# Age and sex specific thresholds for risk stratification of cardiovascular disease and clinical decision making: prospective open cohort study

**DOI:** 10.1136/bmjmed-2023-000633

**Published:** 2024-08-12

**Authors:** Zhe Xu, Juliet Usher-Smith, Lisa Pennells, Ryan Chung, Matthew Arnold, Lois Kim, Stephen Kaptoge, Matthew Sperrin, Emanuele Di Angelantonio, Angela M Wood

**Affiliations:** 1 British Heart Foundation Cardiovascular Epidemiology Unit, Department of Public Health and Primary Care, University of Cambridge, Cambridge, UK; 2 Primary Care Unit, Department of Public Health and Primary Care, University of Cambridge, Cambridge, UK; 3 National Institute for Health and Care Research Blood and Transplant Research Unit in Donor Health and Behaviour, University of Cambridge, Cambridge, UK; 4 Division of Informatics, Imaging and Data Science, Faculty of Biology Medicine and Health, The University of Manchester, Manchester, UK; 5 British Heart Foundation Centre of Research Excellence, University of Cambridge, Cambridge, UK; 6 Health Data Research UK Cambridge, Wellcome Genome Campus and University of Cambridge, Cambridge, UK; 7 Health Data Science Research Centre, Human Technopole, Milan, Italy; 8 Cambridge Centre of Artificial Intelligence in Medicine, Cambridge, UK

**Keywords:** Cardiovascular disease, Risk prediction, Risk stratification, Statin treatment

## Abstract

**Objective:**

To quantify the potential advantages of using 10 year risk prediction models for cardiovascular disease, in combination with risk thresholds specific to both age and sex, to identify individuals at high risk of cardiovascular disease for allocation of statin treatment.

**Design:**

Prospective open cohort study.

**Setting:**

Primary care data from the UK Clinical Practice Research Datalink GOLD, linked with hospital admissions from Hospital Episode Statistics and national mortality records from the Office for National Statistics in England, 1 January 2006 to 31 May 2019.

**Participants:**

1 046 736 individuals (aged 40-85 years) with no cardiovascular disease, diabetes, or a history of statin treatment at baseline using data from electronic health records.

**Main outcome measures:**

10 year risk of cardiovascular disease, calculated with version 2 of the QRISK cardiovascular disease risk algorithm (QRISK2), with two main strategies to identify individuals at high risk: in strategy A, estimated risk was a fixed cut-off value of ≥10% (ie, as per the UK National Institute for Health and Care Excellence guidelines); in strategy B, estimated risk was ≥10% or ≥90th centile of age and sex specific risk distributions.

**Results:**

Compared with strategy A, strategy B stratified 20 241 (149.8%) more women aged ≤53 years and 9832 (150.2%) more men aged ≤47 years as having a high risk of cardiovascular disease; for all other ages the strategies were the same. Assuming that treatment with statins would be initiated in those identified as high risk, differences in the estimated gain in cardiovascular disease-free life years from statin treatment for strategy B versus strategy A were 0.14 and 0.16 years for women and men aged 40 years, respectively; among individuals aged 40-49 years, the numbers needed to treat to prevent one cardiovascular disease event for strategy B versus strategy A were 39 versus 21 in women and 19 versus 15 in men, respectively.

**Conclusions:**

This study quantified the potential gains in cardiovascular disease-free life years when implementing prevention strategies based on age and sex specific risk thresholds instead of a fixed risk threshold for allocation of statin treatment. Such gains should be weighed against the costs of treating more younger people with statins for longer.

**WHAT IS ALREADY KNOWN ON THIS TOPIC**

- Risk prediction models for cardiovascular disease are recommended to identify people at high risk of cardiovascular disease and who might benefit the most from preventive interventions
- There is a concern that for younger individuals, especially those with a heavy burden of risk factors for cardiovascular disease, the benefit of statin treatment is delayed until their estimated risks are above a prespecified single threshold

**WHAT THIS STUDY ADDS**

- Based on data from the electronic health records of 1 046 736 individuals collected in UK primary care setting, two risk stratification strategies to identify individuals at high risk of cardiovascular disease were assessed: strategy A, estimated risk ≥10% (ie, according to guidelines from the UK National Institute for Health and Care Excellence); strategy B, estimated risk ≥10% or ≥90th centile of age and sex specific risk distributions
- In men and women aged 40 years, compared with a fixed risk threshold, use of age and sex specific thresholds was associated with a modest increase in the gain in cardiovascular disease-free life years from statin treatment of 0.14-0.16 years, and a slightly higher number needed to treat to prevent one cardiovascular disease event
- No differences between the strategies were found in older individuals

**HOW THIS STUDY MIGHT AFFECT RESEARCH, PRACTICE, OR POLICY**

- Using age and sex specific thresholds in combination with 10 year cardiovascular disease risk prediction models can modestly enhance risk stratification of cardiovascular disease for allocating statin treatment, especially among men and women at earlier ages
- The benefits need to be formally weighted against the costs of treating more younger people with statins for longer

## Introduction

 Cardiovascular disease remains the leading cause of morbidity and mortality, as well as a main contributor to disability globally.^1 2^ Identifying individuals who are at higher risk of cardiovascular disease is essential for effectively allocating interventions for primary prevention of cardiovascular disease. For this purpose, risk prediction models for cardiovascular disease have been developed and recommended in clinical practice guidelines worldwide.^3^,^15^ These prediction models can inform decision making about allocating preventive interventions, such as lifestyle modification and statin treatment, for individuals with an increased risk of cardiovascular disease.

Although clinical practice guidelines recommend different risk prediction models and corresponding risk thresholds, a common feature is the use of a single risk threshold for the overall population to identify people at high risk of cardiovascular disease.^6 9 12 13^ For example, guidelines from the UK National Institute for Health and Care Excellence (NICE), published in 2014,^6^ and the American College of Cardiology/American Heart Association, published in 2019,^13^ recommend identifying individuals at high risk as those with an estimated fatal or non-fatal 10 year risk of cardiovascular disease ≥10% and ≥7.5%, respectively. More recently, the updated 2023 NICE guideline recommends considering statins initiation if a concern exists that the risk could be underestimated.^6^ However, the absolute estimates of the risk of cardiovascular disease are much dependent on age and sex.^7^ For younger individuals, especially women, with a heavy burden of risk factors for cardiovascular disease, using a single risk threshold for the whole population may delay the benefit of statin treatment.^16^ In some guidelines, the estimated risk of lifetime cardiovascular disease for younger adults has been incorporated along with the 10 year risk estimates to aid treatment recommendations.^7 13 15^ No clear cut-off values for identifying a high lifetime risk of cardiovascular disease have been currently established,^17^ however, making it difficult to implement in practice.

As an alternative to lifetime risk prediction models for cardiovascular disease, age specific thresholds in combination with 10 year risk prediction models for cardiovascular disease could be easier to implement.^18^ The 2021 guidelines from the European Society of Cardiology proposed three age dependent risk thresholds for younger, middle aged, and older age groups (ie, ≥7.5% for age <50 years, ≥10% for age 50-69 years, and ≥15% for age ≥70 years).^15 19^ Limited quantitative analysis exists for establishing and assessing the clinical benefits and harms of age and sex specific risk thresholds for cardiovascular disease, with a gap in the evidence for frameworks that can be adapted and implemented across populations.

In this study, our aim was to enhance the quantitative evidence and provide a framework for incorporating risk distributions specific to age and sex into decision making for statins initiation in the primary prevention of cardiovascular disease. We used a large UK primary care electronic health records database to assess the potential clinical benefits and harms of augmenting recommended 10 year risk prediction tools for cardiovascular disease (ie, version 2 of the QRISK cardiovascular disease risk algorithm (QRISK2)^5^ used in the UK and version 2 of the Systematic COronary Risk Evaluation (SCORE2)^15 19^ used across Europe) with thresholds based on the centiles of risk distributions in the population by age and sex.

## Methods

### Study population and data sources

We used primary care data from the Clinical Practice Research Datalink (CPRD) GOLD, linked with hospital admissions from Hospital Episode Statistics (HES) and national mortality records from the Office for National Statistics (ONS) in England. CPRD GOLD contains anonymised individual level primary care records from UK general practitioners, covering about 6.9% of the UK population, and is broadly representative with regards to age, sex, and ethnic group.^20^ Data for sex were taken from information in the CPRD.

Individuals were identified and followed up from the study baseline, which was set as 1 January 2006, allowing an approximate two year time window after 1 April 2004 (the date of introduction of the quality and outcomes framework in the UK NHS^21^), for the recording of measurements of risk factors before baseline until: the date of their first newly recorded cardiovascular disease event or death; their 95th birthday; date of de-registration at the general practice or the last contact date for the practice with CPRD; or 31 May 2019 (the end of data availability), whichever came first. We further restricted the study population to those aged 40-85 years with no previous cardiovascular disease (codelists shown in online supplemental methods 1), and no history of treatment with statins or prevalent diabetes at baseline (because people with type 1 and type 2 diabetes are considered at high risk of cardiovascular disease, regardless of their predicted risks, in the clinical guidelines from NICE and European Society of Cardiology^6 15^). Online supplemental figure 1 shows a flowchart of selection of the study population.

### Statistical analysis

#### Risk estimates for cardiovascular disease

For the primary analyses, we estimated the 10 year risk of cardiovascular disease for each individual using the QRISK2 algorithm,^5^ as recommended in the UK cardiovascular disease risk assessment guideline. Although QRISK3 is recommended in the updated 2023 NICE guideline, until electronic clinical systems in which QRISK2 is embedded are updated with QRISK3, using QRISK2 might be necessary, as indicated in the guideline.^6^ Online supplemental methods 1 provides details of the cardiovascular disease outcomes and risk factors used in the QRISK2 algorithm. Multiple imputation by chained equations was used to impute missing values for smoking status, systolic blood pressure, total cholesterol, high density lipoprotein cholesterol, and body mass index (online supplemental methods 1). We performed five imputations which were adequate to get relatively high efficiency^22^ and were pragmatic for our sample size. Analyses were performed in each imputed dataset separately and then the results were pooled across imputations with Rubin's rules.^22^ External validation of the QRISK2 model in our data involved assessment of overall performance (with the R^2^ statistic^23^), discrimination (with Harrell's C statistic^24^ and D statistic^25^), and calibration (visually assessing the agreement of observed risk and predicted risk by 10ths of predicted risk^26^) (online supplemental methods 1).

#### Risk stratification strategies

With the estimated 10 year risks of cardiovascular disease from the QRISK2 algorithm, individuals were stratified as having a high risk of cardiovascular disease for allocating statins based on two main strategies: in strategy A, predicted risk was a fixed high risk cut-off value of 10% (ie, individuals who had an absolute risk ≥10% were identified as high risk); in strategy B, individuals were first grouped by centiles of predicted risk at each age, by one year age groups, and sex, and then were identified as high risk if they had an absolute risk ≥10% or an estimated risk exceeding the 90th centile of the age and sex specific risk distributions.

The 90th centile was selected as an example to illustrate the potential results of applying age and sex specific thresholds in risk stratification for cardiovascular disease. We applied this approach to lower the thresholds at younger ages rather than to increase the thresholds at older ages, with the consideration that this would be a pragmatic, acceptable, and implementable strategy.

#### Performance of stratification strategies

Although individuals receiving treatment with statins at baseline were excluded, about 20% of included individuals initiated statin treatment during follow-up (so-called treatment drop-ins^27 28^). Ignoring treatment initiation could underestimate the observed risks of cardiovascular disease.^29^ Therefore, we first estimated the counterfactual statin naive survival time, which accounts for the treatment drop-ins effect^30^ (online supplemental methods 2). The counterfactual statin naive survival times were used for the subsequent evaluation of the stratification performance.

To compare the stratification strategies, we calculated sensitivity (ie, proportion of individuals who are correctly grouped as high risk by the stratification strategy^31^), specificity (ie, proportion of individuals who are correctly identified as low risk^31^), an adapted area under the receiver operating characteristic curve for dichotomised predictions (AUROC-dp), and net benefit. AUROC-dp measures the ability to discriminate between individuals who do and do not have a cardiovascular disease event according to the combined risk prediction model and the stratification rule. As a measure of discrimination, AUROC-dp generally has values from 0.5 (representing discriminative ability equal to chance alone) to 1 (when the risk prediction model and stratification strategy perfectly divides individuals into those who do and do not later have a cardiovascular disease event).^32^,^34^

Net benefit was estimated to assess the clinical value of different risk stratification strategies and their clinical consequences. Net benefit represents the difference between the true positive rate and false positive rate weighted by the odds of the selected threshold for being at high risk, with higher values indicating greater net benefit.^35^,^37^ Sensitivity, specificity, AUROC-dp, and net benefit were calculated accounting for censoring. Online supplemental methods 3 and 4 describe the methods in detail.

#### Potential public health impact

We quantified the public health impact of the combined risk prediction model and the stratification rule by the number needed to screen and number needed to treat to prevent one new cardiovascular disease event in 10 years, under the assumption that statin treatment is given to individuals at high risk and reduces the risk of cardiovascular disease. We assumed a 25% relative risk reduction in cardiovascular disease upon statins allocation, for all ages, sexes,^38 39^ and treatment duration,^40^ while allowing for different adherence rates to statin treatment by age and sex (online supplemental methods 5). The number needed to screen will always be smaller when the threshold is lowered, and is at a minimum when everyone is treated. In contrast, the number needed to treat will always increase when the threshold is lowered.

To investigate the long term benefit of treating individuals with a high risk of cardiovascular disease with statins, we estimated the gain in cardiovascular disease-free life expectancy associated with statin initiation by age and sex. Cardiovascular disease-free life expectancy (or life years free of cardiovascular disease) is defined as the average duration of survival without cardiovascular disease over the follow-up period, and was calculated as the area under the cardiovascular disease-free survival curve.^41^ To better reflect the potential benefits over a lifetime, especially for younger individuals with low short term risks and who were expected to survive far longer than the available follow-up time, we used age as the time scale and adjusted for the competing risk of death from non-cardiovascular disease events to assess the potential longer term cardiovascular disease-free survival.^42^ Also, future life years were discounted with a time preference rate of 0.03 (which assumes that the value of the next year is worth 97% of the previous year) to account for a likely increasing lower value that individuals might give to life years further out into the far-off future.^43^ Estimations were based on sex specific life tables combining age specific risks of cardiovascular disease and risks of death from non-cardiovascular disease in one year age intervals.^42^

When individuals were identified as having a high risk of cardiovascular disease at baseline age according to each stratification strategy, the one year risk of cardiovascular disease was calculated by incorporating the relative risk reduction of statin treatment on cardiovascular disease into the subdistribution of risk of cardiovascular disease for each of the remaining life years. The gain in cardiovascular disease-free life years is the difference in cardiovascular disease-free life expectancy with and without treatment of statins assumed. To illustrate the results intuitively at a population level, we calculated the possible gain in cardiovascular disease-free life years in England based on the most recent available data on the age and sex structure of the 2020 mid-year England population aged 40-85 years.^44^ Online supplemental methods 6 provides details of the calculation.

#### Sensitivity analyses

Because the number needed to screen, number needed to treat, and population average gain in cardiovascular disease-free life years from statin treatment depend on the number of individuals identified as high risk, to make a fairer comparison across strategies, we further performed sensitivity analyses by ascertaining the same number of individuals at high risk of cardiovascular disease in each strategy. We constrained the number of individuals classified as having a high risk of cardiovascular disease to be the same as the number identified in strategy B among the whole population sample, and then identified the corresponding single risk threshold as an alternative fixed threshold for strategy A. This single risk threshold was identified to be 9.2% (strategy A1).

Sensitivity analyses were also conducted with the SCORE2^14^ and SCORE2-OP^45^ algorithms, as recommended in the current guidelines from European Society of Cardiology (with the low risk region equations for the UK population as recommended).^15^ Online supplemental methods 1 provides details of the cardiovascular disease outcomes and risk factors used in the SCORE2 and SCORE2-OP algorithms. We further assessed age specific risk stratification thresholds with high risk cut-off values at 7.5%, 10%, or 15% for younger (40-49 years), middle aged (50-69 years), and older (≥70 years) age groups, respectively, as recommended in the European Society of Cardiology guidelines (strategy C). Thus, for risk estimates based on SCORE2, we compared the stratification performance of strategy A (single 10% threshold), strategy B (age and sex specific thresholds), and strategy C (age specific thresholds).

Analyses were performed with Stata version 15.1 (StataCorp, College Station, TX, USA) and R version 3.6.1 (R Foundation for Statistical Computing, Vienna, Austria). The manuscript was prepared in accordance with the strengthening the reporting of observational studies in epidemiology (STROBE) statement (online supplemental material).

### Patient and public involvement

Patients and the public were not involved in the design, or conduct, or reporting, or dissemination plans of this research, mainly because this study used anonymised electronic health records data and focused on population level results. We plan to communicate the study findings to stakeholders related to cardiovascular disease guidelines.

## Results

### Characteristics of the study population

The analyses included 1 046 736 eligible individuals. Table 1 summarises the characteristics of the study participants at baseline. Mean age at baseline was 55 years (standard deviation (SD) 11) in men and 57 years (SD 12) in women. Online supplemental table 1 provides summaries of the observed and imputed risk factor data. Excluded individuals with a history of statin treatment at baseline (n=80 860) were generally older (mean age at baseline 65 years (SD 10)) than individuals without statin treatment at baseline (n=1 046 736) (online supplemental tables 2 and 3). The proportion of individuals with statin treatment at baseline was low among younger individuals (online supplemental table 3), with an average proportion of 3.8% among people aged 40-60 years. A total of 80 569 incident cardiovascular disease events were identified during a median follow-up period of 7.8 years (5th, 95th centiles 0.9, 13.4; online supplemental figure 2), with an incidence rate of 10.4 (95% confidence interval (CI) 10.3 to 10.5) per 1000 person years.

**Table 1:** Baseline characteristics of individuals included in risk estimation by version 2 of the QRISK cardiovascular disease risk algorithm (QRISK2)*

Characteristics†	Dataset for QRISK2 estimation (n=1 046 736)	
	Men (n=498 687, 47.6%)	Women (n=548 049, 52.4%)
Mean (SD) age at baseline (years)	55.2 (11.1)	57.0 (12.1)
Mean (SD) systolic blood pressure (mm Hg)	134.7 (15.5)	131.6 (17.1)
Mean (SD) total cholesterol (mmol/L)	5.5 (1.1)	5.6 (1.1)
Mean (SD) high density lipoprotein cholesterol (mmol/L)	1.4 (0.4)	1.7 (0.4)
Mean (SD) total to high density lipoprotein cholesterol ratio	4.3 (1.3)	3.6 (1.1)
Mean (SD) body mass index	27.5 (4.7)	27.1 (5.8)
Current or ever smoker	217 233 (43.6)	232 116 (42.4)
Ethnic group:		
White/not recorded	490 492 (98.4)	537 384 (98.1)
Indian	1845 (0.4)	2556 (0.5)
Pakistani	598 (0.1)	682 (0.1)
Chinese	396 (0.1)	591 (0.1)
Bangladeshi	183 (<0.1)	143 (<0.1)
Other Asian	795 (0.2)	1158 (0.2)
Black Caribbean	1171 (0.2)	1661 (0.3)
Black African	891 (0.2)	1076 (0.2)
Other	2316 (0.5)	2798 (0.5)
Prescription for antihypertensive drugs	89 336 (17.9)	156 893 (28.6)
Chronic renal disease	720 (0.1)	946 (0.2)
Atrial fibrillation	6687 (1.3)	5696 (1.0)
Rheumatoid arthritis	3396 (0.7)	9157 (1.7)
Family history of coronary heart disease	15 606 (3.1)	21 845 (4.0)

Data are number (%) unless stated otherwise.

* Variables for age, sex, ethnic group, smoking status, systolic blood pressure, treatment for hypertension, total to high density lipoprotein cholesterol ratio, body mass index, diabetes status, chronic renal disease status, atrial fibrillation, rheumatoid arthritis, Townsend deprivation score (not shown), and family history of cardiovascular disease were used in QRISK2 risk estimation.

† Values for systolic blood pressure, total cholesterol, high density lipoprotein cholesterol, body mass index, and smoking status were estimated based on the pooled results from five imputed datasets.

SD, standard deviation.

### Predicted risk with QRISK2

The mean predicted 10 year risk of cardiovascular disease risk with QRISK2 was 11.4% (online supplemental figure 3) but varied substantially by age and sex (online supplemental figures 4 and 5). For younger people (aged 40-49 years), only 1.2% of women and 5.6% of men had a predicted QRISK2 risk ≥10%, whereas for older people (aged ≥60 years), 83.1% of women and 99.0% of men had a predicted risk ≥10%.

The QRISK2 model was well calibrated for both men and women (online supplemental figures 6 and 7) and had generally good predictive ability, with an overall R^2^ of 38.5 (95% CI 38.1 to 38.9), Harrell's C statistic of 0.772 (0.770 to 0.773), and D statistic of 1.619 (1.604 to 1.633). Online supplemental table 4 presents the sex specific results. These measures of model predictive performance were comparable with the results reported in the original QRISK2 validation research.^5^

### Assessment of risk stratification strategies

#### Thresholds for risk stratification strategies

Table 2 shows the number and proportion of people identified as having a high risk of cardiovascular disease for each risk stratification strategy. Compared with strategy A, strategy B stratified 20 241 (149.8%) more women aged ≤53 years and 9832 (150.2%) more men aged ≤47 years as having a high risk of cardiovascular disease; for all other ages the strategies were the same. For strategy B, individuals with a predicted risk ≥90th centile had higher values for systolic blood pressure, total cholesterol, and body mass index, and were more likely to be smokers than those with a predicted risk <90th centile (online supplemental table 5).

**Table 2:** Age and sex specific cut-off values for stratifying individuals at high risk of cardiovascular disease, and numbers of individuals identified as high risk in different strategies with risk estimations by version 2 of the QRISK cardiovascular disease risk algorithm (QRISK2)

Age (years)	Men				Women			
	Strategy A*		Strategy B†		Strategy A*		Strategy B†	
	Cut-off (%)	No (%)	Cut-off (%)	No (%)	Cut-off (%)	No (%)	Cut-off (%)	No (%)
40	10	300 (1.3)	5.0	2234 (10.0)‡	10	81 (0.4)	3.0	2127 (10.0)‡
41	10	406 (1.8)	5.5	2204 (10.0)‡	10	99 (0.5)	3.3	2143 (10.0)‡
42	10	490 (2.3)	6.0	2162 (10.0)‡	10	121 (0.6)	3.6	2063 (10.0)‡
43	10	627 (3.0)	6.7	2073 (10.0)‡	10	151 (0.8)	4.0	2012 (10.0)‡
44	10	778 (3.8)	7.3	2023 (10.0)‡	10	198 (1.0)	4.4	1975 (10.0)‡
45	10	1010 (5.1)	8.1	1962 (10.0)§	10	224 (1.2)	4.7	1918 (10.0)‡
46	10	1283 (6.8)	8.8	1886 (10.0)§	10	264 (1.4)	5.2	1824 (10.0)‡
47	10	1653 (9.0)	9.7	1834 (10.0)§	10	338 (1.9)	5.7	1818 (10.0)‡
48	10	2059 (11.7)	10	2059 (11.7)	10	414 (2.4)	6.2	1754 (10.0)‡
49	10	2509 (15.0)	10	2509 (15.0)	10	475 (2.8)	6.6	1685 (10.0)‡
50	10	3150 (19.9)	10	3150 (19.9)	10	566 (3.6)	7.2	1583 (10.0)‡
51	10	3806 (24.6)	10	3806 (24.6)	10	723 (4.5)	7.8	1590 (10.0)§
52	10	4764 (31.0)	10	4764 (31.0)	10	872 (5.5)	8.3	1586 (10.0)§
53	10	5694 (38.1)	10	5694 (38.1)	10	1022 (6.7)	8.9	1535 (10.0)§
54	10	6700 (46.6)	10	6700 (46.6)	10	1330 (8.8)	10	1507 (10.0)
55	10	7878 (54.9)	10	7878 (54.9)	10	1702 (11.3)	10	1702 (11.3)
56	10	9242 (63.2)	10	9242 (63.2)	10	2132 (13.9)	10	2132 (13.9)
57	10	10 735 (71.6)	10	10 735 (71.6)	10	2796 (17.6)	10	2796 (17.6)
58	10	13 024 (79.7)	10	13 024 (79.7)	10	3807 (21.7)	10	3807 (21.7)
59	10	12 600 (86.9)	10	12 600 (86.9)	10	4294 (27.3)	10	4294 (27.3)
60	10	10 711 (92.7)	10	10 711 (92.7)	10	4174 (33.5)	10	4174 (33.5)
61	10	11 897 (96.7)	10	11 897 (96.7)	10	5455 (41.2)	10	5455 (41.2)
62	10	10 577 (98.8)	10	10 577 (98.8)	10	6115 (50.6)	10	6115 (50.6)
63	10	9943 (99.7)	10	9943 (99.7)	10	6607 (60.1)	10	6607 (60.1)
64	10	8296 (99.9)	10	8296 (99.9)	10	6593 (69.4)	10	6593 (69.4)
65	10	7843 (100.0)	10	7843 (100.0)	10	7225 (78.6)	10	7225 (78.6)
66	10	7740 (100.0)	10	7740 (100.0)	10	8159 (87.4)	10	8159 (87.4)
67	10	7489 (100.0)	10	7489 (100.0)	10	8330 (93.8)	10	8330 (93.8)
68	10	6980 (100.0)	10	6980 (100.0)	10	8255 (97.8)	10	8255 (97.8)
69	10	6421 (100.0)	10	6421 (100.0)	10	8036 (99.6)	10	8036 (99.6)
70	10	5823 (100.0)	10	5823 (100.0)	10	7967 (99.9)	10	7967 (99.9)
71	10	5550 (100.0)	10	5550 (100.0)	10	7417 (100.0)	10	7417 (100.0)
72	10	5044 (100.0)	10	5044 (100.0)	10	6912 (100.0)	10	6912 (100.0)
73	10	4837 (100.0)	10	4837 (100.0)	10	6884 (100.0)	10	6884 (100.0)
74	10	4670 (100.0)	10	4670 (100.0)	10	6689 (100.0)	10	6689 (100.0)
75	10	4488 (100.0)	10	4488 (100.0)	10	6710 (100.0)	10	6710 (100.0)
76	10	4077 (100.0)	10	4077 (100.0)	10	6313 (100.0)	10	6313 (100.0)
77	10	3883 (100.0)	10	3883 (100.0)	10	6019 (100.0)	10	6019 (100.0)
78	10	3483 (100.0)	10	3483 (100.0)	10	5642 (100.0)	10	5642 (100.0)
79	10	3316 (100.0)	10	3316 (100.0)	10	5325 (100.0)	10	5325 (100.0)
80	10	3119 (100.0)	10	3119 (100.0)	10	5162 (100.0)	10	5162 (100.0)
81	10	2816 (100.0)	10	2816 (100.0)	10	5019 (100.0)	10	5019 (100.0)
82	10	2663 (100.0)	10	2663 (100.0)	10	4939 (100.0)	10	4939 (100.0)
83	10	2290 (100.0)	10	2290 (100.0)	10	4747 (100.0)	10	4747 (100.0)
84	10	2221 (100.0)	10	2221 (100.0)	10	4565 (100.0)	10	4565 (100.0)
85	10	2137 (100.0)	10	2137 (100.0)	10	4558 (100.0)	10	4558 (100.0)

* Strategy A identified individuals with a high risk of cardiovascular disease as those with an estimated 10 year risk of cardiovascular disease ≥10% (fixed threshold).

† Strategy B identified individuals with a high risk of cardiovascular disease as those with an estimated 10 year risk of cardiovascular disease ≥10% or ≥90th centile of age and sex specific risk distributions (age and sex specific thresholds).

‡ Cut-off values <7.5%.

§ Cut-off values ≥7.5% and <10%.

#### Sensitivity, specificity, AUROC-dp, and net benefit

The overall sensitivity and specificity were 79.6% and 63.8%, respectively, for strategy A, and 81.3% and 60.8%, respectively, for strategy B. Corresponding sex specific values for sensitivity and specificity were 78.0% and 70.0% in women and 80.4% and 56.8% in men for strategy A, and 80.1% and 66.2% in women and 81.7% and 54.7% in men for strategy B (online supplemental table 6). Strategy B provided higher sensitivity with only modest reductions in specificity for individuals at younger ages (online supplemental table 7 and online supplemental figure 8). For older individuals aged 70-85 years, we found no difference in sensitivity and specificity for the two strategies because all people were classified as having a high risk of cardiovascular disease.

The overall AUROC-dp was about 0.7 for strategies A and B (online supplemental table 6). Among those aged 40-49 years, however, strategy B had higher discrimination: AUROC-dp was 0.555 (95% CI 0.550 to 0.559) for strategy A and 0.583 (0.577 to 0.589) for strategy B) (online supplemental table 8 and online supplemental figure 9). For older individuals aged 70-85, we found no difference in AUROC-dp between the two strategies because all people were classified as having a high risk of cardiovascular disease.

**Figure 1:**
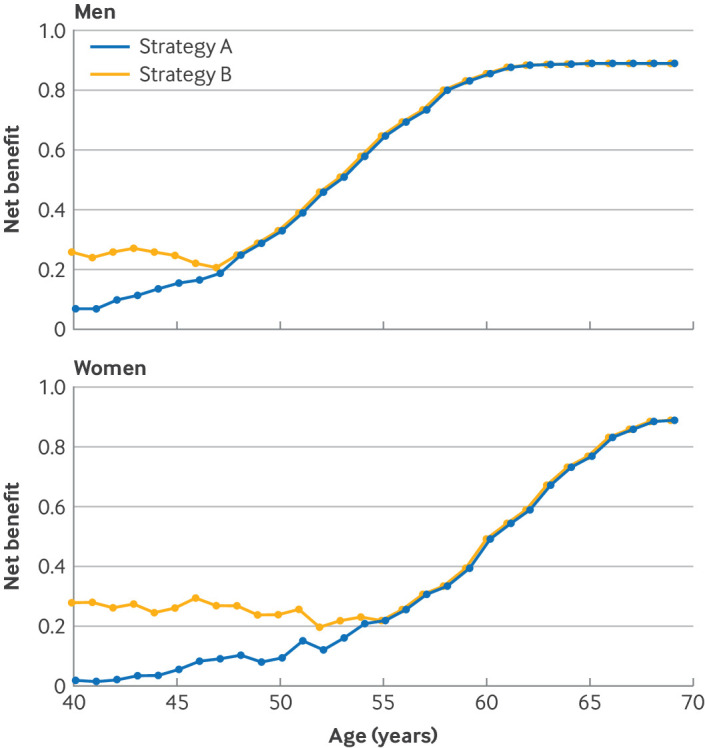
Net benefit for different stratification strategies by age for men and women, with risk estimations by QRISK cardiovascular disease risk algorithm (QRISK2). Strategy A identified individuals with a high risk of cardiovascular disease as those with an estimated 10 year risk of cardiovascular disease ≥10% (fixed threshold). Strategy B identified individuals with a high risk of cardiovascular disease as those with an estimated 10 year risk of cardiovascular disease ≥10% or ≥90th centile of age and sex specific risk distributions (age and sex specific thresholds)

For men aged 40-47 years and women aged 40-54 years, the net benefit was relatively constant and equal across age and sex, and higher for strategy B than strategy A (figure 1). For example, the net benefit was 0.24 for strategy B versus 0.10 for strategy A for women aged 50 years (figure 1).

#### Estimated number needed to screen and treat to prevent one cardiovascular disease event

**Figure 2:**
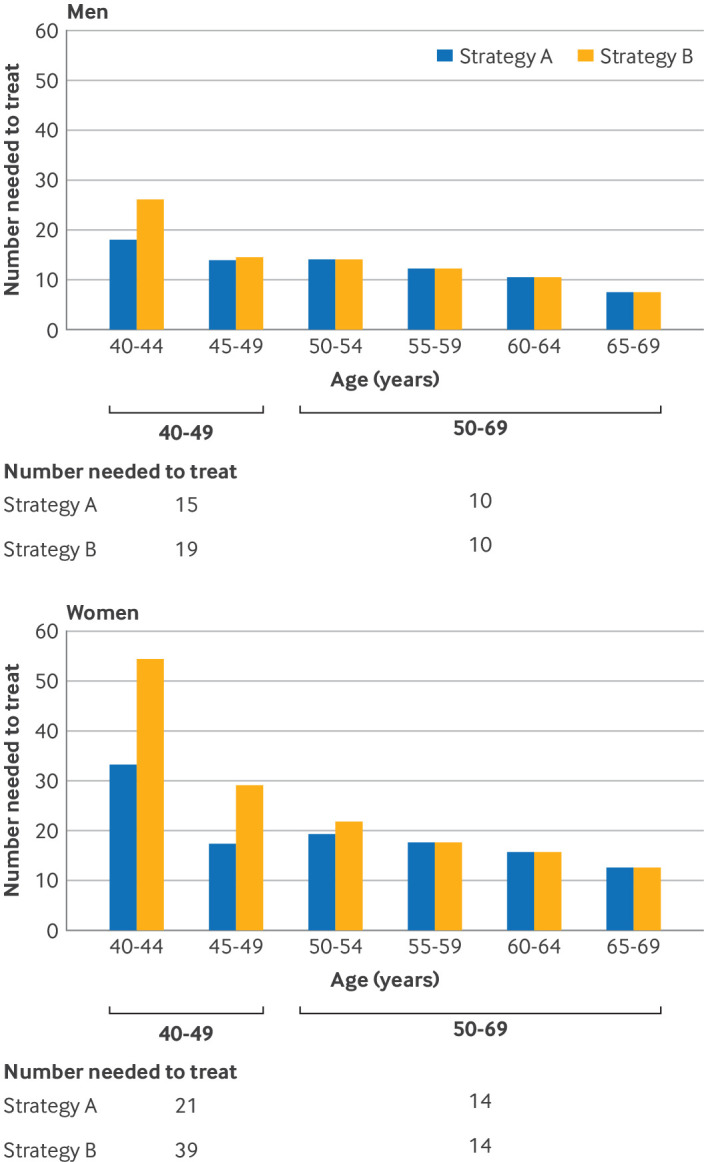
Estimated number needed to treat to prevent one new cardiovascular disease event in different stratification strategies by five year age groups for men and women, with risk estimations by version 2 of the QRISK cardiovascular disease risk algorithm (QRISK2). Strategy A identified individuals with a high risk of cardiovascular disease as those with an estimated 10 year risk of cardiovascular disease ≥10% (fixed threshold). Strategy B identified individuals with a high risk of cardiovascular disease as those with an estimated 10 year risk of cardiovascular disease ≥10% or ≥90th centile of age and sex specific risk distributions (age and sex specific thresholds)

The number needed to screen was substantially lower for people aged <50 years for strategy B than strategy A (online supplemental figure 10), with only a modest increase in the number needed to treat (figure 2). For example, in individuals aged 40-49 years, the overall number needed to screen was 494 versus 259; the sex specific number needed to screen was 1667 versus 398 in women and 263 versus 178 in men, respectively. In contrast, the overall number needed to treat was 17 versus 26; the sex specific number needed to treat was 21 versus 39 in women and 15 versus 19 in men, respectively (figure 2).

#### Gain in cardiovascular disease-free life expectancy from statin treatment

**Figure 3:**
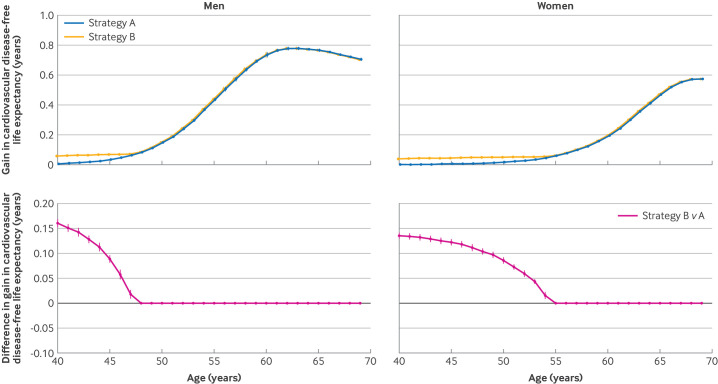
Population average gain in cardiovascular disease-free life years from treatment with statins in the high risk population and difference in the gain comparing different stratification strategies, with risk estimations by version 2 of the QRISK cardiovascular disease risk algorithm (QRISK2). Results are shown as population average gain in cardiovascular disease-free life years and difference in the gain between strategy B versus strategy A in men and women with 95% confidence intervals. Strategy A identified individuals with a high risk of cardiovascular disease as those with an estimated 10 year risk of cardiovascular disease ≥10% (fixed threshold). Strategy B identified individuals with a high risk of cardiovascular disease as those with an estimated 10 year risk of cardiovascular disease ≥10% or ≥90th centile of age and sex specific risk distributions (age and sex specific thresholds)

Strategy B had a modest increase in the average gain in cardiovascular disease-free life years (from statin treatment compared with no statin treatment) versus strategy A in younger individuals (figure 3); the maximum difference in the average gain was 0.14 years in women and 0.16 years in men at age 40 years. Standardising to the mid-year population of England in 2020, the expected population gains in cardiovascular disease-free life expectancy were similar (online supplemental figure 11).

#### Sensitivity analyses

When modelling a fixed budget scenario, constraining the total number of individuals stratified as having a high risk of cardiovascular disease among the whole population sample to be the same across strategies, a single threshold of 9.2% (strategy A1) was identified to ascertain the same number of high risk individuals as that from strategy B among all individuals. Use of age and sex specific thresholds remained favourable compared with a single threshold for individuals aged 40-49, with an overall smaller number needed to screen and only a slightly higher number needed to treat (online supplemental table 9). The maximum difference in the average gain in cardiovascular disease-free life years for strategy B versus strategy A1 was 0.13 years in women and 0.15 years in men at age 40 years (online supplemental figure 12). For women aged 54-73 years and men aged 47-67 years, the gain in cardiovascular disease-free life years was smaller with strategy B than strategy A1, because more individuals were selected as high risk based on the 9.2% threshold in strategy A1 versus the 10% threshold in strategy B for those age groups.

Online supplemental tables 10-12 and online supplemental figures 13-18 present supplementary results from similar analyses but with predicted 10 year risk of cardiovascular disease by the SCORE2 (and SCORE2-OP) algorithms. We found similar patterns for comparison of risk stratification performance for SCORE2. Furthermore, the results showed that the intermediate pragmatic approach of age specific thresholds recommended by the European Society of Cardiology guidelines^15^ (ie, 7.5%, 10%, or 15% for younger (40-49 years), middle aged (50-69 years), and older (≥70 years) age groups) (strategy C) produced improvement over a single threshold, although to a lesser degree than with the age and sex specific stratification strategy (online supplemental tables 13-18 and online supplemental figures 19-25).

## Discussion

### Principal findings

In this study, we quantified the use of age and sex specific risk thresholds in combination with 10 year risk prediction models for cardiovascular disease for guiding clinical decisions for starting treatment with statins. Risk thresholds equal to the 90th centiles of age and sex specific risk distributions stratified more people at younger ages (women aged ≤53 years and men aged ≤47 years) as having a high risk of cardiovascular disease to statin initiation compared with a fixed 10% threshold. This result translates to a moderate gain of 0.14-0.16 cardiovascular disease-free life years from earlier statin treatment for those aged 40 years because of the longer benefits from statin treatment given to the younger high risk population, and a modest increase in the number needed to treat from 21 to 39 in women and from 15 to 19 in men, at ages 40-49 years.

Extensions and alternative approaches merit consideration. For example, other potential strategies are ones that achieve same sensitivity, false negative rates (eg, fixed 5% false negative rate),^46^ or net benefit^35^ across different ages for men and women. It is noteworthy that in our study, risk thresholds equal to the 90th centiles of age and sex specific risk distributions resulted in approximately equal estimates across younger ages and sex for sensitivity, specificity, and net benefit. Risk thresholds could be further specified by ethnic group and other metrics of social and economic status, which might have important implications for the fairness of risk assessments beyond age and sex.^47 48^ Alternatively, individuals could be stratified by their potential impact of treatment, which incorporate causal effects of modification of risk factors on risk of disease and disease-free life years (eg, the JBS3 risk calculator^7^). Regardless, we have highlighted the importance of ensuring changes to thresholds align with a clinically sensible balance between benefits and harms.

### Study implications

Our study emphasises the important role of age and sex in risk stratification for cardiovascular disease and provides a framework of using information from age and sex specific risk distributions to support decision making on statin initiation for primary prevention of cardiovascular disease. Also, we found that using both age and sex showed better stratification performance than merely using age specific thresholds. As suggested in guidelines, risk assessment and stratification are not the only determinants of allocating statins in clinical practice, but should be used as a starting point for an informed discussion between clinicians and patients,^6^ and to motivate patients to adhere to statin treatment. With the steady increases in risk algorithms for cardiovascular disease into electronic healthcare systems,^49^ further incorporating age and sex specific thresholds to stratify high risk individuals is a relatively straightforward extension to implement.

Better personalising of statin treatment towards people at high risk of cardiovascular disease earlier supports prevention strategies, leading to fewer incident cardiovascular diseases, however longer use of statins might raise concerns about safety, cost, and adherence. Evidence from meta-analyses of clinical trials^50^ and observational studies^51^ suggests small absolute excess harm of statins, and microsimulation models in the US and Scottish populations indicated improved cost effectiveness with lower risk thresholds for cardiovascular disease.^16 52 53^ The evidence is not consistent across studies,^54^ however, and more research on the use of age and sex specific thresholds in cost effectiveness analyses in different populations with limited health resources is warranted. Subsequent efforts on improving adherence to long term statin treatment are important. Moreover, extending the awareness of age and sex specific thresholds to inform the implementation of other preventive interventions, such as health education, lifestyle modification, and treatment of hypertension, is also possible.^6 55^ This approach is important for targeting an overall risk reduction in cardiovascular disease rather than focusing on a reduction in cholesterol levels only, as has been recently re-emphasised in guidelines for the prevention of cardiovascular disease.^56^

In this study, we focused on risk thresholds for 10 year risk estimations for cardiovascular disease, as commonly recommended in clinical practice. In some guidelines, assessment of the 30 year or lifetime risk of cardiovascular disease has been incorporated along with the traditional 10 year risk estimates in treatment recommendations.^7 13 15^ We note the similar goals in using lifetime risk prediction models for cardiovascular disease and using age specific thresholds; both facilitate age specific clinical decision making. For example, the lifetime risk for an individual aged 40 years is the estimated risk of cardiovascular disease over the next 55 years to age 95 years, whereas the lifetime risk for a person aged 70 years is the estimated risk of cardiovascular disease over the next 25 years. Likewise, age specific thresholds in combination with existing recommended 10 year risk prediction models for cardiovascular disease are likely to be easier to implement in practice compared with new models for lifetime risk of cardiovascular disease.

### Strengths and limitations of this study

Our study had several strengths. To the best of our knowledge, our work is the first to provide quantitative evidence of using age and sex specific risk thresholds for cardiovascular disease for allocating statins in the UK population. This study included data from more than one million individuals with >80 000 incident cardiovascular disease events identified from large and representative UK population electronic health records. This large sample therefore increases the statistical power to detect any meaningful differences in risk stratification performance. Contemporary data collected from 2004 to 2019, with linked information on primary care, hospital admissions, and national mortality records were used. Multiple imputation by chained equations was implemented to impute missing values for risk factors to reduce bias from restricting the study population to those with complete data. We accept that the possibility of some missing values being missing not at random remains, especially among people who do not engage with healthcare for reasons that are difficult to measure; however, the impact of this limitation is likely to be small, given the large sample size and the large number of covariates used in the imputation models.

Additionally, we assessed the risk of cardiovascular disease with the QRISK2 algorithm, which is recommended in the UK guideline, and also the SCORE2 algorithms, which are recommended in the latest European guidelines. Both risk prediction models showed good predictive performance, with similar findings for stratification comparisons with both scores. Moreover, to provide evidence for supporting the decision making on statin initiation in a statin naive population, we excluded individuals who were receiving statin treatment at baseline. Potential risks of selection bias caused by the difference in individuals with and without a history of treatment with statins might be negligible because only a small proportion (3.8%) of individuals aged <60 years had a history of statin treatment at baseline. This potential bias would have little impact on the predicted risk distributions, the cut-off values for strategy B, and the assessments of the comparison between risk stratification strategies for younger individuals. We also accounted for statin initiation during the follow-up period by estimating a counterfactual survival time assuming statins had not been started.^30^ We evaluated multiple stratification performance metrics for comparison, which not only included sensitivity and specificity,^18^ but also the potential public health impact and lifetime benefits in cardiovascular disease-free life expectancy. The results of each metric are in agreement with several independent studies.^15^,^1840^

Our study had some limitations. We assumed a constant effect of statins, and age and sex specific adherence rates to statin treatment. Trial based meta-analyses, however, suggest that the statin effect is relatively independent of age and sex^38 39^ but increases with length of treatment.^38 57^ Such finding might lead to an overestimation of performance measures in individuals with a shorter length of statin treatment over their lifespans (ie, older individuals), or an underestimation of performance measures in younger individuals who could benefit from statins for a longer time. Furthermore, recent studies have shown that the proportion of people adhering to statins decreases over time (eg, 76% at six months *v* 51% at five years).^58 59^ Although we considered different adherence rates by age and sex, we assumed that the rates did not vary with time, which might result in an overestimation of the long term performance measures in individuals who are more likely to discontinue statin treatment (eg, men and younger people). Also, differences in other characteristics (eg, cholesterol levels) could affect the actual benefits of statins in individuals.^60^

We acknowledge that the absolute values of thresholds estimated from our study (table 2) might need to be recalibrated for other populations. Nevertheless, our study provides a valuable insight into the framework of using population level risk estimates for cardiovascular disease to inform age and sex specific thresholds and risk stratification. We also assumed that statins were given to all people with a risk above the set thresholds, and did not incorporate more personalised clinical decisions which might take into account existing comorbidities and medication use.

### Conclusions

This study highlights the importance and benefits of using age and sex specific thresholds in combination with existing 10 year risk prediction models for risk stratification for cardiovascular disease. Implementing these strategies into clinical practices should be straightforward. Our results provide quantitative evidence for the potential gains in cardiovascular disease-free life years when using age and sex specific risk thresholds rather than a fixed risk threshold. Such gains should be weighed against the costs of treating more younger people with statins for longer.

## Supplementary material

10.1136/bmjmed-2023-000633 online supplemental file 1

## Data Availability

Data are available upon reasonable request.
