# Supplementary material for: Age and sex specific thresholds for risk stratification of cardiovascular disease and clinical decision making: prospective open cohort study
Source: BMJ Med. 2024 Aug 12;3(1):e000633. doi: 10.1136/bmjmed-2023-000633 (PMC11340247; doi:10.1136/bmjmed-2023-000633)
Supplement: online supplemental file 1 [file bmjmed-3-1-s001.pdf]

Age- and sex-specific thresholds for cardiovascular disease risk stratification and clinical decision-making

Zhe Xu, Juliet Usher-Smith, Lisa Pennells, Ryan Chung, Matthew Arnold, Lois Kim, Stephen Kaptoge, Matthew Sperrin, Emanuele Di Angelantonio, Angela M. Wood

Contents

Supplementary Tables and Figures for QRISK2 risk estimations.......... 2

Results using SCORE2 risk estimations.......... 23

Characteristics of the study population.......... 23

Predicted risk using SCORE2.......... 27

Thresholds for risk stratification strategies.......... 32

Sensitivity, specificity, AUROC-dp, and net benefit.......... 37

Estimated numbers needed to screen (NNS) and treat (NNT) to prevent one CVD event.......... 43

Gain in CVD-free life-expectancy from statin treatment.......... 45

Sensitivity analyses.......... 48

Supplementary Methods 1: Estimation of QRISK2 and SCORE2 predictions using CPRD data.......... 51

Supplementary Methods 2: The closed form calculation of counterfactual survival times in the absence of statin initiation.......... 54

Supplementary Methods 3: Calculation of sensitivity, specificity, and AUROC-dp accounting for censoring.......... 56

Supplementary Methods 4: Estimation of net benefit for different risk stratification strategies.......... 57

Supplementary Methods 5: Calculation of the public health modeling metrics.......... 58

Supplementary Methods 6: Estimation of gain in CVD-free life expectancy from statin treatment.......... 59

STROBE Statement - checklist of items that should be included in reports of observational studies.......... 62

References.......... 66

Supplementary Tables and Figures for QRISK2 risk estimations

Supplementary Table 1. Number of individuals with records for the key risk factors and the comparison for observed values (before imputation) and imputed values of individuals included for QRISK2 estimations

**Characteristics**	**Dataset for QRISK2 estimation (N = 1,046,736)**	
	**Men (n = 498,687, 47.6%)**	**Women (n = 548,049, 52.4%)**
Number of persons with SBP value, n (%)	226,142 (45.3)	339,674 (62.0)
SBP (before imputation), mean (SD), mmHg	136.4 (15.8)	133.0 (17.4)
SBP (after imputation), mean (SD), mmHg	134.7 (15.5)	131.6 (17.1)
Number of persons with total cholesterol value, n (%)	97,875 (19.6)	119,344 (21.8)
Total cholesterol (before imputation), mean (SD), mmol/L	5.5 (1.0)	5.8 (1.0)
Total cholesterol (after imputation), mean (SD), mmol/L	5.5 (1.1)	5.6 (1.1)
Number of persons with HDL cholesterol value, n (%)	74,471 (14.9)	90,479 (16.5)
HDL cholesterol (before imputation), mean (SD), mmol/L	1.4 (0.4)	1.7 (0.4)
HDL cholesterol (after imputation), mean (SD), mmol/L	1.4 (0.4)	1.7 (0.4)
Number of persons with BMI value, n (%)	100,603 (20.2)	163,478 (29.8)
BMI (before imputation), mean (SD), kg/m^2	27.7 (4.8)	27.4 (6.0)
BMI (after imputation), mean (SD), kg/m^2	27.5 (4.7)	27.1 (5.8)
Number of persons with smoking status value, n (%)	116,467 (23.4)	126,119 (23.0)
Current/Ever smoker (before imputation), n (%)	47,606 (40.9)	54,846 (43.5)
Current/Ever smoker (after imputation), n (%)	217,233 (43.6)	23,2116 (42.4)

Abbreviations: BMI, body mass index; HDL, high-density lipoprotein; SBP, systolic blood pressure; SD, standard deviation.

Supplementary Table 2. Baseline characteristics of included individuals by statin treatment status at baseline

Characteristics [a]	With statin treatment at baseline (n = 80,860, 7.2%)	Without statin treatment at baseline (n = 1,046,736, 92.8%)	P-value
Age at baseline, mean (SD), year	64.8 (10.1)	56.2 (11.7)	<0.001
Women, n (%)	42,281 (52.3)	548,049 (52.4)	0.71
Number of persons with SBP value, n (%)	73,144 (90.5)	565,816 (54.1)	
SBP, mean (SD), mmHg	139.4 (15.8)	134.4 (16.9)	<0.001
Number of persons with total cholesterol value, n (%)	70,066 (86.7)	217,219 (20.8)	
Total cholesterol, mean (SD), mmol/L	5.2 (1.2)	5.6 (1.0)	<0.001
Number of persons with HDL cholesterol value, n (%)	57,191 (70.7)	164,950 (15.8)	
HDL cholesterol, mean (SD), mmol/L	1.5 (0.4)	1.5 (0.4)	<0.001
Number of persons with total/HDL cholesterol ratio value, n (%)	57,098 (70.6)	164,277 (15.7)	
Total/HDL cholesterol ratio, mean (SD)	3.7 (1.2)	4.0 (1.2)	<0.001
Number of persons with BMI value, n (%)	38,095 (47.1)	264,081 (25.2)	
BMI, mean (SD), kg/m2	28.5 (5.1)	27.5 (5.6)	<0.001
Number of persons with smoking status value, n (%)	33,121 (41.0)	242,586 (23.2)	
Current/Ever smoker, n (%)	11,042 (33.3%)	102,452 (42.2%)	<0.001
Ethnicity, n (%)			<0.001
White/not recorded	79,302 (98.1)	1,027,876 (98.2)	
Indian	444 (0.5)	4401 (0.4)	
Pakistani	101 (0.1)	1280 (0.1)	
Chinese	49 (0.1)	987 (0.1)	
Bangladeshi	25 (<0.1)	326 (<0.1)	
Other Asian	234 (0.3)	1953 (0.2)	
Black Caribbean	189 (0.2)	2832 (0.3)	
Black African	98 (0.1)	1967 (0.2)	
Other	418 (0.5)	5114 (0.5)	
Prescription for antihypertensive medication, n (%)	58,472 (72.3)	246,229 (23.5)	<0.001
Chronic renal disease, n (%)	768 (0.9)	1666 (0.2)	<0.001
Atrial fibrillation, n (%)	3374 (4.2)	12383 (1.2)	<0.001
Rheumatoid arthritis, n (%)	1355 (1.7)	12553 (1.2)	<0.001
Family history of CHD, n (%)	6442 (8.0)	37451 (3.6)	<0.001

Abbreviations: BMI, body mass index; HDL, high-density lipoprotein; SBP, systolic blood pressure; SD, standard deviation.

[a] Values for SBP, total cholesterol, HDL cholesterol, BMI, and smoking status were estimated based on the values from individuals with observed measurements

Supplementary Table 3. The proportion of individuals with and without statin treatment at baseline

Age at baseline	With statin treatment at baseline, n (%)	Without statin treatment at baseline, n (%)	Age at baseline	With statin treatment at baseline, n (%)	Without statin treatment at baseline, n (%)
40	353 (0.8)	43,620 (99.2)	66	2,853 (14.3)	17,076 (85.7)
41	440 (1.0)	43,485 (99.0)	67	2,895 (15.0)	16,374 (85.0)
42	510 (1.2)	42,254 (98.8)	68	2,896 (15.8)	15,418 (84.2)
43	594 (1.4)	40,856 (98.6)	69	2,938 (16.9)	14,487 (83.1)
44	611 (1.5)	39,986 (98.5)	70	2,800 (16.9)	13,799 (83.1)
45	700 (1.8)	38,813 (98.2)	71	2,675 (17.1)	12,970 (82.9)
46	801 (2.1)	37,109 (97.9)	72	2,546 (17.6)	11,957 (82.5)
47	875 (2.3)	36,524 (97.7)	73	2,519 (17.7)	11,722 (82.3)
48	1,038 (2.9)	35,182 (97.1)	74	2,414 (17.5)	11,359 (82.5)
49	1,066 (3.1)	33,529 (96.9)	75	2,236 (16.6)	11,198 (83.4)
50	1,155 (3.5)	31,682 (96.5)	76	1,992 (16.1)	10,390 (83.9)
51	1,221 (3.7)	31,389 (96.3)	77	1,795 (15.4)	9,902 (84.7)
52	1,404 (4.3)	31,231 (95.7)	78	1,503 (14.1)	9,125 (85.9)
53	1,616 (5.1)	30,311 (94.9)	79	1,413 (14.1)	8,641 (86.0)
54	1,689 (5.4)	29,458 (94.6)	80	1,149 (12.2)	8,281 (87.8)
55	1,915 (6.1)	29,445 (93.9)	81	930 (10.6)	7,835 (89.4)
56	2,056 (6.4)	29,995 (93.6)	82	856 (10.1)	7,602 (89.9)
57	2,401 (7.2)	30,853 (92.8)	83	741 (9.5)	7,037 (90.5)
58	2,866 (7.8)	33,906 (92.2)	84	668 (9.0)	6,786 (91.0)
59	2,801 (8.5)	30,259 (91.5)	85	598 (8.2)	6,695 (91.8)
60	2,564 (9.6)	24,030 (90.4)	Total	80,860 (7.2)	1,046,736 (92.8)
61	2,970 (10.4)	25,559 (89.6)			
62	2,830 (11.0)	22,795 (89.0)			
63	2,822 (11.9)	20,970 (88.1)			
64	2,494 (12.3)	17,799 (87.7)			
65	2,651 (13.5)	17,042 (86.5)			

Supplementary Table 4. R^2, D statistic, and Harrell's C statistic of the QRISK2 estimation [a]

	**R^2 (95% CI)**	**D statistic (95% CI)**	**C statistic (95% CI)**
**Men**	32.967 (32.367, 33.568)	1.435 (1.416, 1.455)	0.745 (0.743, 0.748)
**Women**	43.098 (42.492, 43.703)	1.781 (1.759, 1.803)	0.791 (0.789, 0.794)
**Overall**	38.479 (38.054, 38.904)	1.619 (1.604, 1.633)	0.772 (0.770, 0.773)

[a] Calculations for all these statistics were conducted in each of the five imputed datasets and then pooled across imputations using Rubin's rules.

Supplementary Table 5. Comparison of risk factor levels between individuals with predicted risk values in age- and sex-specific above and below the 90th percentile by age group using QRISK2 risk estimations [a]

Risk factor	Predicted risk below the 90th percentile	Predicted risk above the 90th percentile	Standardised difference [b]
**SBP, mean (SD), mmHg**			
40-44	125.3 (14.5)	138.5 (15.9)	-0.9
45-49	127.8 (14.7)	140.6 (16.2)	-0.8
50-54	130.3 (14.8)	142.6 (16.6)	-0.8
55-59	132.9 (15.0)	144.3 (16.8)	-0.7
60-64	135.3 (15.0)	145.2 (17.4)	-0.6
65-69	137.7 (15.3)	146.2 (17.9)	-0.5
70-74	139.9 (15.5)	145.8 (18.2)	-0.4
75-79	141.9 (16.2)	145.0 (17.8)	-0.2
80-85	143.7 (17.1)	144.2 (18.1)	0.0
**Total cholesterol, mean (SD), mmol/L**			
40-44	5.4 (1.0)	5.8 (1.1)	-0.4
45-49	5.4 (1.0)	5.9 (1.1)	-0.4
50-54	5.5 (1.0)	5.9 (1.1)	-0.4
55-59	5.6 (1.1)	6.0 (1.1)	-0.4
60-64	5.6 (1.1)	6.0 (1.1)	-0.4
65-69	5.6 (1.1)	6.0 (1.2)	-0.3
70-74	5.7 (1.1)	6.0 (1.2)	-0.3
75-79	5.7 (1.1)	6.0 (1.2)	-0.3
80-85	5.7 (1.2)	5.9 (1.3)	-0.2
**HDL cholesterol, mean (SD), mmol/L**			
40-44	1.5 (0.4)	1.2 (0.3)	0.7
45-49	1.5 (0.4)	1.2 (0.3)	0.7
50-54	1.5 (0.4)	1.2 (0.3)	0.7
55-59	1.5 (0.4)	1.3 (0.3)	0.8
60-64	1.6 (0.4)	1.3 (0.3)	0.8
65-69	1.6 (0.4)	1.3 (0.3)	0.9
70-74	1.6 (0.4)	1.3 (0.3)	0.9
75-79	1.7 (0.4)	1.3 (0.4)	0.9
80-85	1.7 (0.4)	1.3 (0.4)	0.9
**BMI, mean (SD), kg/m^2**			
40-44	27.2 (5.2)	32.3 (6.2)	-0.9
45-49	27.2 (5.2)	31.8 (6.2)	-0.8
50-54	27.2 (5.2)	31.0 (5.9)	-0.7
55-59	27.2 (5.2)	31.5 (6.1)	-0.6
60-64	26.9 (5.0)	29.7 (5.6)	-0.5
65-69	26.7 (4.9)	28.9 (5.5)	-0.4
70-74	26.5 (4.9)	28.1 (5.3)	-0.3
75-79	25.9 (4.8)	27.1 (5.1)	-0.3
80-85	25.3 (4.7)	26.0 (4.8)	-0.2

Risk factor	Predicted risk below the 90th percentile	Predicted risk above the 90th percentile	Standardised difference [b]
**Current/Ever smoker, n (%)**			
40-44	99,139 (52.4)	18,330 (87.2)	-0.8
45-49	78,230 (48.0)	15,361 (84.8)	-0.8
50-54	58,874 (42.5)	12,608 (81.9)	-0.9
55-59	51,879 (37.3)	11,883 (76.9)	-0.9
60-64	32,408 (32.4)	7,870 (70.8)	-0.8
65-69	20,381 (28.2)	5,075 (63.2)	-0.8
70-74	13,387 (24.1)	3,254 (52.7)	-0.6
75-79	9,692 (21.9)	2,094 (42.6)	-0.5
80-85	6,487 (19.2)	1,149 (30.6)	-0.3

Abbreviations: BMI, body mass index; HDL, high-density lipoprotein; SBP, systolic blood pressure; SD, standard deviation.

[a] Calculations for all these statistics were conducted in each of the five imputed datasets and then pooled across imputations using Rubin's rules.

[b] Standardised differences were calculated between the two groups of individuals with predicted 10-year CVD risk below the 90th percentile and individuals with predicted 10-year CVD risk above the 90th percentile. An absolute standard difference of 0.1 or more indicates that the difference is statistically significant.[1] All the p-values for the comparisons were also <0.001.

Supplementary Table 6. The total number of people stratified as high-risk, overall sensitivity, specificity, and Area Under Receiver Operating Characteristic curve for dichotomised predictions (AUROC-dp) [a] of the stratification strategies across all ages using QRISK2 risk estimations

	Total number of people stratified as high-risk (%)	Sensitivity (%)	Specificity (%)	AUROC-dp (95% CI)
**Men**				
Strategy-A[b]	237,020 (47.5)	80.4	56.8	0.676 (0.674, 0.679)
Strategy-B [c]	246,852 (49.5)	81.7	54.7	0.672 (0.669, 0.674)
**Women**				
Strategy-A	185,427 (33.8)	78.0	70.0	0.724 (0.722, 0.727)
Strategy-B	205,668 (37.5)	80.1	66.2	0.717 (0.715, 0.719)
**Overall**				
Strategy-A	422,447 (40.4)	79.6	63.8	0.704 (0.702, 0.705)
Strategy-B	452,520 (43.2)	81.3	60.8	0.697 (0.696, 0.699)

Abbreviations: AUROC-dp, Area Under Receiver Operating Characteristic curve for dichotomised predictions; CI, confidence intervals

[a] AUROC-dp represents the discriminatory ability to discriminate between those who did and who did not have a CVD event according to the combined risk prediction model and the stratification rule. A higher AUROC-dp value indicates that individuals who experienced a CVD event are more likely to be identified as high-risk under the stratification rules than those who did not have CVD events.

[b] Strategy-A identified high-risk individuals as those with an estimated risk ≥10% (a fixed threshold).

[c] Strategy-B identified high-risk individuals as those with an estimated risk ≥10% or ≥90th-percentile of the age- and sex-specific risk distributions (age- and sex-specific thresholds).

Supplementary Table 7. The sensitivity and specificity of the stratification strategies for individuals aged 40-49, 50-69, and 70-85 using QRISK2 risk estimations

	40-49		50-69		70-85	
	**Sensitivity (%)**	**Specificity (%)**	**Sensitivity (%)**	**Specificity (%)**	**Sensitivity (%)**	**Specificity (%)**
**Men**						
Strategy-A[a]	17.2	94.9	84.7	33.4	100.0	0.0
Strategy-B [b]	25.7	90.2	84.7	33.4	100.0	0.0
**Women**						
Strategy-A	6.0	98.9	59.3	68.1	100.0	0.0
Strategy-B	27.0	90.4	60.7	66.8	100.0	0.0
**Overall**						
Strategy-A	13.7	96.9	75.5	52.0	100.0	0.0
Strategy-B	26.1	90.3	76.0	51.3	100.0	0.0

[a] Strategy-A identified high-risk individuals as those with an estimated 10-year cardiovascular disease risk ≥10% (a fixed threshold).
[b] Strategy-B identified high-risk individuals as those with an estimated 10-year cardiovascular disease risk ≥10% or ≥90th-percentile of the age- and sex-specific risk distributions (age- and sex-specific thresholds).

Supplementary Table 8. The Area Under Receiver Operating Characteristic curve for dichotomised predictions (AUROC-dp) [a] for individuals aged 40-49 and 50-69 using QRISK2 risk estimations

	**40-49**	**50-69**	**70-85**
**Men**			
Strategy-A [b]	0.562 (0.555, 0.568)	0.585 (0.581, 0.588)	0.500 (0.500, 0.500)
Strategy-B [c]	0.579 (0.572, 0.587)	0.585 (0.581, 0.588)	0.500 (0.500, 0.500)
**Women**			
Strategy-A	0.529 (0.524, 0.534)	0.632 (0.628, 0.636)	0.500 (0.500, 0.500)
Strategy-B	0.590 (0.581, 0.600)	0.633 (0.628, 0.637)	0.500 (0.500, 0.500)
**Overall**			
Strategy-A	0.555 (0.550, 0.559)	0.630 (0.627, 0.632)	0.500 (0.500, 0.500)
Strategy-B	0.583 (0.577, 0.589)	0.629 (0.626, 0.631)	0.500 (0.500, 0.500)

Abbreviations: AUROC-dp, Area Under Receiver Operating Characteristic curve for dichotomised predictions; CI, confidence intervals

[a] AUROC-dp represents the discriminatory ability to discriminate between those who did and who did not have a CVD event according to the combined risk prediction model and the stratification rule. A higher AUROC-dp value indicates that individuals who experienced a CVD event are more likely to be identified as high-risk under the stratification rules than those who did not have CVD events.

[b] Strategy-A identified high-risk individuals as those with an estimated 10-year cardiovascular disease risk ≥10% (a fixed threshold).

[c] Strategy-B identified high-risk individuals as those with an estimated 10-year cardiovascular disease risk ≥10% or ≥90th-percentile of the age- and sex-specific risk distributions (age- and sex-specific thresholds).

Supplementary Table 9. The estimated number needed to screen (NNS) and the number needed to treat (NNT) to prevent one new cardiovascular disease (CVD) event using QRISK2 risk estimations in different stratification strategies in the sensitivity analysis by 10-year age group and sex

	**40-49**		**50-69**		**70-85**	
	**NNS**	**NNT**	**NNS**	**NNT**	**NNS**	**NNT**
**Men**						
Strategy-A [a]	263	15	14	10	6	6
Strategy-A1 [b]	218	16	13	10	6	6
Strategy-B [c]	178	19	14	10	6	6
**Women**						
Strategy-A	1,667	21	40	14	8	8
Strategy-A1	1,346	22	37	14	8	8
Strategy-B	398	39	39	14	8	8
**Overall**						
Strategy-A	494	17	20	10	7	7
Strategy-A1	408	19	19	11	7	7
Strategy-B	259	26	20	10	7	7

[a] Strategy-A identified high-risk individuals as those with an estimated risk ≥10% (a fixed threshold).

[b] Strategy-A1, an alternative strategy in the sensitivity analysis, which identified high-risk individuals of CVD as those with estimated risk ≥ **9.2%** (a fixed threshold), to ascertain the same total amount of high-risk individuals as that from strategy-B.

[c] Strategy-B identified high-risk individuals as those with an estimated risk ≥10% or ≥90th-percentile of the age- and sex-specific risk distributions (age- and sex-specific thresholds).

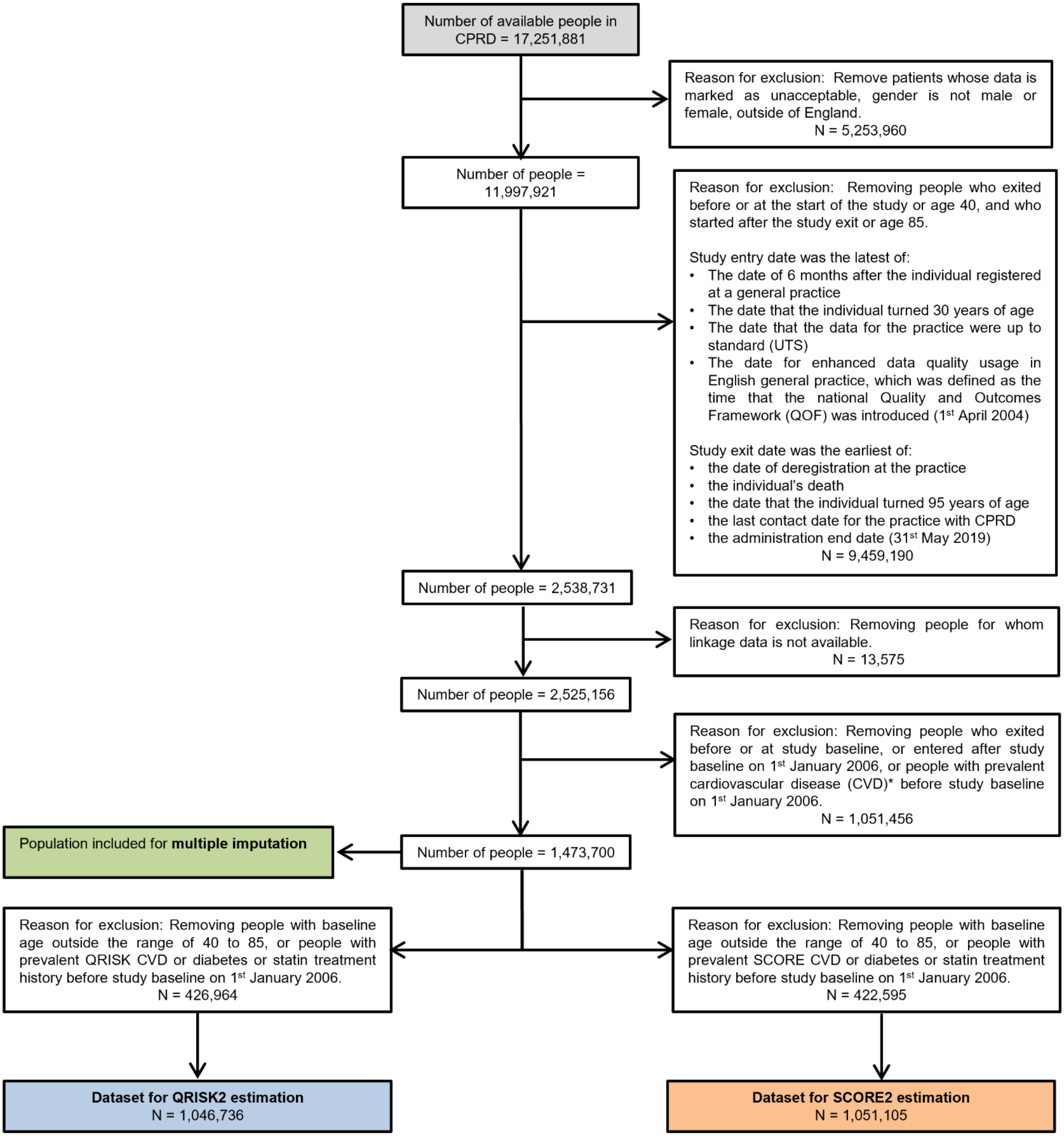

*SCORE2 CVD events were used to exclude prevalent CVD for multiple imputation because there were less SCORE2 CVD events than QRISK CVD events which used a broader CVD definition, thus we can maximise the available sample to preform multiple imputation.

Supplementary Figure 1. Flow chart of study population selection, Clinical Practice Research Datalink, Hospital Episode Statistics, and the Office for National Statistics, England, United Kingdom, 2006-2019

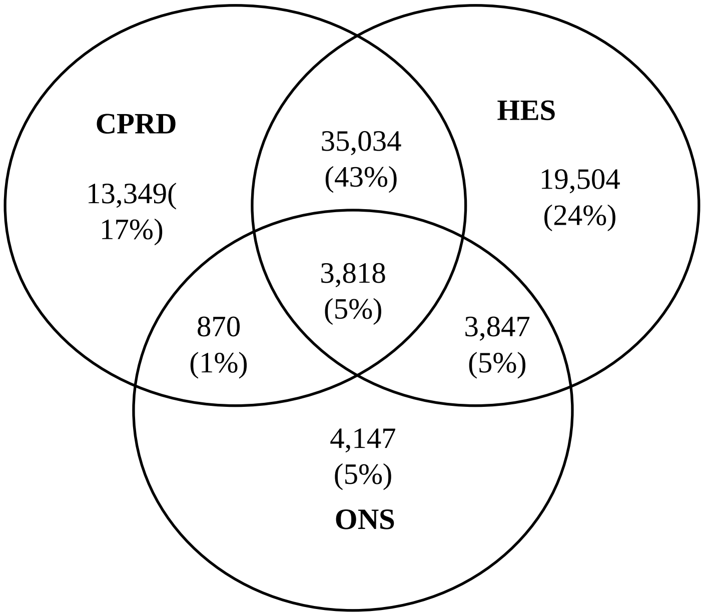

Supplementary Figure 2. Venn diagram of the incident QRISK2 cardiovascular events identified for the QRISK2 risk estimations during follow-up recorded from primary care data in Clinical Practice Research Datalink (CPRD) (n=53,071 first events identified), secondary care data in Hospital Episode Statistics (HES) (n=62,203 first events identified), and mortality records in Office for National Statistics (ONS) (n=12,682 first events identified)

*A total of 80,569 incident QRISK2 CVD events were identified during a median follow-up period of 7.8 (5th, 95th percentile: 0.9, 13.4) years, producing an incident rate of 10.4 (95% CI: 10.3, 10.5) per 1000 person-years.

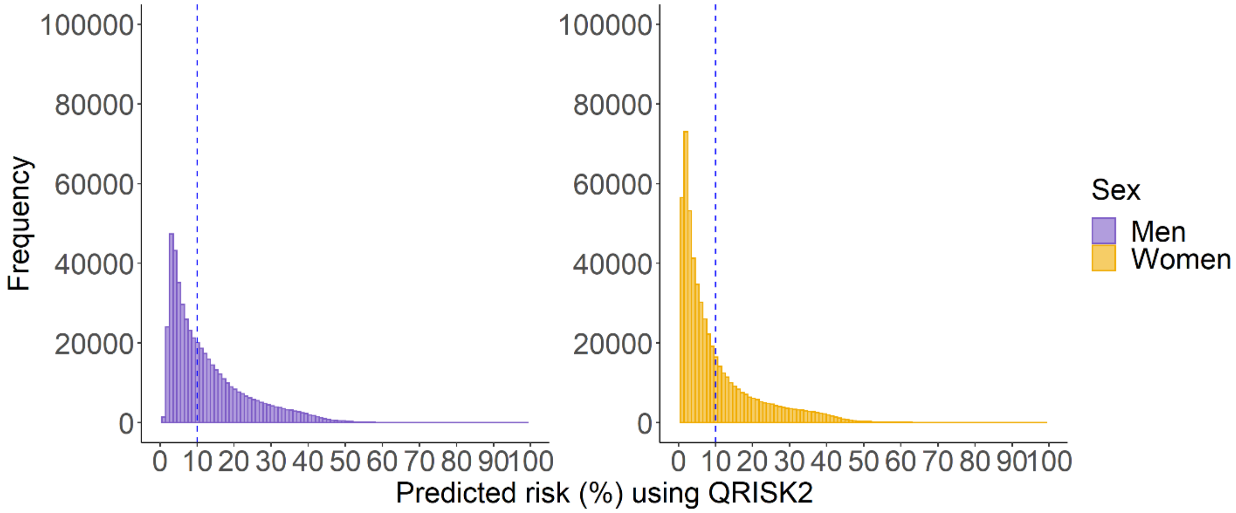

Supplementary Figure 3. Distribution of predicted 10-year cardiovascular disease risk using the QRISK2 algorithms among men and women

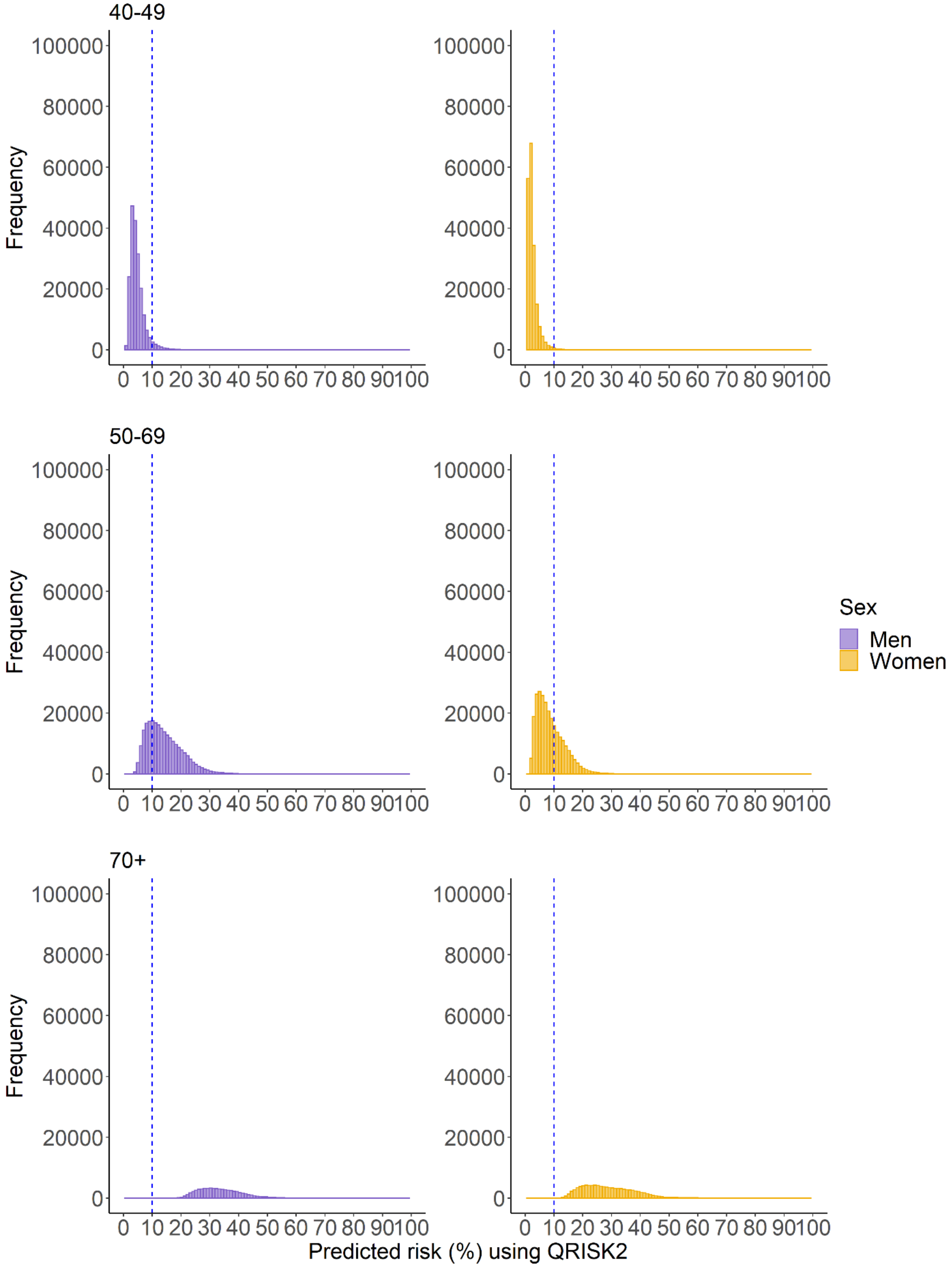

Supplementary Figure 4. Distribution of predicted 10-year cardiovascular disease risk using the QRISK2 algorithms by age group among men and women

*The vertical dashed blue line represents the predicted risk of 10%

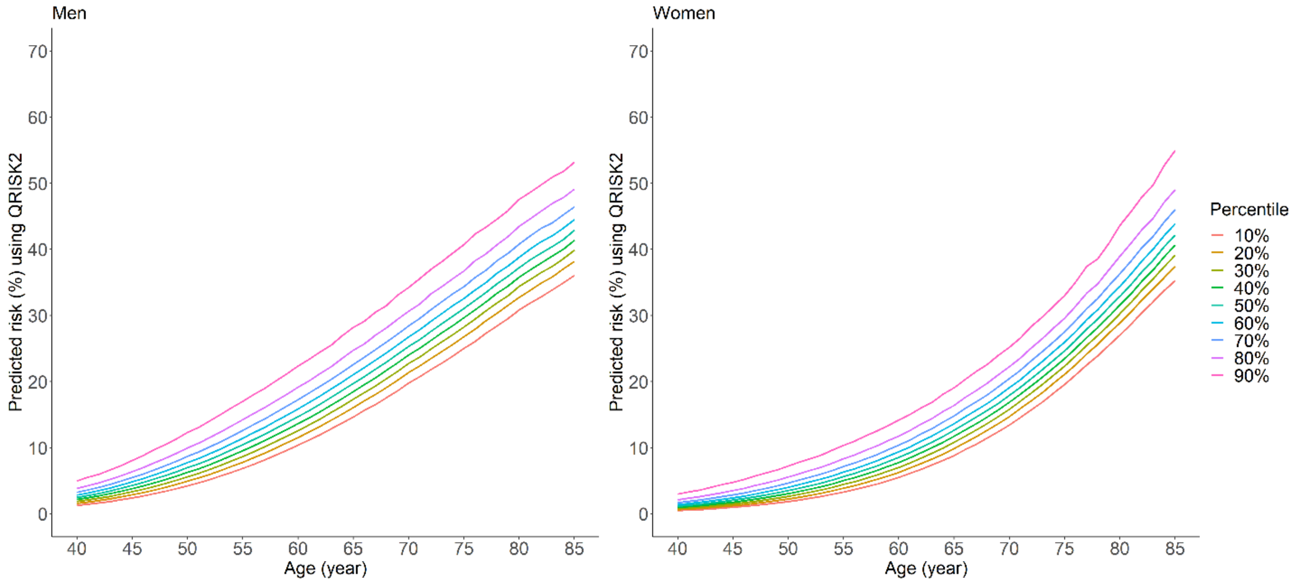

Supplementary Figure 5. Distribution of predicted 10-year cardiovascular disease risk using the QRISK2 algorithms by age in deciles for men and women

*The predicted risk distribution is shown by the 10 values at the tenths of population risk distribution at each age for men and women, separately.

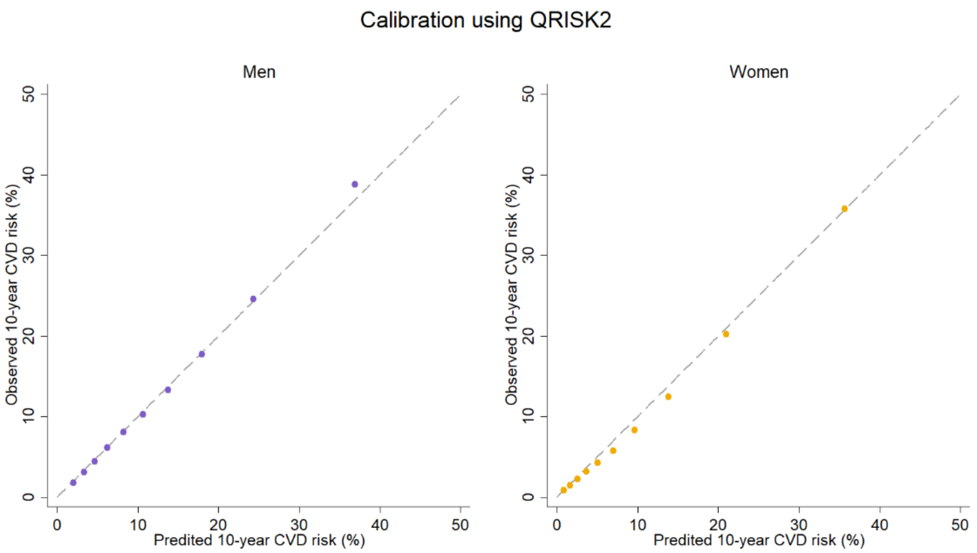

Supplementary Figure 6. Calibration plot of predicted 10-year cardiovascular disease risk versus observed risk using QRISK2 algorithms

*The calibration is presented by comparing the mean predicted 10-year risk vs. the observed 10-year CVD outcomes at each decile of predicted risk for men and women, separately.

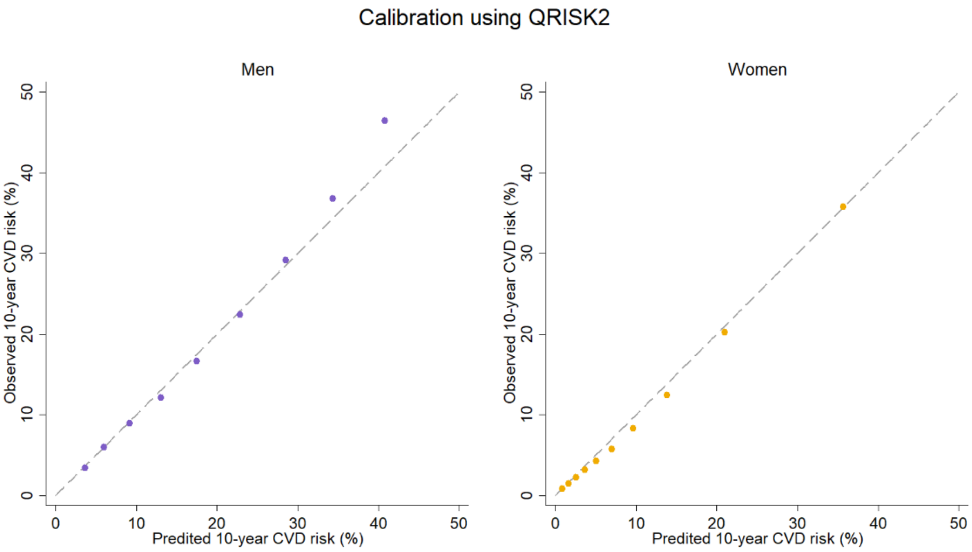

Supplementary Figure 7. Calibration plot of predicted 10-year cardiovascular disease risk versus observed risk by age group using QRISK2 algorithms

*The calibration is presented by comparing the mean predicted 10-year risk vs. the observed 10-year CVD outcomes at each 5-year age group (40-44, 45-49, 50-54, 55-59, 60-64, 65-69, 70-74, 75-79, 80-84) for men and women, separately.

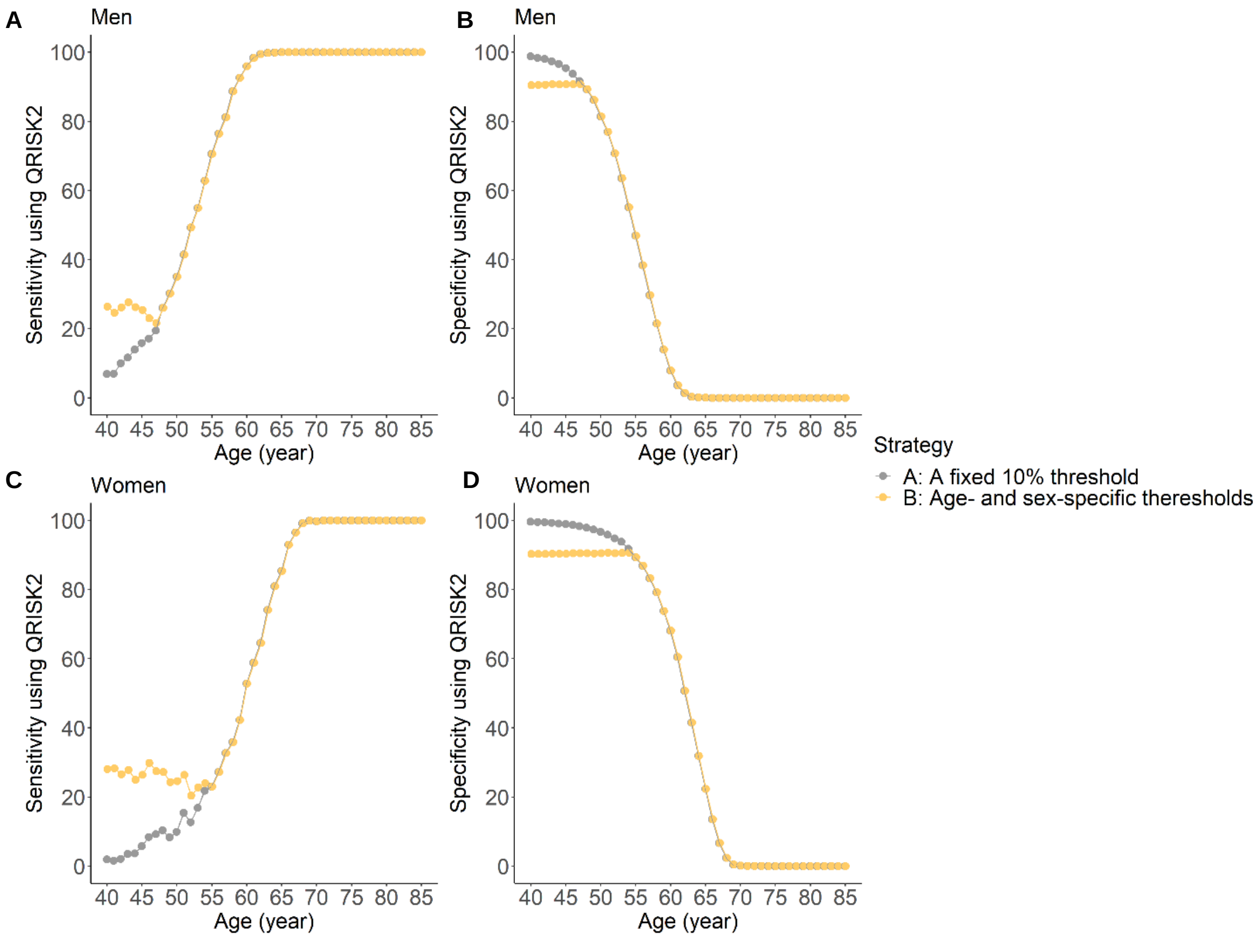

Supplementary Figure 8. Sensitivity (A and C) and specificity (B and D) by age and sex using QRISK2 risk estimations

[a] Strategy-A identified high-risk individuals as those with an estimated 10-year cardiovascular disease risk ≥10% (a fixed threshold).

[b] Strategy-B identified high-risk individuals as those with an estimated 10-year cardiovascular disease risk ≥10% or ≥90th-percentile of the age- and sex-specific risk distributions (age- and sex-specific thresholds).

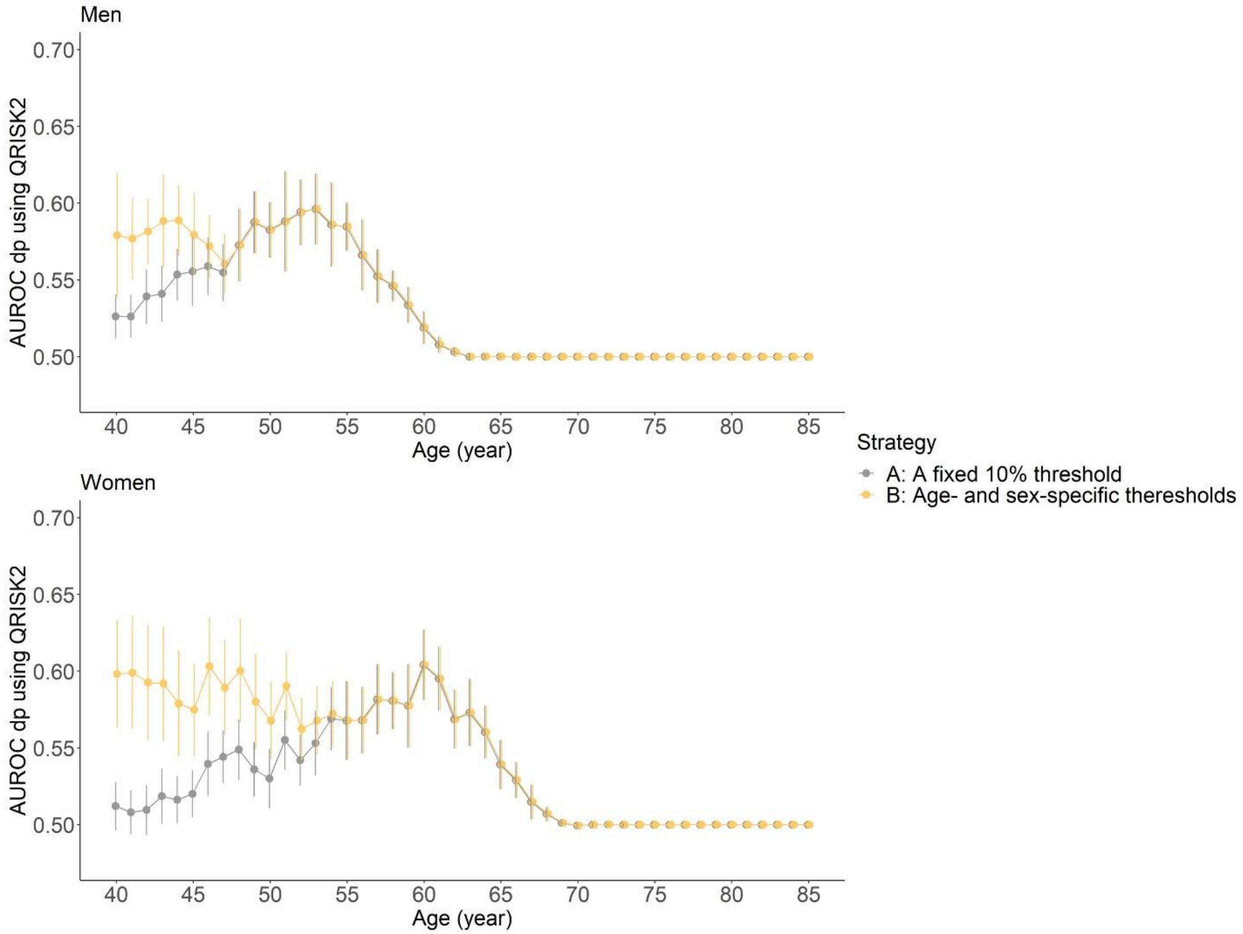

Supplementary Figure 9. Area Under Receiver Operating Characteristic curve for dichotomised predictions (AUROC-dp) in different stratification strategies by age and sex using QRISK2 risk estimations

[a] AUROC-dp represents the discriminatory ability to discriminate between those who did and who did not have a CVD event according to the combined risk prediction model and the stratification rule. A higher AUROC-dp value indicates that individuals who experienced a CVD event are more likely to be identified as high-risk under the stratification rules than those who did not have CVD events.

[b] Strategy-A identified high-risk individuals as those with an estimated 10-year cardiovascular disease risk ≥10% (a fixed threshold).

[c] Strategy-B identified high-risk individuals as those with an estimated 10-year cardiovascular disease risk ≥10% or ≥90th-percentile of the age- and sex-specific risk distributions (age- and sex-specific thresholds).

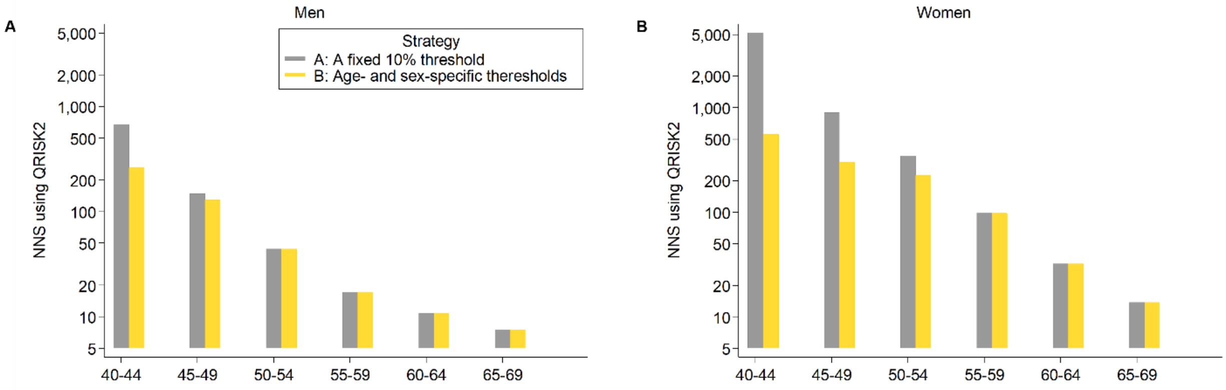

NNS	40-49	50-69
Strategy A	263	14
Strategy B	178	14

NNS	40-49	50-69
Strategy A	1,667	40
Strategy B	398	39

Supplementary Figure 10. The estimated number needed to screen (NNS) to prevent one new CVD event in different stratification strategies by 5-year age group for men (A) and women (B) using QRISK2 risk estimations

[a] Numbers needed to screen to prevent one event are shown on the natural log scale for presentation.
[b] Strategy-A identified high-risk individuals as those with an estimated 10-year cardiovascular disease risk ≥10% (a fixed threshold).
[c] Strategy-B identified high-risk individuals as those with an estimated 10-year cardiovascular disease risk ≥10% or ≥90th-percentile of the age- and sex-specific risk distributions (age- and sex-specific thresholds).

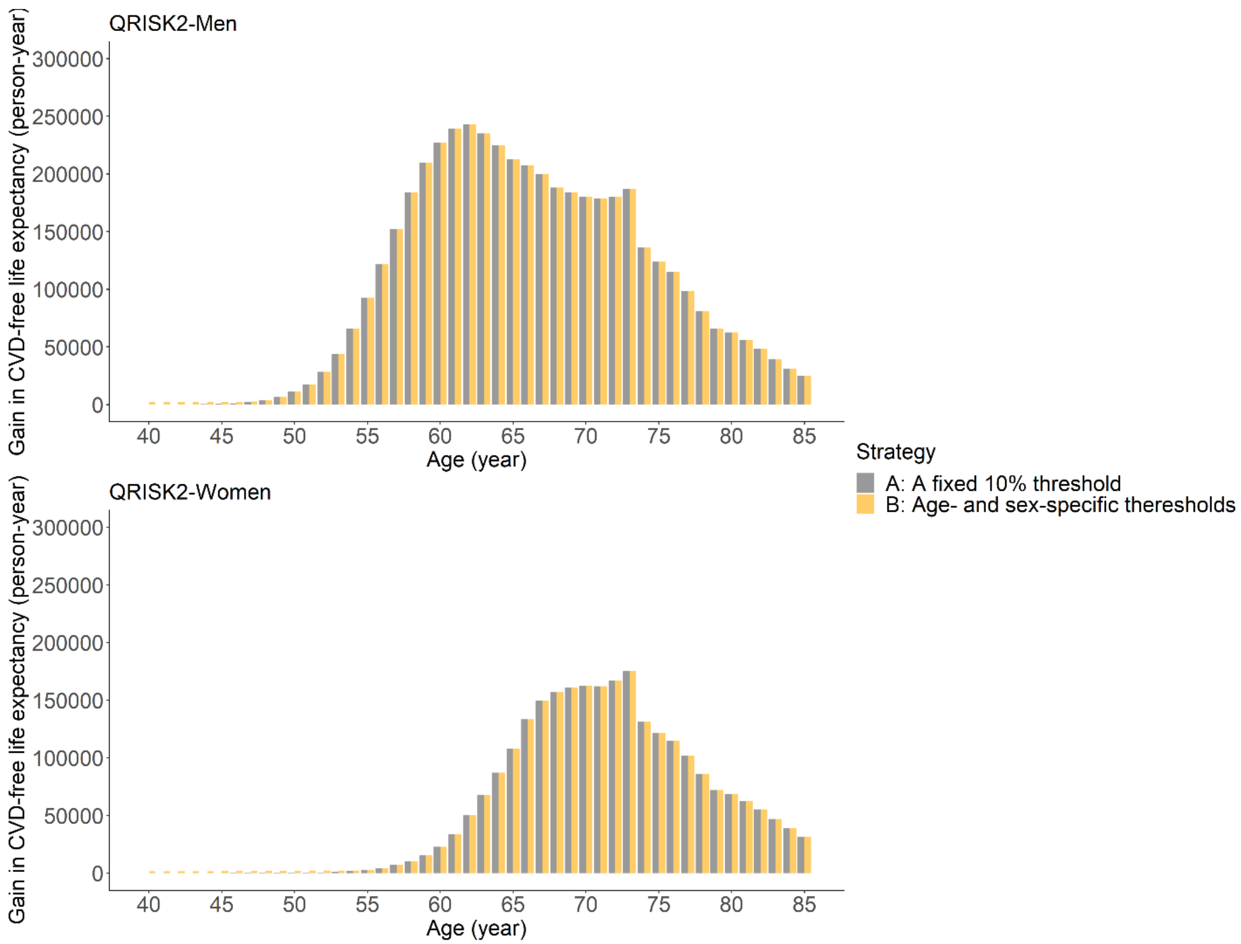

Supplementary Figure 11. Gain in CVD-free life-years (in person-years) from statin treatment given to the high-risk population using different stratification strategies in the standard English population using QRISK2 risk estimations

[a] Strategy-A identified high-risk individuals as those with an estimated 10-year cardiovascular disease risk ≥10% (a fixed threshold).

[b] Strategy-B identified high-risk individuals as those with an estimated 10-year cardiovascular disease risk ≥10% or ≥90th-percentile of the age- and sex-specific risk distributions (age- and sex-specific thresholds).

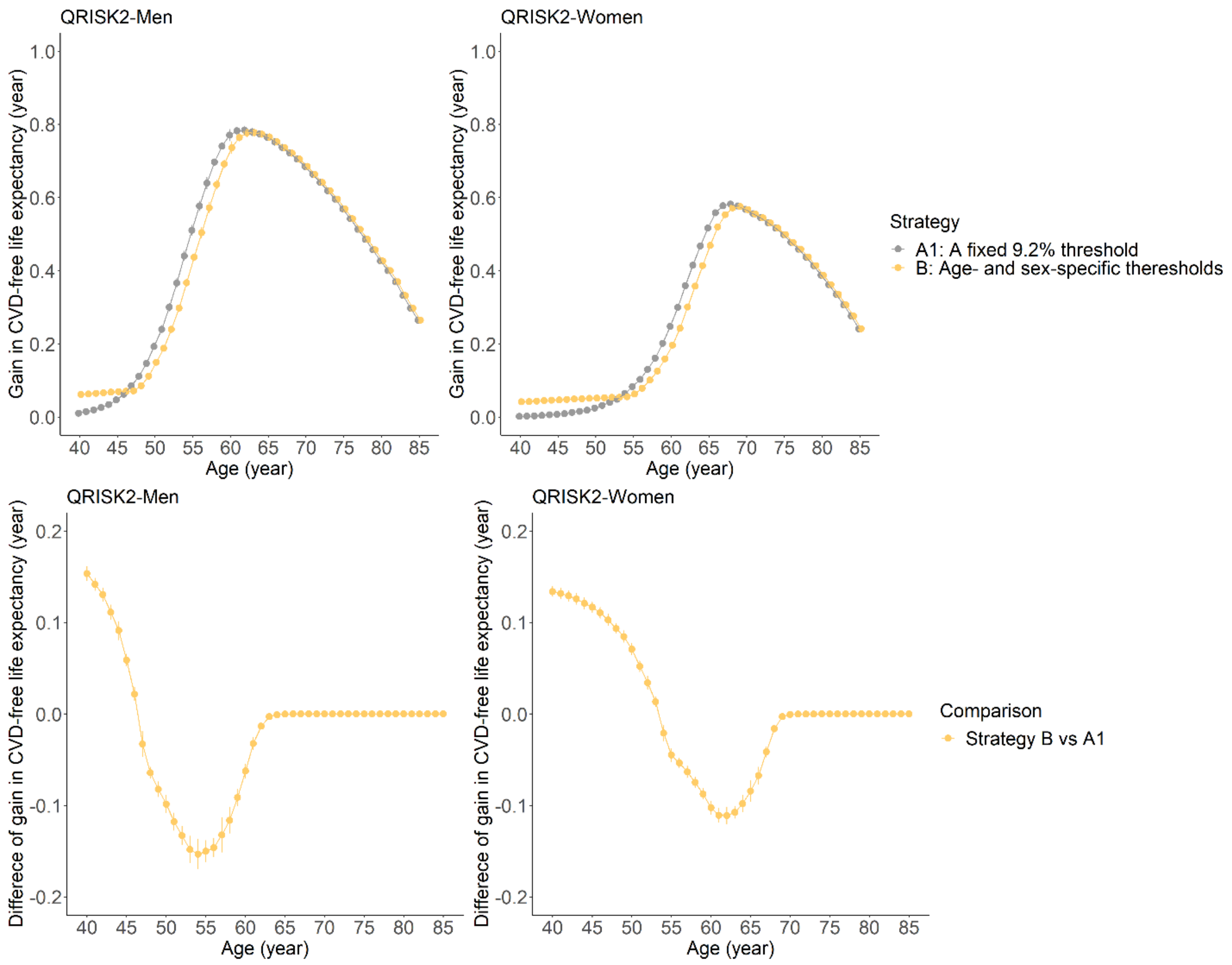

Supplementary Figure 12. Sensitivity analysis - Population average gain in CVD-free life-years from statin treatment given to the high-risk population and difference in the gain comparing different stratification strategies using QRISK2 risk estimations.

[a] Results are shown as the population average gain in CVD-free life-years (A and B) and the difference in the gain comparing strategies-B and -C versus strategy-A (C and D).

[b] Strategy-A1, an alternative strategy in the sensitivity analysis, which identified high-risk individuals of CVD as those with estimated risk ≥ **9.2%** (a fixed threshold), to ascertain the same total amount of high-risk individuals as that from strategy-B.

[c] Strategy-B identified high-risk individuals as those with an estimated 10-year cardiovascular disease risk ≥10% or ≥90th-percentile of the age- and sex-specific risk distributions (age- and sex-specific thresholds).

Results using SCORE2 risk estimations

Characteristics of the study population

The sensitivity analyses using SCORE2 estimations included a total of 1,051,105 eligible individuals (note, the sample sizes were different as different CVD outcomes were defined in QRISK2 and SCORE2 to exclude individuals with prevalent CVD). Characteristics of the study participants at baseline are summarised in **Supplementary Table 10**. Risk factor levels of SBP, total and HDL cholesterol, BMI, and smoking status for both observed and imputed values are provided in **Supplementary Table 11**.

Supplementary Table 10. Baseline characteristics of individuals included for SCORE2 risk estimations [a]

Characteristics [b]	Dataset for SCORE2 estimation (N = 1,051,105)	
	**Men (n = 500,697, 47.6%)**	**Women (n = 550,408, 52.4%)**
Age at baseline, mean (SD), year	55.3 (11.2)	57.1 (12.1)
SBP, mean (SD), mmHg	134.7 (15.5)	131.7 (17.1)
Total cholesterol, mean (SD), mmol/L	5.5 (1.1)	5.6 (1.1)
HDL cholesterol, mean (SD), mmol/L	1.4 (0.4)	1.7 (0.4)
Total/HDL cholesterol ratio, mean (SD)	4.3 (1.3)	3.6 (1.1)
BMI, mean (SD), kg/m^2	27.5 (4.7)	27.1 (5.8)
Current/Ever smoker, n (%)	217,767 (43.5)	232,831 (42.3)
Ethnicity, n (%)		
White/not recorded	492,486 (98.4)	539,723 (98.1)
Indian	1,853 (0.4)	2,563 (0.5)
Pakistani	598 (0.1)	682 (0.1)
Chinese	397 (0.1)	591 (0.1)
Bangladeshi	183 (<0.1)	144 (<0.1)
Other Asian	796 (0.2)	1,159 (0.2)
Black Caribbean	1173 (0.2)	1,665 (0.3)
Black African	892 (0.2)	1,077 (0.2)
Other	2319 (0.5)	2,804 (0.5)
Prescription for antihypertensive medication, n (%)	90,641 (18.1)	158,587 (28.8)
Chronic renal disease, n (%)	738 (0.1)	964 (0.2)
Atrial fibrillation, n (%)	6,992 (1.4)	5,964 (1.1)
Rheumatoid arthritis, n (%)	3,435 (0.7)	9,273 (1.7)
Family history of CHD, n (%)	15,664 (3.1)	21,936 (4.0)

Abbreviations: BMI, body mass index; HDL, high-density lipoprotein; SBP, systolic blood pressure; SD, standard deviation.

[a] Variables of age, sex, smoking status, SBP, total- and HDL-cholesterol, and diabetes status are used in SCORE2 risk estimation.

[b] Values for SBP, total cholesterol, HDL cholesterol, BMI, and smoking status were estimated based on the pooled results from five imputed datasets

Supplementary Table 11. Number of individuals with records for the key risk factors and the comparison for observed values (before imputation) and imputed values of individuals included for SCORE2 estimations

**Characteristics**	**Dataset for SCORE2 estimation (N = 1,051,105)**	
	**Men (n = 500,697, 47.6%)**	**Women (n = 550,408, 52.4%)**
Number of persons with SBP value, n (%)	227,847 (45.5)	341,724 (62.1)
SBP (before imputation), mean (SD), mmHg	136.4 (15.9)	133.1 (17.4)
SBP (after imputation), mean (SD), mmHg	134.7 (15.5)	131.7 (17.1)
Number of persons with total cholesterol value, n (%)	98,971 (19.8)	120,529 (21.9)
Total cholesterol (before imputation), mean (SD), mmol/L	5.5 (1.0)	5.8 (1.0)
Total cholesterol (after imputation), mean (SD), mmol/L	5.5 (1.1)	5.6 (1.1)
Number of persons with HDL cholesterol value, n (%)	75,256 (15.0)	91,315 (16.6)
HDL cholesterol (before imputation), mean (SD), mmol/L	1.4 (0.4)	1.7 (0.4)
HDL cholesterol (after imputation), mean (SD), mmol/L	1.4 (0.4)	1.7 (0.4)
Number of persons with BMI value, n (%)	101,358 (20.2)	164,357 (29.9)
BMI (before imputation), mean (SD), kg/m^2	27.7 (4.9)	27.4 (6.1)
BMI (after imputation), mean (SD), kg/m^2	27.5 (4.7)	27.1 (5.8)
Number of persons with smoking status value, n (%)	117,451 (23.5)	126,902 (23.1)
Current/Ever smoker (before imputation), n (%)	47,886 (40.8)	55,107 (43.4)
Current/Ever smoker (after imputation), n (%)	217,767 (43.5)	232,831 (42.3)

Abbreviations: BMI, body mass index; HDL, high-density lipoprotein; SBP, systolic blood pressure; SD, standard deviation.

There were 57,728 incident SCORE2 CVD events identified during a median follow-up period of 8.1 (5th, 95th percentile: 1.0, 13.4) years (**Supplementary Figure 13**), with an incidence rate of 7.3 (95% CI: 7.2, 7.4) per 1000 person-years.

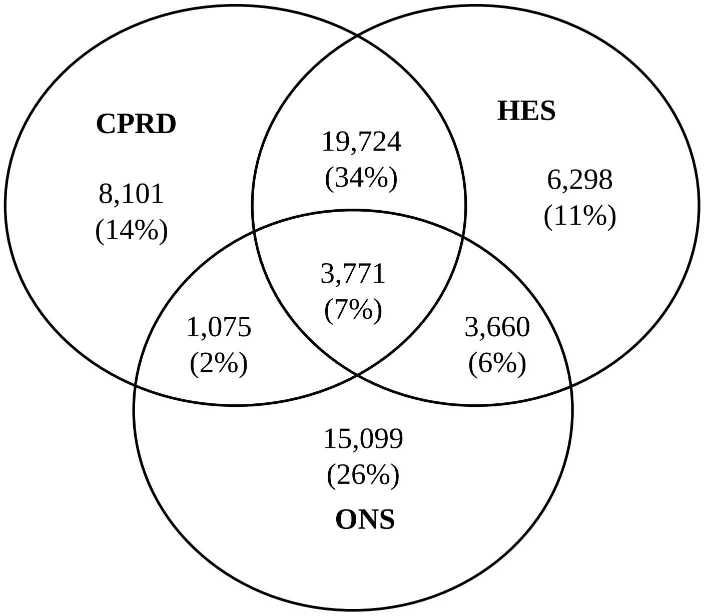

Supplementary Figure 13. Venn diagram of the incident SCORE2 cardiovascular events identified for the SCORE2 risk estimation during follow-up recorded from primary care data in Clinical Practice Research Datalink (CPRD) (n=32,671 first events identified), secondary care data in Hospital Episode Statistics (HES) (n=33,453 first events identified), and mortality records in Office for National Statistics (ONS) (n=23,605 first events identified)

Predicted risk using SCORE2

For SCORE2 and SCORE2-OP results, which accounted for the competing risk of non-CVD death, the mean predicted 10-year CVD risk was 5.9% (**Supplementary Figure 14**). Only 0.03% of younger women and 0.6% of younger men had predicted SCORE2 risk greater than 10%, whereas 39.4% of women and 49.8% of men aged over 60 years had predicted risk higher than 10% (**Supplementary Figures 15** and **16**).

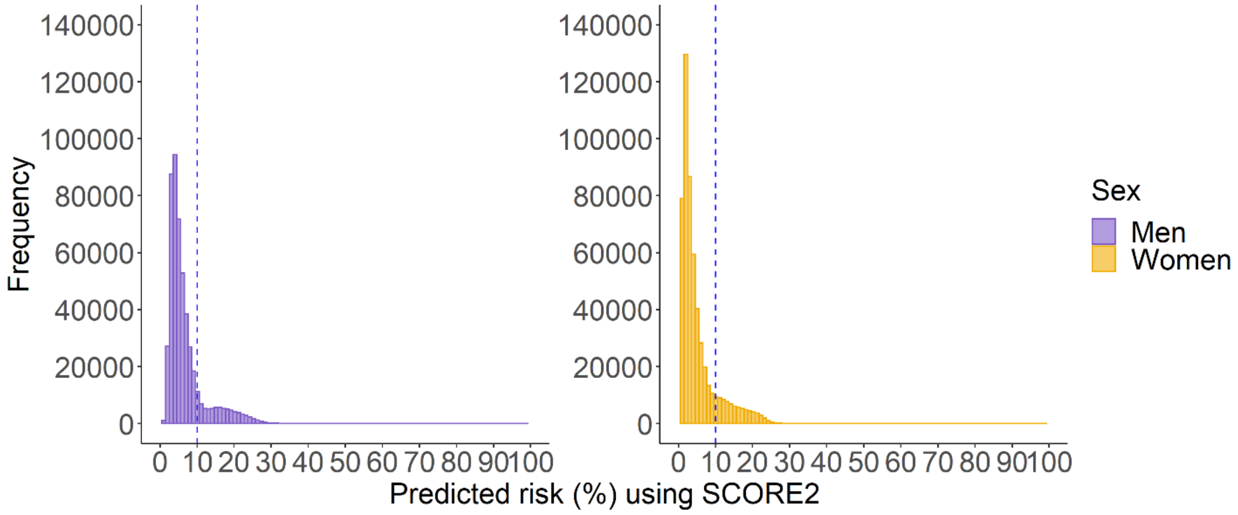

Supplementary Figure 14. Distribution of predicted 10-year cardiovascular disease risk using the SCORE2 algorithms among men and women

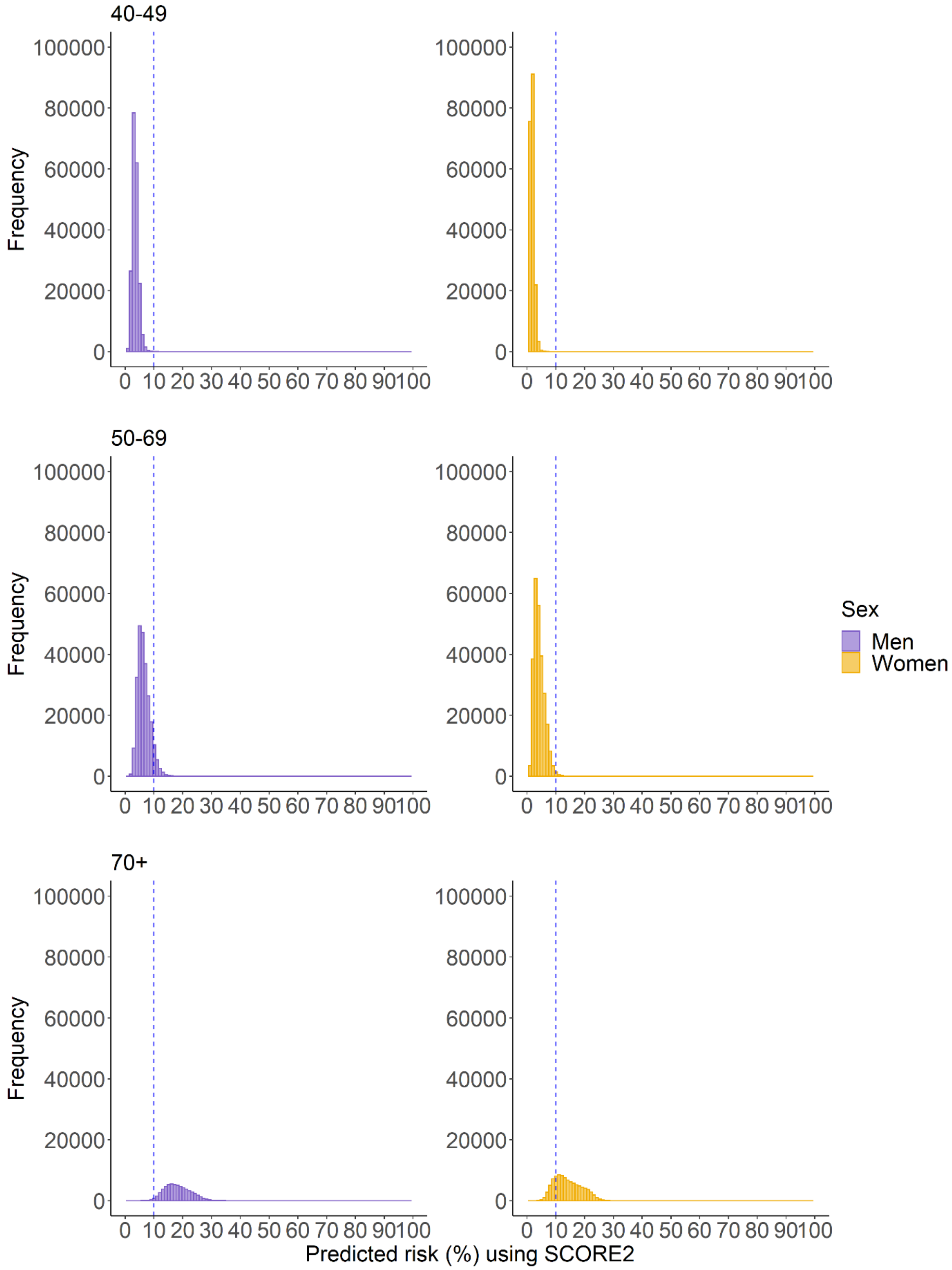

Supplementary Figure 15. Distribution of predicted 10-year cardiovascular disease risk using the SCORE2 algorithms by age group among men and women

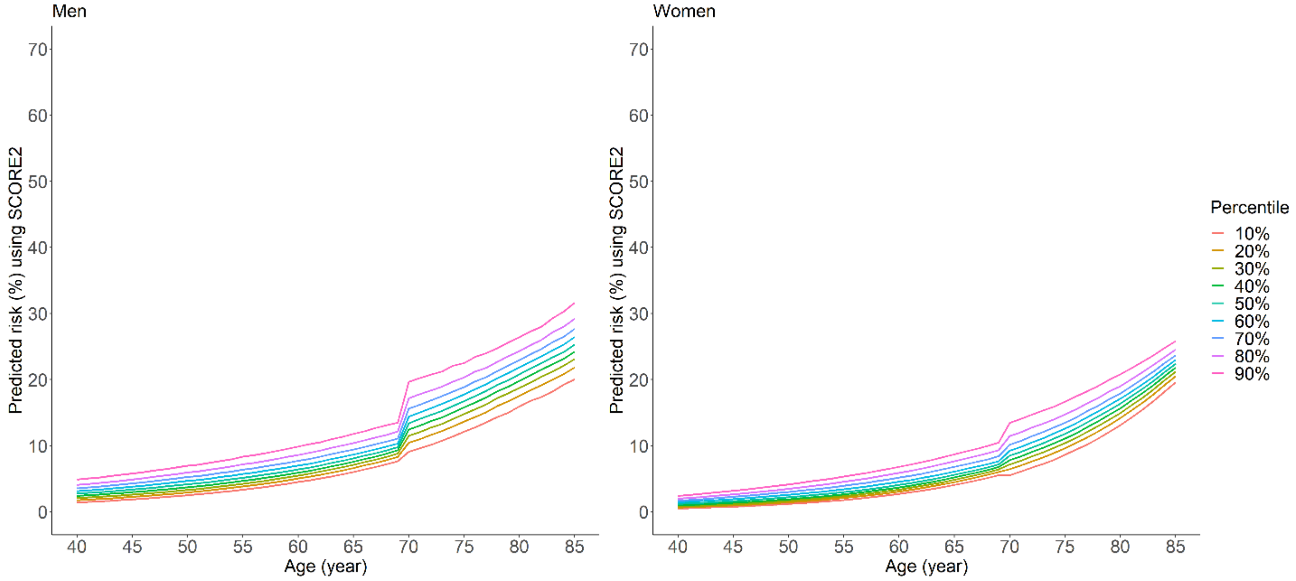

*The vertical dashed blue line represents the predicted risk of 10%

Supplementary Figure 16. Distribution of predicted 10-year cardiovascular disease risk using the SCORE2 algorithms by age in deciles for men and women

*The predicted risk distribution is shown by the 10 values at the tenths of population risk distribution at each age for men and women, separately. The slight discontinuity of the distribution curve reflects the use of two different sets of parameters from SCORE2 algorithms (SCORE2 for people under age 70 and SCORE2-OP for those aged over 70 years).

For SCORE2, the overall R^2 was 45.394 (95% CI: 44.904, 45.883); the C-index was 0.805 (95% CI: 0.803, 0.808) (**Supplementary Table 12**); whereas predicted risks were slightly lower than the observed cumulative incidence due to adjustment for competing risks, an observation consistent with previous findings[2,3] (**Supplementary Figures 17** and **18)**.

Supplementary Table 12. R^2, D statistic, and Harrell's C statistic of the SCORE2 estimation [a]

	**R^2 (95% CI)**	**D statistic (95% CI)**	**C statistic (95% CI)**
**Men**	39.418 (38.709, 40.127)	1.651 (1.626, 1.675)	0.773 (0.769, 0.777)
**Women**	50.802 (50.246, 51.358)	20.80 (2.057, 2.103)	0.832 (0.829, 0.835)
**Overall**	45.394 (44.904, 45.883)	1.866 (1.848, 1.884)	0.805 (0.803, 0.808)

[a] Calculations for all these statistics were conducted in each of the five imputed datasets and then pooled across imputations using Rubin's rules.

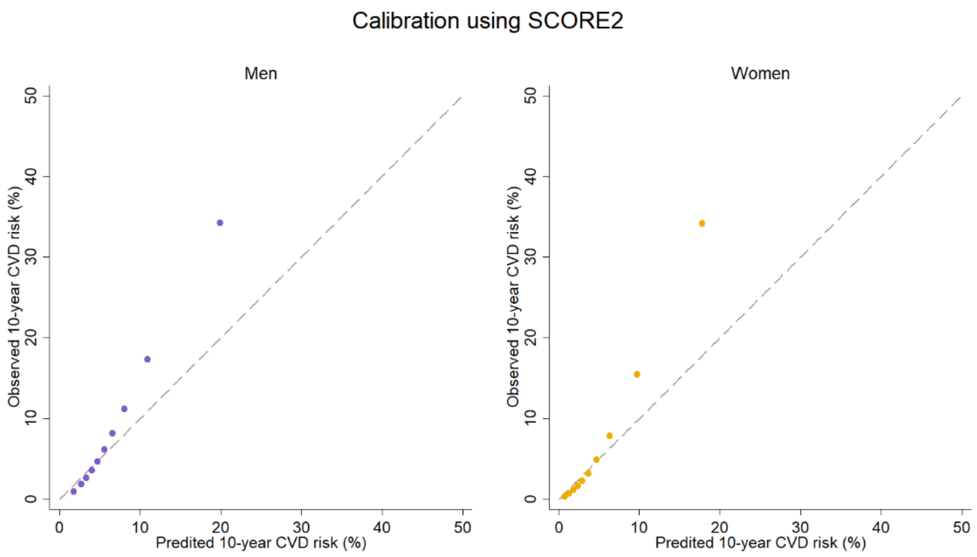

Supplementary Figure 17. Calibration plot of predicted 10-year cardiovascular disease risk versus observed risk using SCORE2 algorithms

*Note: to match the predicted risk which adjusted for competing risks of non-CVD death in SCORE2, observed 10-year CVD risk in SCORE2 was estimated using cumulative incidence function adjusted for competing risks of non-CVD death. The calibration is presented by comparing the mean predicted 10-year risk vs. the observed 10-year CVD outcomes at each decile of predicted risk for men and women, separately. The predicted risks were slightly lower than the observed cumulative incidence due to adjustment for competing risks, an observation consistent with previous findings.[2,3]

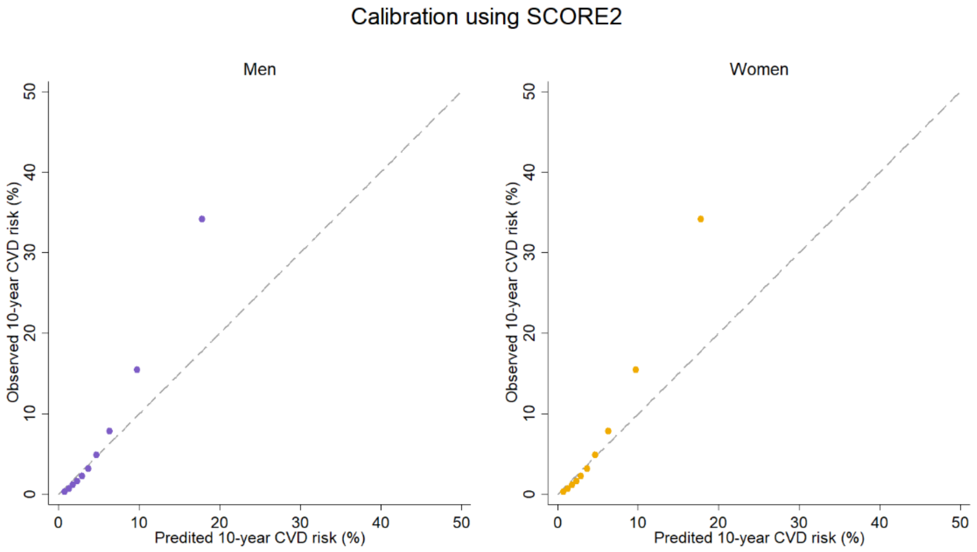

Supplementary Figure 18. Calibration plot of predicted 10-year cardiovascular disease risk versus observed risk by age group using SCORE2 algorithms

*Note: to match the predicted risk which adjusted for competing risks of non-CVD death in SCORE2, observed 10-year CVD risk in SCORE2 was estimated using cumulative incidence function adjusted for competing risks of non-CVD death. The calibration is presented by comparing the mean predicted 10-year risk vs. the observed 10-year CVD

outcomes at each 5-year age group (40-44, 45-49, 50-54, 55-59, 60-64, 65-69, 70-74, 75-79, 80-84) for men and women, separately. The predicted risks were slightly lower than the observed cumulative incidence due to adjustment for competing risks, an observation consistent with previous findings.[2,3]

Thresholds for risk stratification strategies

The number and proportions of people identified as at high-risk of CVD are provided in **Supplementary Table 13**.

Supplementary Table 13. Age- and sex- specific cut-offs of stratifying individuals with high-risk of CVD in each strategy using SCORE2 risk estimations

Age	Men						Women					
	Strategy-A [a]		Strategy-B [b]		Strategy-C [c]		Strategy-A		Strategy-B		Strategy-C	
	Cut-off	Number (%)	Cut-off	Number (%)	Cut-off	Number (%)	Cut-off	Number (%)	Cut-off	Number (%)	Cut-off	Number (%)
40	10%	29 (0.1)	4.9%	2234 (10.0)	7.5%	216 (1.0)	10%	0 (0.0)	2.4%	2128 (10.0)	7.5%	1 (0.0)
41	10%	31 (0.1)	5.0%	2205 (10.0)	7.5%	241 (1.1)	10%	0 (0.0)	2.5%	2144 (10.0)	7.5%	2 (0.0)
42	10%	36 (0.2)	5.2%	2163 (10.0)	7.5%	287 (1.3)	10%	0 (0.0)	2.7%	2064 (10.0)	7.5%	1 (0.0)
43	10%	44 (0.2)	5.4%	2074 (10.0)	7.5%	329 (1.6)	10%	0 (0.0)	2.9%	2013 (10.0)	7.5%	2 (0.0)
44	10%	47 (0.2)	5.6%	2025 (10.0)	7.5%	402 (2.0)	10%	1 (0.0)	3.0%	1976 (10.0)	7.5%	6 (0.0)
45	10%	66 (0.3)	5.8%	1963 (10.0)	7.5%	471 (2.4)	10%	0 (0.0)	3.2%	1919 (10.0)	7.5%	8 (0.0)
46	10%	68 (0.4)	6.0%	1887 (10.0)	7.5%	552 (2.9)	10%	1 (0.0)	3.4%	1826 (10.0)	7.5%	8 (0.0)
47	10%	94 (0.5)	6.2%	1835 (10.0)	7.5%	655 (3.6)	10%	2 (0.0)	3.6%	1818 (10.0)	7.5%	11 (0.1)
48	10%	108 (0.6)	6.4%	1765 (10.0)	7.5%	770 (4.4)	10%	2 (0.0)	3.7%	1755 (10.0)	7.5%	14 (0.1)
49	10%	130 (0.8)	6.7%	1669 (10.0)	7.5%	898 (5.4)	10%	2 (0.0)	3.9%	1687 (10.0)	7.5%	21 (0.1)
50	10%	149 (0.9)	7.0%	1586 (10.0)	10%	149 (0.9)	10%	3 (0.0)	4.1%	1585 (10.0)	10%	3 (0.0)
51	10%	168 (1.1)	7.1%	1549 (10.0)	10%	168 (1.1)	10%	4 (0.0)	4.4%	1593 (10.0)	10%	4 (0.0)
52	10%	218 (1.4)	7.4%	1538 (10.0)	10%	218 (1.4)	10%	5 (0.0)	4.6%	1588 (10.0)	10%	5 (0.0)
53	10%	273 (1.8)	7.7%	1497 (10.0)	10%	273 (1.8)	10%	10 (0.1)	4.8%	1537 (10.0)	10%	10 (0.1)
54	10%	321 (2.2)	8.0%	1441 (10.0)	10%	321 (2.2)	10%	9 (0.1)	5.0%	1509 (10.0)	10%	9 (0.1)
55	10%	435 (3.0)	8.3%	1436 (10.0)	10%	435 (3.0)	10%	10 (0.1)	5.3%	1512 (10.0)	10%	10 (0.1)
56	10%	527 (3.6)	8.6%	1464 (10.0)	10%	527 (3.6)	10%	15 (0.1)	5.6%	1540 (10.0)	10%	15 (0.1)
57	10%	696 (4.6)	8.9%	1501 (10.0)	10%	696 (4.6)	10%	23 (0.1)	5.9%	1588 (10.0)	10%	23 (0.1)
58	10%	942 (5.8)	9.2%	1638 (10.0)	10%	942 (5.8)	10%	40 (0.2)	6.2%	1760 (10.0)	10%	40 (0.2)
59	10%	1061 (7.3)	9.5%	1454 (10.0)	10%	1061 (7.3)	10%	43 (0.3)	6.5%	1579 (10.0)	10%	43 (0.3)
60	10%	1067 (9.2)	9.9%	1159 (10.0)	10%	1067 (9.2)	10%	52 (0.4)	6.8%	1249 (10.0)	10%	52 (0.4)
61	10%	1405 (11.4)	10%	1405 (11.4)	10%	1405 (11.4)	10%	82 (0.6)	7.1%	1328 (10.0)	10%	82 (0.6)
62	10%	1471 (13.7)	10%	1471 (13.7)	10%	1471 (13.7)	10%	107 (0.9)	7.5%	1212 (10.0)	10%	107 (0.9)
63	10%	1706 (17.1)	10%	1706 (17.1)	10%	1706 (17.1)	10%	147 (1.3)	7.8%	1104 (10.0)	10%	147 (1.3)
64	10%	1698 (20.3)	10%	1698 (20.3)	10%	1698 (20.3)	10%	193 (2.0)	8.3%	953 (10.0)	10%	193 (2.0)
65	10%	1887 (23.9)	10%	1887 (23.9)	10%	1887 (23.9)	10%	312 (3.4)	8.7%	922 (10.0)	10%	312 (3.4)
66	10%	2169 (27.9)	10%	2169 (27.9)	10%	2169 (27.9)	10%	437 (4.7)	9.1%	939 (10.0)	10%	437 (4.7)
67	10%	2450 (32.6)	10%	2450 (32.6)	10%	2450 (32.6)	10%	604 (6.8)	9.5%	892 (10.0)	10%	604 (6.8)
68	10%	2685 (38.2)	10%	2685 (38.2)	10%	2685 (38.2)	10%	823 (9.7)	10%	849 (10.0)	10%	823 (9.7)
69	10%	2960 (45.8)	10%	2960 (45.8)	10%	2960 (45.8)	10%	1118 (13.8)	10%	1118 (13.8)	10%	1118 (13.8)

70	10%	4922 (83.7)	10%	4922 (83.7)	15%	2049 (34.9)	10%	2544 (31.7)	10%	2544 (31.7)	15%	420 (5.2)
71	10%	4920 (87.4)	10%	4920 (87.4)	15%	2269 (40.3)	10%	2811 (37.7)	10%	2811 (37.7)	15%	499 (6.7)
72	10%	4623 (90.7)	10%	4623 (90.7)	15%	2363 (46.3)	10%	3213 (46.1)	10%	3213 (46.1)	15%	622 (8.9)
73	10%	4566 (93.5)	10%	4566 (93.5)	15%	2572 (52.7)	10%	3820 (54.9)	10%	3820 (54.9)	15%	787 (11.3)
74	10%	4550 (95.9)	10%	4550 (95.9)	15%	2856 (60.2)	10%	4417 (65.4)	10%	4417 (65.4)	15%	940 (13.9)
75	10%	4442 (97.5)	10%	4442 (97.5)	15%	3093 (67.9)	10%	5114 (75.1)	10%	5114 (75.1)	15%	1247 (18.3)
76	10%	4074 (98.3)	10%	4074 (98.3)	15%	3092 (74.6)	10%	5419 (84.8)	10%	5419 (84.8)	15%	1528 (23.9)
77	10%	3925 (98.9)	10%	3925 (98.9)	15%	3190 (80.4)	10%	5581 (91.3)	10%	5581 (91.3)	15%	1971 (32.2)
78	10%	3544 (99.5)	10%	3544 (99.5)	15%	3087 (86.6)	10%	5482 (95.8)	10%	5482 (95.8)	15%	2412 (42.2)
79	10%	3392 (99.6)	10%	3392 (99.6)	15%	3063 (90.0)	10%	5334 (98.4)	10%	5334 (98.4)	15%	3011 (55.6)
80	10%	3177 (99.8)	10%	3177 (99.8)	15%	2977 (93.6)	10%	5257 (99.4)	10%	5257 (99.4)	15%	3652 (69.1)
81	10%	2916 (99.9)	10%	2916 (99.9)	15%	2801 (96.0)	10%	5142 (99.9)	10%	5142 (99.9)	15%	4292 (83.4)
82	10%	2763 (99.9)	10%	2763 (99.9)	15%	2693 (97.4)	10%	5102 (100.0)	10%	5102 (100.0)	15%	4733 (92.7)
83	10%	2377 (100.0)	10%	2377 (100.0)	15%	2340 (98.4)	10%	4878 (100.0)	10%	4878 (100.0)	15%	4778 (97.9)
84	10%	2301 (100.0)	10%	2301 (100.0)	15%	2280 (99.1)	10%	4711 (100.0)	10%	4711 (100.0)	15%	4692 (99.6)
85	10%	2231 (100.0)	10%	2231 (100.0)	15%	2223 (99.6)	10%	4742 (100.0)	10%	4742 (100.0)	15%	4740 (100.0)

[a] Strategy-A identified high-risk individuals as those with an estimated risk ≥10% (a fixed threshold).
[b] Strategy-B identified high-risk individuals as those with an estimated risk ≥10% or ≥90th-percentile of the age- and sex-specific risk distributions (age- and sex-specific thresholds).
[c] Strategy-C identified high-risk individuals as those with an estimated risk ≥7.5%, ≥10%, or ≥15% for individuals in younger (40-49), middle-aged (50-69), and older (70 and over) age groups (age-specific thresholds).
Numbers in green: cut-offs<7.5%; numbers in yellow: cut-offs ≥7.5% and <10%; numbers in orange: cut-offs ≥10% and <15%; numbers in red: cut-offs ≥15%.

Under strategy-B, individuals with predicted risk above versus below the 90th percentile, had higher levels of SBP, total cholesterol, BMI, and were more likely to be smokers (**Supplementary Table 14**).

Supplementary Table 14. Comparison of risk factor levels between individuals with predicted risk values in age- and sex-specific above and below the 90th percentile by age group using SCORE2 risk estimation

Risk factor	Predicted risk below the 90th percentile	Predicted risk above the 90th percentile	Standardised difference [b]
**SBP, mean (SD), mmHg**			
40-44	124.9 (14.3)	142.5 (14.0)	-1.2
45-49	127.3 (14.4)	145.1 (14.0)	-1.3
50-54	129.8 (14.5)	147.7 (14.2)	-1.2
55-59	132.2 (14.7)	150.1 (14.5)	-1.2
60-64	134.5 (14.6)	152.4 (15.0)	-1.2
65-69	136.7 (14.6)	155.2 (15.9)	-1.2
70-74	139.0 (14.9)	153.7 (17.8)	-0.9
75-79	140.6 (15.4)	156.2 (18.6)	-0.9
80-85	141.9 (16.0)	159.5 (20.2)	-0.9
**Total cholesterol, mean (SD), mmol/L**			
40-44	5.3 (1.0)	6.2 (1.2)	-0.8
45-49	5.4 (1.0)	6.2 (1.2)	-0.8
50-54	5.5 (1.0)	6.2 (1.1)	-0.7
55-59	5.5 (1.0)	6.2 (1.1)	-0.6
60-64	5.6 (1.1)	6.1 (1.1)	-0.5
65-69	5.6 (1.1)	6.0 (1.2)	-0.4
70-74	5.7 (1.1)	5.8 (1.1)	-0.1
75-79	5.7 (1.1)	6.0 (1.2)	-0.3
80-85	5.6 (1.2)	6.3 (1.2)	-0.6
**HDL cholesterol, mean (SD), mmol/L**			
40-44	1.5 (0.4)	1.2 (0.3)	0.9
45-49	1.5 (0.4)	1.2 (0.3)	0.9
50-54	1.5 (0.4)	1.2 (0.3)	0.9
55-59	1.5 (0.4)	1.3 (0.3)	0.8
60-64	1.6 (0.4)	1.3 (0.3)	0.8
65-69	1.6 (0.4)	1.3 (0.3)	0.7
70-74	1.6 (0.4)	1.2 (0.3)	1.2
75-79	1.7 (0.4)	1.3 (0.3)	1.1
80-85	1.7 (0.4)	1.3 (0.3)	1.0
**BMI, mean (SD), kg/m^2**			
40-44	27.4 (5.4)	30.5 (5.8)	-0.6
45-49	27.4 (5.4)	30.0 (5.7)	-0.5
50-54	27.4 (5.3)	29.5 (5.5)	-0.4
55-59	27.3 (5.2)	28.7 (5.3)	-0.3
60-64	27.1 (5.1)	28.0 (5.2)	-0.2
65-69	26.9 (5.0)	27.3 (5.0)	-0.1
70-74	26.6 (5.0)	27.1 (5.0)	-0.1

Risk factor	Predicted risk below the 90th percentile	Predicted risk above the 90th percentile	Standardised difference [b]
75-79	26.0 (4.9)	26.4 (4.9)	-0.1
80-85	25.3 (4.7)	25.6 (4.8)	-0.1
**Current/Ever smoker, n (%)**			
40-44	96,709 (51.1)	20,827 (99.1)	-1.3
45-49	75,745 (46.4)	17,910 (98.8)	-1.4
50-54	56,413 (40.6)	15,181 (98.4)	-1.6
55-59	48,790 (35.0)	15,111 (97.7)	-1.8
60-64	29,654 (29.5)	10,777 (96.7)	-1.9
65-69	17,962 (24.7)	7,628 (94.4)	-2.0
70-74	11,369 (20.2)	5,430 (87.1)	-1.8
75-79	8,037 (17.8)	3,940 (78.7)	-1.5
80-85	5,410 (15.5)	2,413 (62.5)	-1.1

Abbreviations: BMI, body mass index; HDL, high-density lipoprotein; SBP, systolic blood pressure; SD, standard deviation.

[a] Calculations for all these statistics were conducted in each of the five imputed datasets and then pooled across imputations using Rubin's rules.

[b] Standardised differences were calculated between the two groups of individuals with predicted 10-year CVD risk below the 90th percentile and individuals with predicted 10-year CVD risk above the 90th percentile. An absolute standard difference of 0.1 or more indicates that the difference is statistically significant.[1] All the p-values for the comparisons were also <0.001.

Sensitivity, specificity, AUROC-dp, and net benefit

The overall sensitivity and specificity were 57.7% and 87.6% for strategy-A, 61.5% and 80.8% for strategy-B, 44.4% and 91.2% for strategy-C (**Supplementary Table 15**). For younger aged individuals, strategy-B produced higher sensitivity, for example, sensitivity was markedly improved from 3.1% to 19.9%, but with only modest reductions in specificity from 99.1% to 90.5% in men at age 50 using SCORE2 (**Supplementary Table 16** and **Supplementary Figure 19**).

Supplementary Table 15. The total number of people stratified as high-risk, overall sensitivity, specificity, and Area Under Receiver Operating Characteristic curve for dichotomised predictions (AUROC-dp) [a] of the stratification strategies across all ages using SCORE2 risk estimations

	Total number of people stratified as high-risk (%)	Sensitivity (%)	Specificity (%)	AUROC-dp (95% CI)
**Men**				
Strategy-A [b]	83,662 (16.7)	53.2	86.4	0.690 (0.689, 0.693)
Strategy-B [c]	113,237 (22.6)	57.6	80.4	0.686 (0.682, 0.690)
Strategy-C [d]	72,054 (14.4)	45.6	88.2	0.666 (0.662, 0.670)
**Women**				
Strategy-A	77,614 (14.1)	62.7	88.8	0.732 (0.728, 0.735)
Strategy-B	119,255 (21.7)	65.8	81.1	0.722 (0.718, 0.726)
Strategy-C	44,437 (8.1)	43.1	93.9	0.669 (0.665, 0.673)
**Overall**				
Strategy-A	161,276 (15.3)	57.7	87.6	0.705 (0.702, 0.708)
Strategy-B	232,492 (22.1)	61.5	80.8	0.698 (0.695, 0.701)
Strategy-C	116,490 (11.1)	44.4	91.2	0.661 (0.660, 0.664)

Abbreviations: AUROC-dp, Area Under Receiver Operating Characteristic curve for dichotomised predictions; CI, confidence intervals

[a] AUROC-dp represents the discriminatory ability to discriminate between those who did and who did not have a CVD event according to the combined risk prediction model and the stratification rule. A higher AUROC-dp value indicates that individuals who experienced a CVD event are more likely to be identified as high-risk under the stratification rules than those who did not have CVD events.

[b] Strategy-A identified high-risk individuals as those with an estimated risk ≥10% (a fixed threshold).

[c] Strategy-B identified high-risk individuals as those with an estimated risk ≥10% or ≥90th-percentile of the age- and sex-specific risk distributions (age- and sex-specific thresholds).

[d] Strategy-C identified high-risk individuals as those with an estimated risk ≥7.5%, ≥10%, or ≥15% for individuals in younger (40-49), middle-aged (50-69), and older (70 and over) age groups (age-specific thresholds).

Supplementary Table 16. The sensitivity and specificity of the stratification strategies for individuals aged 40-49, 50-69, and 70-85 using SCORE2 risk estimations

	40-49		50-69		70-85	
	**Sensitivity (%)**	**Specificity (%)**	**Sensitivity (%)**	**Specificity (%)**	**Sensitivity (%)**	**Specificity (%)**
**Men**						
Strategy-A[a]	1.5	99.7	21.6	90.9	97.3	5.6
Strategy-B [b]	23.9	90.4	26.9	86.7	97.3	5.6
Strategy-C [c]	7.8	97.7	21.6	90.9	79.4	34.3
**Women**						
Strategy-A	0.3	99.9	5.6	98.6	89.1	27.6
Strategy-B	27.1	90.2	19.3	90.3	89.1	27.6
Strategy-C	0.5	99.9	5.6	98.6	61.0	63.7
**Overall**						
Strategy-A	1.2	99.9	15.8	95.0	92.9	19.4
Strategy-B	24.8	90.3	24.1	88.6	92.9	19.4
Strategy-C	5.8	98.8	15.8	95.0	69.1	52.7

[a] Strategy-A identified high-risk individuals as those with an estimated risk ≥10% (a fixed threshold).

[b] Strategy-B identified high-risk individuals as those with an estimated risk ≥10% or ≥90th-percentile of the age- and sex-specific risk distributions (age- and sex-specific thresholds).

[c] Strategy-C identified high-risk individuals as those with an estimated risk ≥7.5%, ≥10%, or ≥15% for individuals in younger (40-49), middle-aged (50-69), and older (70 and over) age groups (age-specific thresholds).

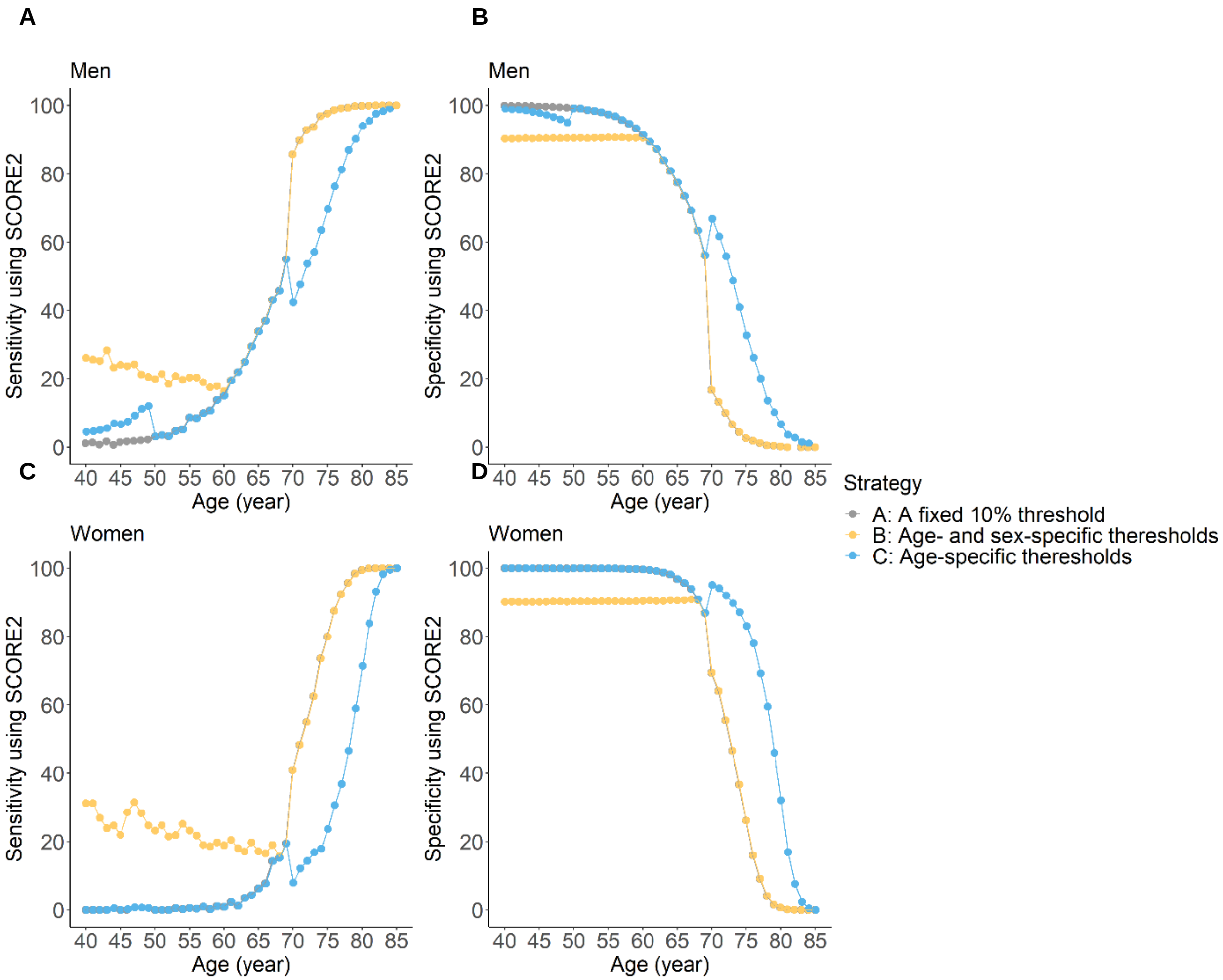

Supplementary Figure 19. Sensitivity (A and C) and specificity (B and D) by age and sex using SCORE2 risk estimations

[a] Strategy-A identified high-risk individuals as those with an estimated 10-year cardiovascular disease risk ≥10% (a fixed threshold).
[b] Strategy-B identified high-risk individuals as those with an estimated 10-year cardiovascular disease risk ≥10% or ≥90th-percentile of the age- and sex-specific risk distributions (age- and sex-specific thresholds).
[c] Strategy-C identified high-risk individuals as those with an estimated 10-year cardiovascular disease risk ≥7.5%, ≥10%, or ≥15% for individuals in younger (40-49), middle-aged (50-69), and older (70 and over) age groups (age-specific thresholds).

The overall AUROC-dp for SCORE2 in combination with the stratification rule was around 0.7 for strategies -A, -B, and -C (**Supplementary Table 15**). However, amongst younger individuals, in contrast with strategy-A, using age- and sex-specific risk thresholds in strategy-B led to significant improvements in AUROC-dp (**Supplementary Figure 20** and **Supplementary Table 17**).

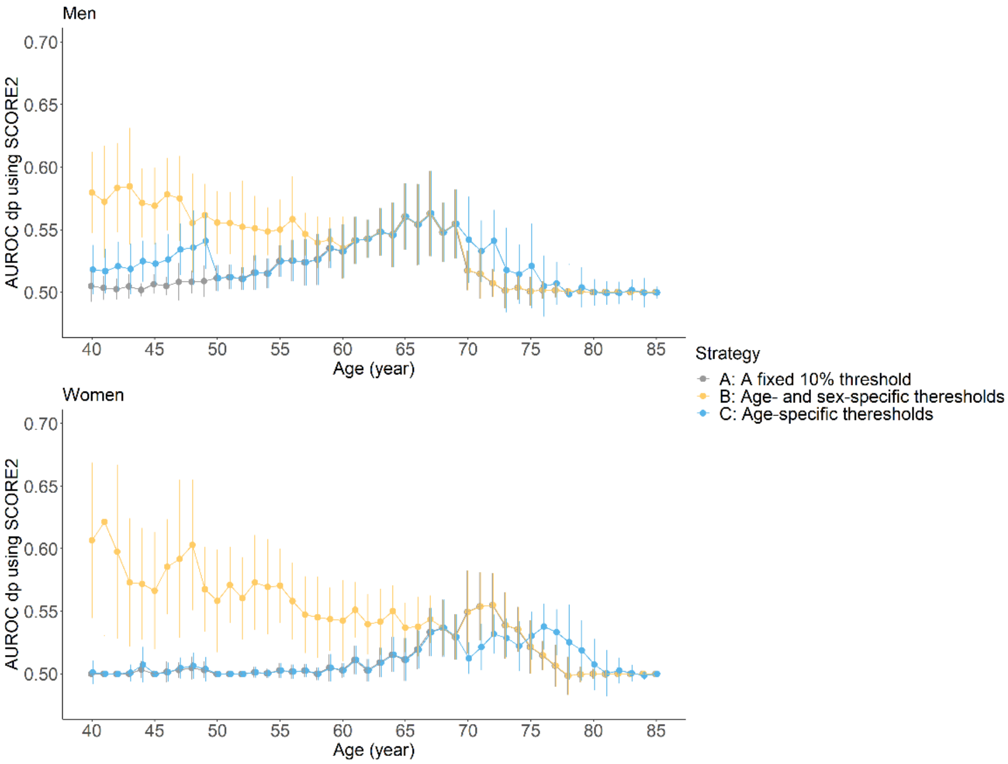

Supplementary Figure 20. Area Under Receiver Operating Characteristic curve for dichotomised predictions (AUROC-dp) in different stratification strategies by age and sex using SCORE2 risk estimations

[a]AUROC-dp represents the discriminatory ability to discriminate between those who did and who did not have a CVD event according to the combined risk prediction model and the stratification rule. A higher AUROC-dp value indicates that individuals who experienced a CVD event are more likely to be identified as high-risk under the stratification rules than those who did not have CVD events.
[b] Strategy-A identified high-risk individuals as those with an estimated 10-year cardiovascular disease risk ≥10% (a fixed threshold).
[c] Strategy-B identified high-risk individuals as those with an estimated 10-year cardiovascular disease risk ≥10% or ≥90th-percentile of the age- and sex-specific risk distributions (age- and sex-specific thresholds).
[d] Strategy-C identified high-risk individuals as those with an estimated 10-year cardiovascular disease risk ≥7.5%, ≥10%, or ≥15% for individuals in younger (40-49), middle-aged (50-69), and older (70 and over) age groups (age-specific thresholds).

Supplementary Table 17. The Area Under Receiver Operating Characteristic curve for dichotomised predictions (AUROC-dp)[a] by age group using SCORE2 risk estimations

	40-49	50-69	70-85
**Men**			
Strategy-A [b]	0.506 (0.503, 0.509)	0.560 (0.556, 0.564)	0.512 (0.508, 0.516)
Strategy-B [c]	0.572 (0.554, 0.589)	0.566 (0.561, 0.571)	0.512 (0.508, 0.516)
Strategy-C [d]	0.529 (0.522, 0.537)	0.560 (0.556, 0.564)	0.555 (0.548, 0.562)
**Women**			
Strategy-A	0.502 (0.500, 0.504)	0.520 (0.517, 0.524)	0.569 (0.565, 0.573)
Strategy-B	0.587 (0.571, 0.603)	0.547 (0.542, 0.552)	0.569 (0.565, 0.573)
Strategy-C	0.503 (0.500, 0.505)	0.520 (0.517, 0.524)	0.603 (0.598, 0.608)
**Overall**			
Strategy-A	0.505 (0.503, 0.507)	0.552 (0.549, 0.555)	0.550 (0.547, 0.553)
Strategy-B	0.576 (0.566, 0.587)	0.562 (0.558, 0.567)	0.550 (0.547, 0.553)
Strategy-C	0.524 (0.518, 0.530)	0.552 (0.549, 0.555)	0.591 (0.587, 0.594)

Abbreviations: AUROC-dp, Area Under Receiver Operating Characteristic curve for dichotomised predictions; CI, confidence intervals

[a] AUROC-dp represents the discriminatory ability to discriminate between those who did and who did not have a CVD event according to the combined risk prediction model and the stratification rule. A higher AUROC-dp value indicates that individuals who experienced a CVD event are more likely to be identified as high-risk under the stratification rules than those who did not have CVD events.

[b] Strategy-A identified high-risk individuals as those with an estimated risk ≥10% (a fixed threshold).

[c] Strategy-B identified high-risk individuals as those with an estimated risk ≥10% or ≥90th-percentile of the age- and sex-specific risk distributions (age- and sex-specific thresholds).

[d] Strategy-C identified high-risk individuals as those with an estimated risk ≥7.5%, ≥10%, or ≥15% for individuals in younger (40-49), middle-aged (50-69), and older (70 and over) age groups (age-specific thresholds).

For men aged 40-60 years and for women aged 40-68 years, the net benefit was higher for strategy-B compared with strategy-A (**Supplementary Figure 21**). For example, the net benefit was 0.18 for strategy-B versus 0.01 for strategy-A for women at age 60 (**Supplementary Figure 21**). Strategy-C also had higher net benefit values for men aged years compared with strategy-A.

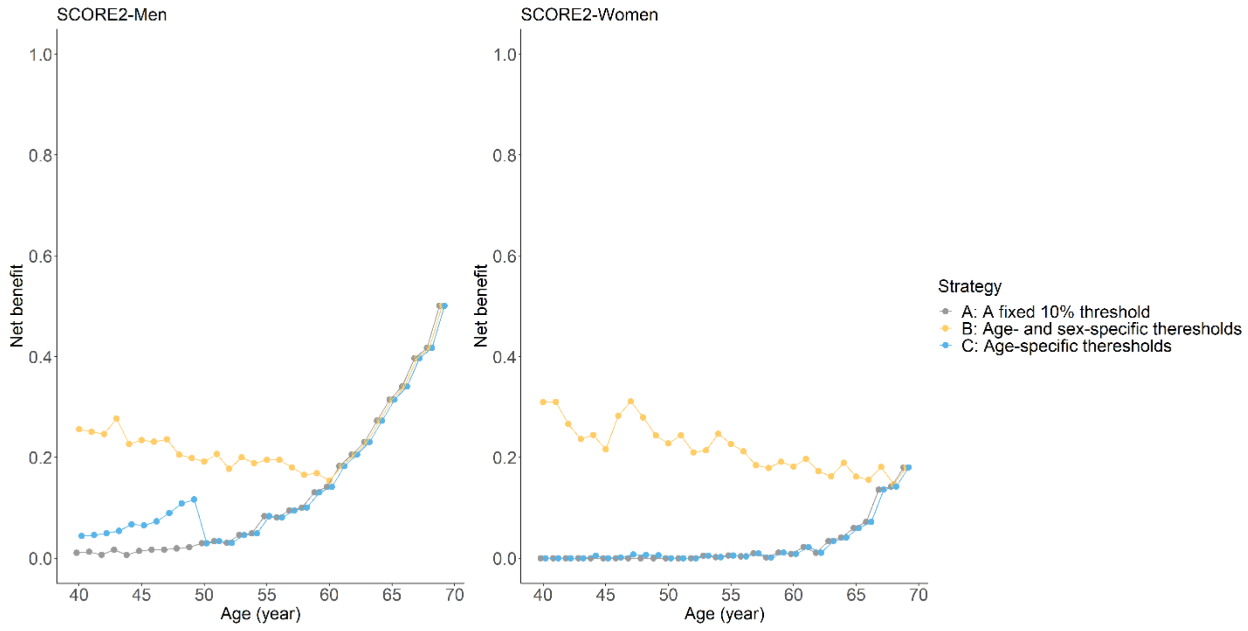

Supplementary Figure 21. Net benefit for different stratification strategies by age for men (A) and women (B) using SCORE2 risk estimations

Estimated numbers needed to screen (NNS) and treat (NNT) to prevent one CVD event

For SCORE2, NNS reductions under strategy-B vs -A were observed amongst people up to age 65 years, with an increase in NNT for people until around age 55-60 years (**Supplementary Figure 22**). Strategy-C led to a smaller reduction: amongst younger individuals aged between 40-49, the overall reduction in NNS was 95% (from 8,669 to 404) for strategy-B and 80% (from 8,669 to 1,734) for strategy-C, with an overall increase in NNT of 161% (from 15 to 40) for strategy-B and 43% (from 15 to 22) for strategy-C, respectively (**Supplementary Figure 22**).

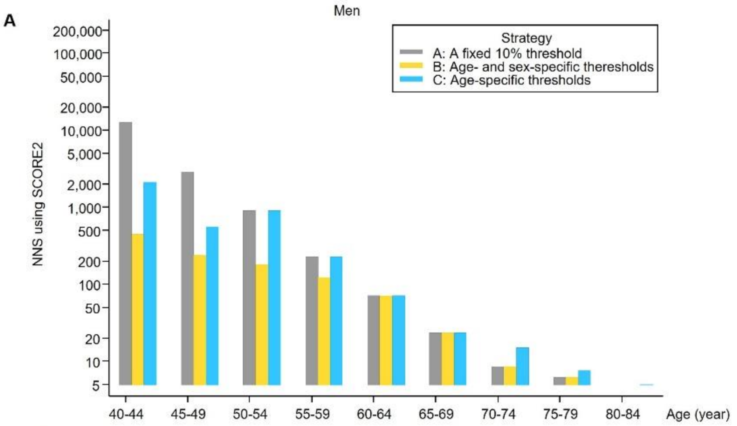

NNS	40-49	50-69	70-85
Strategy A	4,621	83	6
Strategy B	291	66	6
Strategy C	875	83	8

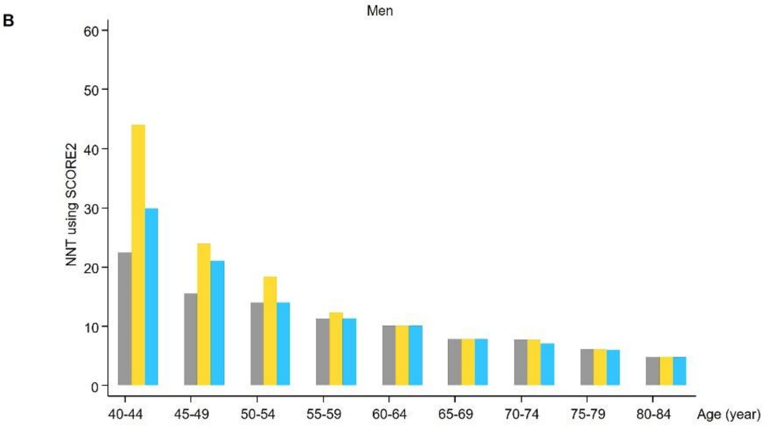

NNT	40-49	50-69	70-85
Strategy A	16	9	6
Strategy B	29	10	6
Strategy C	22	9	6

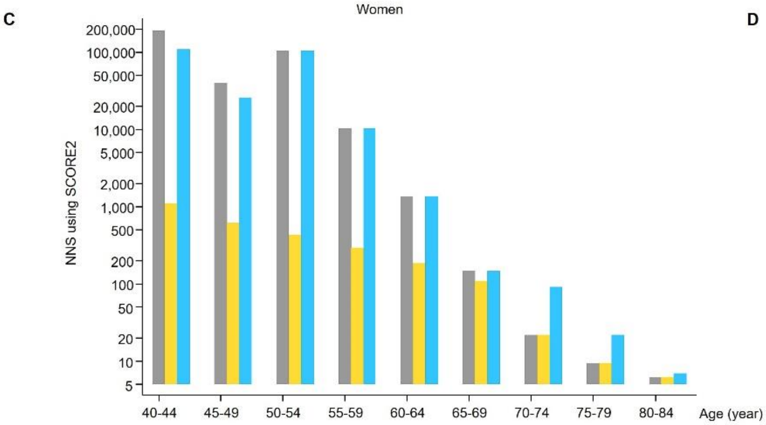

NNS	40-49	50-69	70-85
Strategy A	65,994	718	10
Strategy B	774	203	10
Strategy C	36,749	718	15

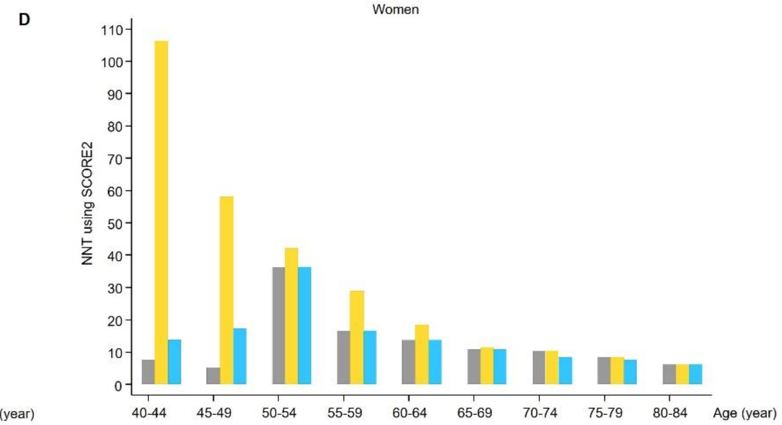

NNT	40-49	50-69	70-85
Strategy A	5	11	7
Strategy B	72	20	7
Strategy C	14	11	6

Supplementary Figure 22. The estimated number needed to screen (NNS) (A and C) and the number needed to treat (NNT) (B and D) to prevent one new CVD event using SCORE2 risk estimations in different stratification strategies by 5-year age group and sex

[a] Numbers needed to screen to prevent one event are shown on the natural log scale for presentation.
[b] Strategy-A identified high-risk individuals as those with an estimated 10-year cardiovascular disease risk ≥10% (a fixed threshold).
[c] Strategy-B identified high-risk individuals as those with an estimated 10-year cardiovascular disease risk ≥10% or ≥90th-percentile of the age- and sex-specific risk distributions (age- and sex-specific thresholds).
[d] Strategy-C identified high-risk individuals as those with an estimated 10-year cardiovascular disease risk ≥7.5%, ≥10%, or ≥15% for individuals in younger (40-49), middle-aged (50-69), and older (70 and over) age groups (age-specific thresholds).

Gain in CVD-free life-expectancy from statin treatment

SCORE2 with strategy-B leads to modest average gains in CVD-free life-years amongst younger individuals in comparison to strategy-A, due to longer benefits from statin treatment given to the younger high-risk population (**Supplementary Figure 23**). The maximum increase in the average gain in CVD-free life expectancy was 0.11 years in men at age 40. On standardising to the population level of England, the population gains in CVD-free life expectancy were also modestly greater under strategy-B versus -A among younger aged people (**Supplementary Figure 24**).

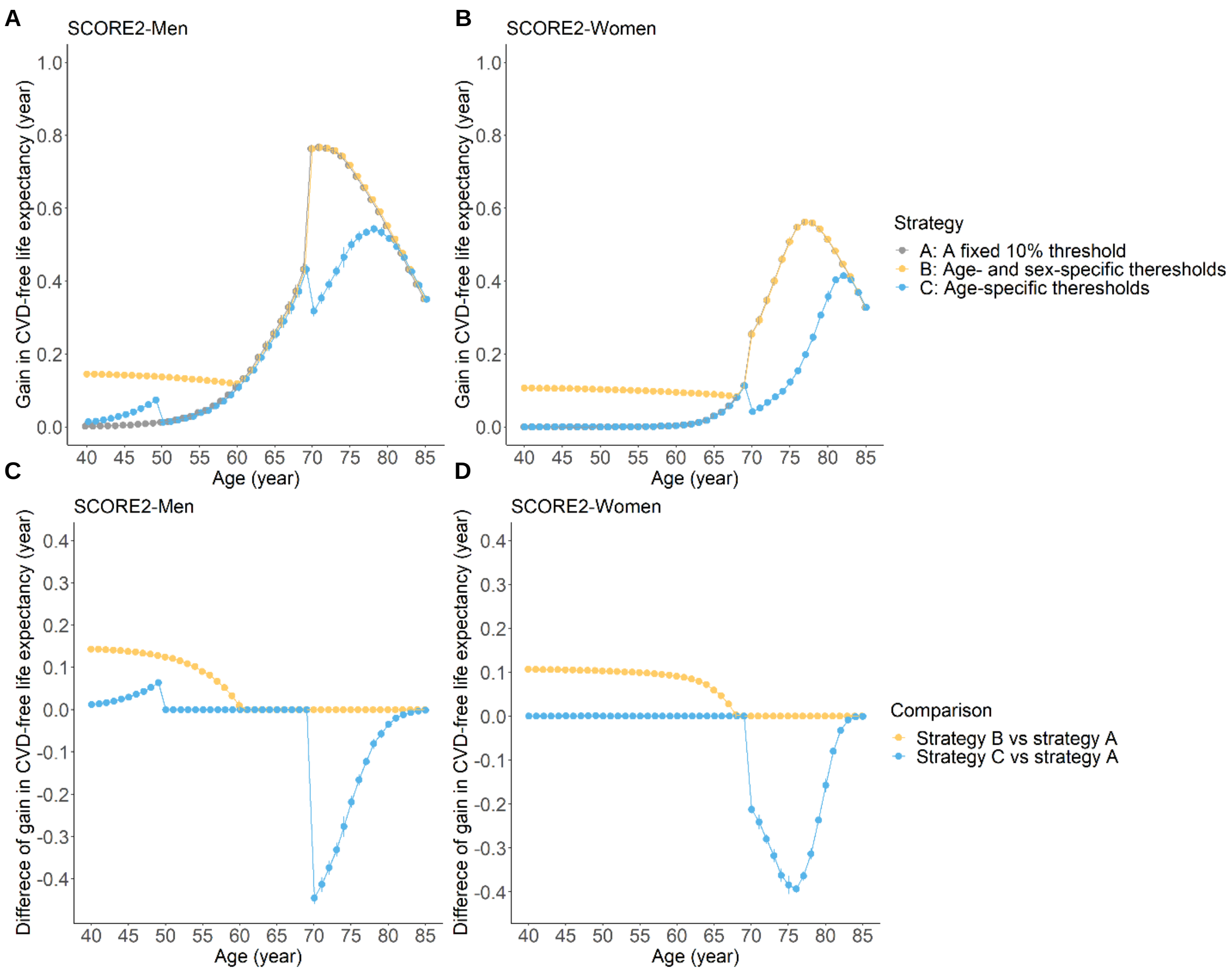

Supplementary Figure 23. Population average gain in CVD-free life-years from statin treatment given to the high-risk population and difference in the gain comparing different stratification strategies using SCORE2 risk estimations

[a] Results are shown as the population average gain in CVD-free life-years (A and B) and the difference in the gain comparing strategies -B and -C versus strategy-A (C and D).

[b] Strategy-A identified high-risk individuals as those with an estimated 10-year cardiovascular disease risk ≥10% (a fixed threshold).

[c] Strategy-B identified high-risk individuals as those with an estimated 10-year cardiovascular disease risk ≥10% or ≥90th-percentile of the age- and sex-specific risk distributions (age- and sex-specific thresholds).

[d] Strategy-C identified high-risk individuals as those with an estimated 10-year cardiovascular disease risk ≥7.5%, ≥10%, or ≥15% for individuals in younger (40-49), middle-aged (50-69), and older (70 and over) age groups (age-specific thresholds).

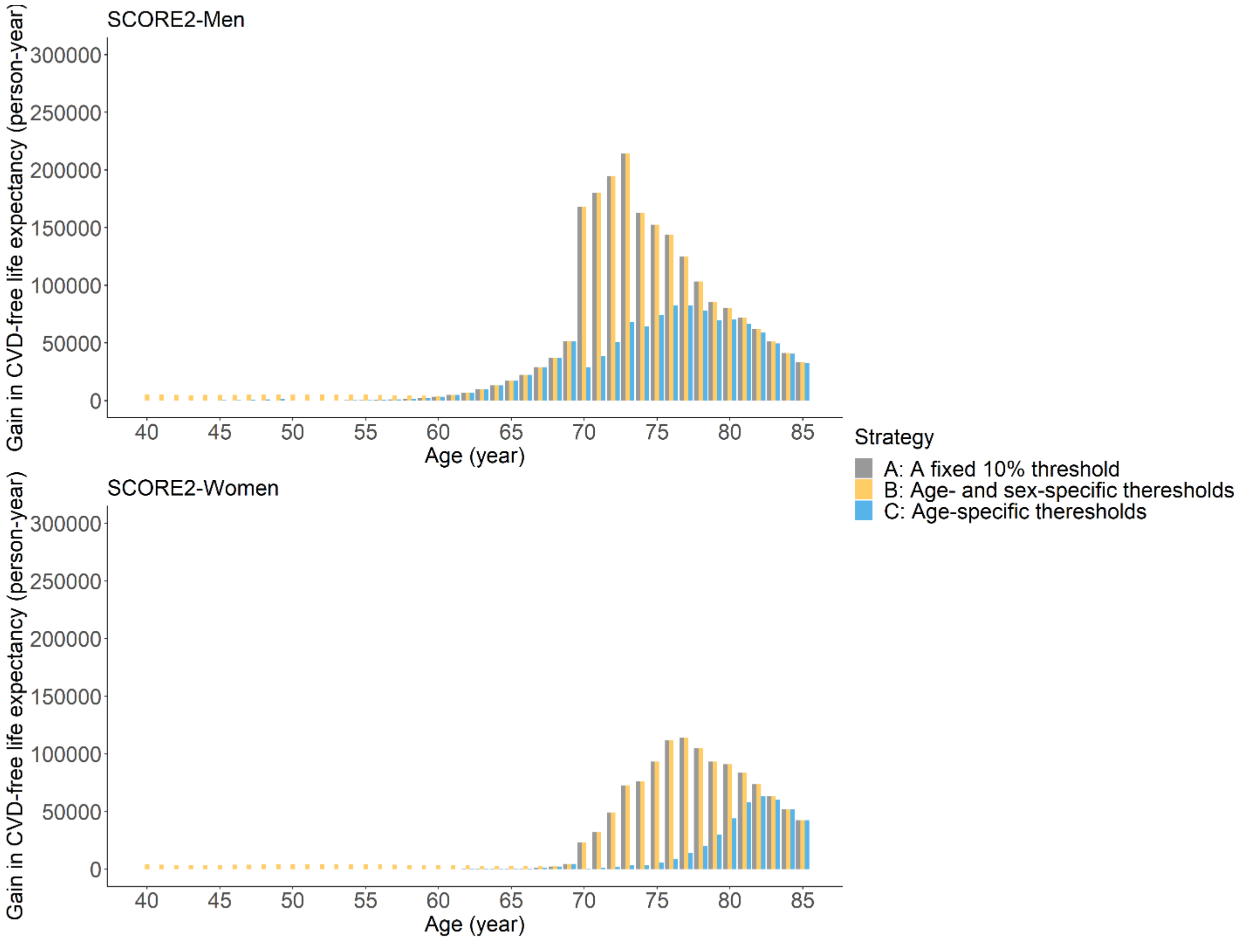

Supplementary Figure 24. Gain in CVD-free life-years (in person-years) from statin treatment given to the high-risk population using different stratification strategies in the standard English population using SCORE2 risk estimations

[a] Strategy-A identified high-risk individuals as those with an estimated 10-year cardiovascular disease risk ≥10% (a fixed threshold).

[b] Strategy-B identified high-risk individuals as those with an estimated 10-year cardiovascular disease risk ≥10% or ≥90th-percentile of the age- and sex-specific risk distributions (age- and sex-specific thresholds).

[c] Strategy-C identified high-risk individuals as those with an estimated 10-year cardiovascular disease risk ≥7.5%, ≥10%, or ≥15% for individuals in younger (40-49), middle-aged (50-69), and older (70 and over) age groups (age-specific thresholds).

Sensitivity analyses

When modelling a fixed budget scenario, constraining the total number of individuals stratified as at high-risk of CVD among the whole population sample to be the same across strategies, a single threshold of 7.9% (strategy-A1) was identified to ascertain the same number of high-risk individuals as that from strategy-B among all individuals. Use of age- and sex-specific thresholds remained favourable compared to a single threshold for individuals aged 40-49, with overall smaller NNS and only a slightly higher NNT (**Supplementary Table 18**). The maximum difference in the average gain in CVD-free life years for strategy-B versus strategy-A1 was 0.13 years in women and 0.15 years in men at age 40 years (**Supplementary Figure 25**). For women aged 64-82 years and men aged 54-84 years, the gain in CVD-free life yeas was smaller when comparing strategy-B vesus strategy-A1, as more individuals were selected as high-risk using the 7.9% threshold in strategy-A1 versus the 10% threshold in strategy-B for those age groups.

Supplementary Table 18. The estimated number needed to screen (NNS) and the number needed to treat (NNT) to prevent one new cardiovascular disease (CVD) event using SCORE2 risk estimations in different stratification strategies in the sensitivity analysis by 10-year age group and sex

	**40-49**		**50-69**		**70-85**	
	**NNS**	**NNT**	**NNS**	**NNT**	**NNS**	**NNT**
**Men**						
Strategy-A [a]	4,621	16	83	9	6	6
Strategy-A1 [b]	1,144	21	38	10	6	6
Strategy-B [c]	291	29	66	10	6	6
Strategy-C [d]	875	22	83	9	8	6
**Women**						
Strategy-A	65,994	5	718	11	10	7
Strategy-A1	42,096	11	210	14	9	8
Strategy-B	744	72	203	20	10	7
Strategy-C	36,749	14	718	11	15	6
**Overall**						
Strategy-A	8,669	15	153	9	7	6
Strategy-A1	2,258	21	65	11	7	7
Strategy-B	404	40	98	12	7	6
Strategy-C	1,734	22	153	9	10	6

[a] Strategy-A identified high-risk individuals as those with an estimated risk ≥10% (a fixed threshold).
[b] Strategy-A1, an alternative strategy in the sensitivity analysis, which identified individuals as high-risk of CVD as those with estimated risk ≥ **7.9%** (a fixed threshold), to ascertain the same total amount of high-risk individuals as strategy-B.
[c] Strategy-B identified high-risk individuals as those with an estimated risk ≥10% or ≥90th-percentile of the age- and sex-specific risk distributions (age- and sex-specific thresholds).
[d] Strategy-C identified high-risk individuals as those with an estimated risk ≥7.5%, ≥10%, or ≥15% for individuals in younger (40-49), middle-aged (50-69), and older (70 and over) age groups (age-specific thresholds).

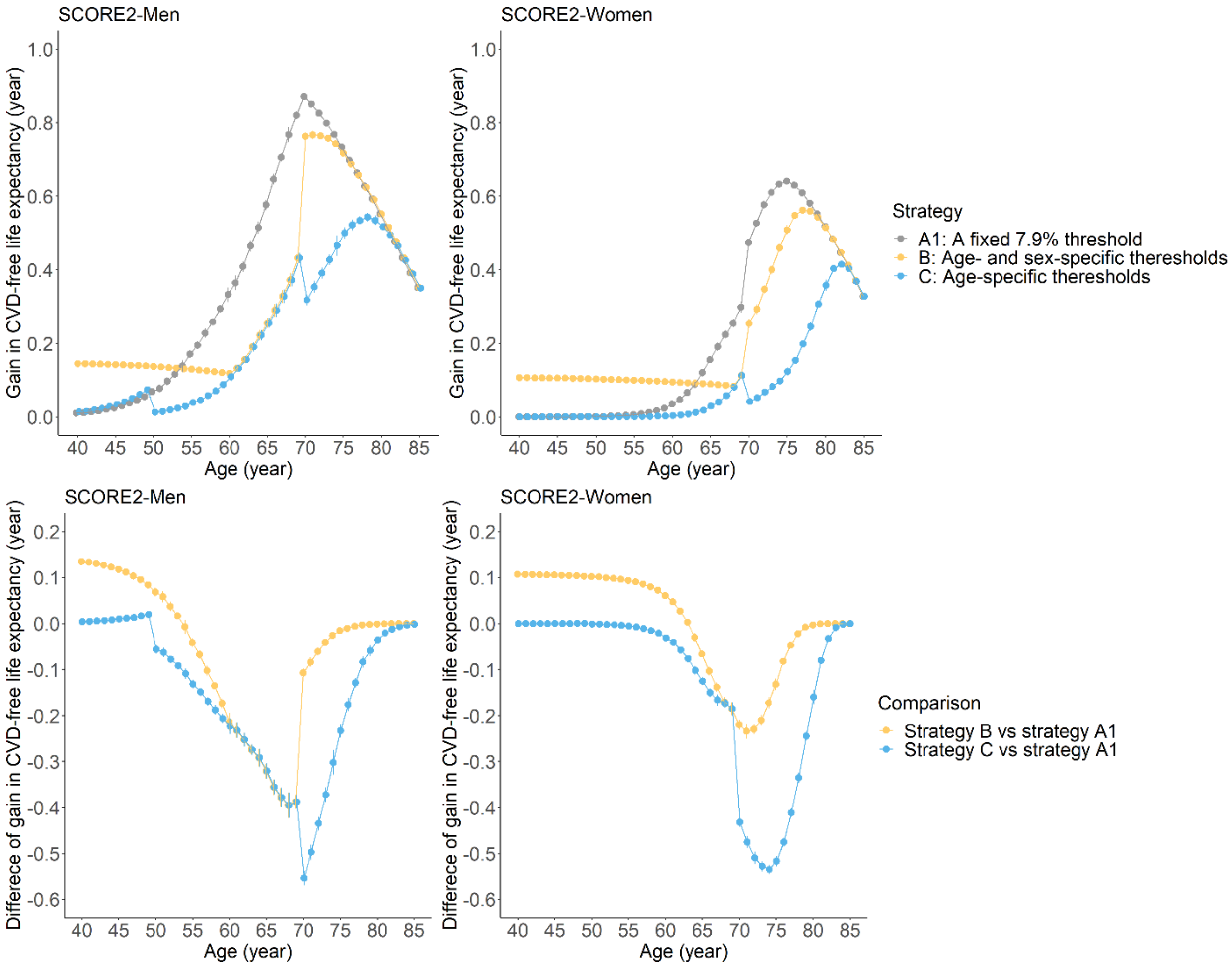

Supplementary Figure 25. Sensitivity analysis - Population average gain in CVD-free life-years from statin treatment given to the high-risk population and difference in the gain comparing different stratification strategies using SCORE2 risk estimations.

[a] Results are shown as the population average gain in CVD-free life-years (A and B) and the difference in the gain comparing strategies -B and -C versus strategy-A (C and D).
[b] Strategy-A1, an alternative strategy in the sensitivity analysis, which identified high-risk individuals of CVD as those with estimated risk ≥ **7.9%** (a fixed threshold), to ascertain the same total amount of high-risk individuals as that from strategy-B.
[c] Strategy-B identified high-risk individuals as those with an estimated 10-year cardiovascular disease risk ≥10% or ≥90th-percentile of the age- and sex-specific risk distributions (age- and sex-specific thresholds).

Supplementary Methods 1: Estimation of QRISK2 and SCORE2 predictions using CPRD data

Data extraction for CVD outcomes and risk factor variables

In QRISK2, incident CVD is defined as new diagnoses of nonfatal or fatal events of coronary heart disease (CHD) (including myocardial infarction and angina), stroke, and transient ischemic attack.[4] Risk factors used in QRISK2 are age, sex, ethnicity, smoking status, systolic blood pressure (SBP), hypertension treatment, ratio of total cholesterol:high-density lipoprotein (HDL) cholesterol, body mass index (BMI), diabetes status, chronic kidney disease status, atrial fibrillation, rheumatoid arthritis, Townsend deprivation score, and family history of CVD.[4]

In SCORE2 and SCORE2-OP, the CVD endpoint is defined as a composite of cardiovascular mortality (including death due to CHD, heart failure, stroke, and sudden death), non-fatal myocardial infarction and non-fatal stroke[2,3] (the models also adjust for competing risks of non-cardiovascular deaths). Risk factors in SCORE2 and SCORE2-OP include age, sex, smoking status, SBP, total- and HDL-cholesterol, and diabetes status.[2,3]

We extracted all these risk factors from CPRD primary care data collected after the population entry date (see **Supplementary Figure 1**) and before the study baseline on 1st January 2006. The following biologically implausible risk factor values were set to missing: SBP >250 mm Hg or <60 mm Hg; total cholesterol >20 mmol/L or <1.75mmol/L; HDL cholesterol >3.1 mmol/L or <0.3 mmol/L.[5,6] Values of SBP, total cholesterol, and HDL cholesterol were standardised using sex-specific means and standard deviations. Note that the Townsend score (a measure of deprivation derived from the post code) is used as continuous variable in QRISK2, but in the CPRD dataset only the categorical values (quintile, decile, or 'twentile') are available[7] and only quintiles are provided for the linked data in this study. Therefore, in order to calculate QRISK2 estimates using CPRD data, we used the median value for each quintile reported from national census data.[7–9]

Estimation of risk factor values using Multiple Imputation by Chained Equations

For risk factors including hypertension treatment, diabetes status, chronic kidney disease status, atrial fibrillation, and rheumatoid arthritis, the most recently observed treatment or diseases status measured before the study baseline on 1st January 2006 were used. Individuals were assumed to

have hypertension treatment or these diseases for the rest of follow-up after their first prescription or diagnosis.

For other risk factors including SBP, total cholesterol, HDL cholesterol, BMI, smoking status, and Townsend score, missing values are common in CPRD primary care EHRs. **Multiple Imputation by Chained Equations (MICE)**[10,11] was used to impute the missing values for these six variables from a set of imputation models. Imputation was carried out separately by sex. The imputation models included all QRISK2 predictor variables listed above (as SCORE2 predictors are a subset of QRISK2 predictors), with the most recent observed values measured before the baseline. The imputation models also included the outcome indicators for both QRISK2 CVD outcome and SCORE2 CVD outcome, the Nelson-Aalen estimators of the baseline cumulative hazard for each CVD outcome, together with the age interaction terms with all other covariates. Missing values for continuous factors (SBP, total cholesterol, HDL cholesterol, BMI) were imputed from linear regression models; missing values for a binary variable (smoking status) were imputed from a logistic model; and missing values for an ordinal variable (Townsend score) were imputed from an ordered logistic regression model. Log transformed values of SBP, total cholesterol, HDL cholesterol, and BMI were used in the imputation models to get better estimation of realistic values that were not normally distributed, and then back transformed for the analyses. Five imputations were performed which are adequate to get relatively high efficiency[12] and are pragmatic for this sample size. Multiple imputation was performed using the mi package in Stata 15.1 (StataCorp LLC, College Station, Texas).

Subsequent analyses for calculating model performance metrics, stratification strategies evaluation, and public health modelling were conducted in each imputed dataset and then pooled across imputations using **Rubin's rules**.[12,13]

Calculation of predicted 10-year CVD risk

Subsequently, the 10-year risk of CVD events for each individual was calculated separately for each of QRISK2 and SCORE2 CVD outcomes, using the observed and imputed (when missing) risk factor and outcome variables from the previous steps, with hazard ratio coefficients provided in the QRISK2,[4] and SCORE2 and SCORE2-OP[2,3] equations. When using SCORE2 and SCORE2-OP, parameters derived and recalibrated for the low-risk countries were used because the UK is stratified as the low-risk country group in SCORE2.

Model validation

The validation of model predictive performance was examined. R^2, the percentage of variation in time-to-CVD-event outcomes explained by the prediction model, was calculated where higher values indicate more explained variation and better performance.[14] Discrimination was measured by Harrell's C statistic, which is a rank-order measure to quantify the discriminative ability to rank individuals according to their predicted and observed risk of CVD accounting for the survival time.[15,16] A C-index value of 1 means perfect discrimination, and value of 0.5 means by chance alone. We also calculated D statistic, another measure of discrimination which is the log hazard ratio comparing the two equal-sized prognostic groups.[17] Higher D statistic values indicate better discrimination. Calibration, which displays the agreement between observed outcomes and predicted risk,[18] was assessed graphically by plots comparing the mean predicted 10-year risk vs. the observed 10-year CVD outcomes by tenths of predicted risk. We also presented the predicted and observed 10-year CVD risks by 5-year age group. For QRISK2 calibration, the observed outcomes were obtained from the Kaplan-Meier estimates close to 10 years.[16,19,20] For SCORE2 calibration, since the predicted risk was estimated accounting for competing risks of non-CVD death, the observed 10-year CVD risk in SCORE2 was estimated using cumulative incidence function (CIF) at 10 years which adjusted for non-CVD death. All these predictive performance measurements were calculated accounting for censoring.

Supplementary Methods 2: The closed form calculation of counterfactual survival times in the absence of statin initiation

The estimated CVD risks are used to inform treatment initiation for those with high predicted risk. However, although we had excluded those who had statins at baseline, there were still 21% of the study population started statin therapy during follow-up (so-called “treatment drop-ins”)[21,22] and might under estimate the statin-free CVD risk in individuals who went on to initiate statins.[23] Therefore, we first estimated the counterfactual follow-up time assuming no one had statins during follow-up to account for the “treatment drop-ins” effect.[24] Counterfactual survival times were estimated as follows.

Denote t as the observed time-to-CVD-event (or censoring time), let t_s be the time-to-statin-initiation (or equal to t if not observed) and let t^* be the counterfactual statin-naïve time-to-CVD-event.

The cumulative hazard function for t, using the Weibull model can be written as:

$$H(t) = \int_0^t h_{0S}(u)\exp[{\beta_x}^T X(La) \ + B \times Statin(u)\ ]\, du$$

$$= \int_0^{t_s} h_{0S}(u)\exp\left({\beta_x}^T X(La)\right)du + \int_{t_s}^{t} h_{0S}(u)\exp[{\beta_x}^T X(La) + B]du$$

$$= \ \exp\left({\beta_x}^T X(La)\right) \times \left[\int_0^{t_s} h_{0S}(u)du + \exp(B)\int_{t_s}^{t} h_{0S}(u)\ du\right]$$

$$= \exp\left({\beta_x}^T X(La)\right) \times \{H_0(t_s) + \exp(B) \times \ [H_0(t) - H_0(t_s)]\}.$$

The cumulative hazard function for t*, using the Weibull model can be written as:

$$H(t^*) = \int_0^{t^*} h_{0S}(u)\exp\,({\beta_x}^T X(La))\, du = \ \exp\,({\beta_x}^T X(La)) \times H_0(t^*)$$

Under the proportional-hazards assumption for the effect of statins, we assume equality of the cumulative hazard function for t and t^*, because the model with observed population and observed follow-up time with additionally adjusted for time-dependent statin use should be equal to the estimation from the counterfactual population with no one had statins using the counterfactual time. Consequently,

$$\exp\,(\beta_x{}^T X(La)) \times H_0(t^*) = \exp\big(\beta_x{}^T X(La)\big) \times \{H_0(t_s) + \exp(B) \times\ [H_0(t) - H_0(t_s)]\}$$

$$H_0(t^*) =\ \{H_0(t_s) + \exp(B) \times\ [H_0(t) - H_0(t_s)]\}$$

$$\lambda t^{*\nu} = \lambda t_s{}^{\nu} + \exp(B) \times (\lambda t^{\nu} - \lambda t_s{}^{\nu})$$

$$t^* = [t_s{}^{\nu} +\ \exp(B) \times (t^{\nu} - t_s{}^{\nu})]^{1/\nu}.$$

When $\nu = 1$, (i.e., the exponential model) and assuming a 25% reduction in risk by statins (estimated results from clinical trials[25,26]), then intuitively, $t^* = t_s +\ 0.75 * (t - t_s)$.

It is not possible to formulate a closed form calculation for $t^*$for the Cox model, however, the cumulative hazard $H_0(t^*)$ can be estimated, from which an estimate of t^* could be approximated from the observed times t, either assuming step functions between observed event times (fine in large datasets with many unique observed event times) or after applying some smoothing function.

Supplementary Methods 3: Calculation of sensitivity, specificity, and AUROC-dp accounting for censoring

Sensitivity refers to the proportion of individuals who experienced an incident CVD event and were correctly grouped as high-risk by the stratification strategy; specificity refers to the proportion of individuals who did not develop a CVD event and were correctly identified as low-risk.[27] The calculation of sensitivity and specificity was adjusted for censoring using the inverse probability of censoring weighted (IPCW) estimates based on Kaplan-Meier estimator weighting method.[28] The adapted Area Under Receiver Operating Characteristic curve for dichotomised predictions (AUROC-dp), which incorporates sensitivity and specificity, represents the ability to discriminate between those who did and who did not have a CVD event according to the combined risk prediction model *and* the stratification rule. With the binary predictor (i.e., high-risk or low-risk of CVD), the AUROC-dp was calculated as the proportion of all possible concordant pairs plus half the proportion of ties while taking into account the time-to-event nature of the data (using `somersd` package in Stata software with time-to-event/censoring included in the calculation).[29–31] Higher AUROC-dp values indicate that individuals who experienced a CVD event are more likely to be identified as high-risk under the stratification rules than those who did not have CVD events.

Supplementary Methods 4: Estimation of net benefit for different risk stratification strategies

Net benefit was calculated using the following equation at each age group:

$$\text{Net benefit} = \text{True positive rate} - \text{False positive rate} \times \left(\frac{P}{1-P}\right)$$

where P is the threshold probability to define when the individual is at high risk of developing CVD (i.e., the risk thresholds under each stratification strategy).[32] Accounting for time-to-event data, the true positive rate is given by [1-(S(t)| x = 1)]×P(x = 1) and the false positive rate is given by (S(t)| x = 1)×P(x = 1), where x = 1 represents that the individual had a predicted risk greater than the threshold probability P and x = 0 otherwise; S(t) is the Kaplan-Meier survival probability at the chosen time t (which is 10 years in our calculation).[33] One assumption of the method is that the mechanism of censoring is independent from the predictors used in the risk prediction model.[33]

Supplementary Methods 5: Calculation of the public health modeling metrics

The number needed to screen (NNS) and the number needed to treat (NNT) to prevent one new CVD event in 10 years were estimated from the number of new CVD events that could be prevented in the population (NEPP). When allocating statins to the high-risk population stratified using each risk stratification strategy, the expected number of new CVD events prevented was calculated from:

NEPP = Number of individuals who had CVD over the next 10 years and exceeded statin treatment threshold (i.e., high-risk people among the cases) (N) × Proportion who adhere to treatment (Pa) × Relative risk reduction (RRR) of CVD risk associated with statins[34]

The following assumptions were made for modelling the NEPP:

Assumption 1: The compliance with allocated statin treatment was associated with age and sex, with younger and female individuals having lower adherence.[35] The proportion of adherence to statin treatment (Pa) was assumed to be 70%[36] for the reference group (women aged 55 to 64 years old), and the adherence for other age- and sex-groups were estimated using the relative risk of association with high adherence to statins previously reported[35] and shown in **Supplementary Table 19**.

Supplementary Table 19. Estimated proportion of adherence to statin therapy by age and sex

Age group	40-44	45-54	55-64	65-70	71-75	76-80	81-85	85+
RR [a] for women	0.71	0.86	1 (Reference)	1.10	1.05	1.01	1.02	0.98
RR for men	0.83	1.01	1.17	1.29	1.23	1.18	1.19	1.15
Pa [b] for Women	50%	60%	70% [c]	77%	74%	71%	71%	69%
Pa Men	59%	70%	82%	90%	87%	83%	83%	81%

[a] RR: relative risk of association with high adherence to statin therapy reported in previous publication[35]
[b] Pa: proportion of people adhering to treatment
[c] The adherence to statin treatment was assumed to be 70% [36] for the referent group.

Assumption 2: The relative risk reduction of CVD risk associated with statins was assumed constant across age and sex.[37,38] After accounting for the adherence rate, the statin treatment effect on CVD risk reduction was set as 31% based on published literature.[36] The value of 31% used to calculate NEPP because it was assumed to be the relative risk reduction (RRR) without adjustment for compliance rate,[36] thus it was used with the combination of various proportions

of adherence (Pa) listed in **Supplementary Table 19** for different populations. Whereas the 25% statin treatment effect reported in clinical trials[25,26] was used for calculation of “statin-naïve” follow-up time described in **Supplementary Methods 2.** In that situation, we assumed that the adherence to statin therapy was adequately captured in trials with intention-to-treat analyses.[39] (Similar results were found when using either combining the 31% relative risk reduction with adherence rate or simply using the 25% relative risk reduction from trial results.)

Assumption 3: The relative risk reduction maintains constant from the initiation to the remaining follow-up years.[39]

The number needed to screen (NNS) to prevent one new CVD event = Number of target population / NEPP

The number needed to treat (NNT) to prevent one new CVD event = Number of high-risk individuals / NEPP

Supplementary Methods 6: Estimation of gain in CVD-free life expectancy from statin treatment

To investigate the lifelong-term benefit from treating the high-risk population with statins, we assessed the gain in CVD-free life expectancy associated with the statin initiation. The CVD-free life expectancy (or life-years free of CVD) is defined as the average duration of survival over the follow-up period, calculated by using the numerical integration of the CVD-free survival probabilities and the time of remaining CVD-free life-years.[40] Graphically, this is equal to the area under the CVD-free survival curve.[40] In this study, we estimated the CVD-free life expectancy to 95 years of age because the maximum of follow-up age in the linked data is 95 and few individuals survived after that age. In order to reflect the true remaining lifetime CVD estimates over the lifelong term, especially for the younger individuals with longer follow-up, we calculated the CVD-free life expectancy with adjustment for the competing risk of death from non-CVD events. Estimations were based on sex-specific lifetables combining both CVD risk and competing risk with 1-year age intervals.[41]

At each life-year, first, the cause-specific 1-year survival for both CVD events and non-CVD was calculated among the whole study population. Then we estimated the cumulative survival by

multiplying the survival probability of that life-year (**1 – CVD risk – non-CVD death risk**) by the survival probability at the beginning of each life-year,[41] repeating to 95 years of age.

When identified as high-risk individuals by each stratification strategy using the predicted risk at baseline, for the remaining cumulative survival for each life-year, the 1-year risk of CVD was calculated by inserting the relative risk reduction of statin treatment on CVD into the sub-distribution survival function of CVD events (i.e., **1- (age-specific 1-year CVD baseline survival)^exp(0.75)**).[41]

In addition, the life expectancy was calculated with further adjustment for a time preference rate to account for the increasing lower value that patients currently give to the life years further out into the far-off future. [42]

Life expectancy with time preference r = $\frac{1}{r} \times [1 - \frac{1}{(1+r)^{LE}}]$,

where r is the time preference, LE is the life expectancy without adjustment for time preference. [42] A time preference rate of 0.03 was used in this study, which values the next year as worth 97% of the previous year. The preference rate was applied throughout the remaining estimated life expectancy. [42]

Gain in life-years free of CVD is the difference between the CVD-free life expectancy of initiating and not initiating statin therapy.

Notably, we made the same assumptions as used in **Supplementary Methods 3** (Assumptions 1 to 3) for the estimation of lifetime benefit from statin treatment, with an additional assumption:

Assumption 4: No statin treatment effect for the sub-distribution of survival function of the competing events.

In order to illustrate the results intuitively at a population level, we calculated the population CVD-free life-years gained using the most recent available data on age- and sex-standard English population structure in 2020 mid-year (data released in 2021).[43]

For example, for men/women with baseline age of i years, the CVD-free life-years gained in the standard English people at age i =

$$\left(\frac{\text{Mi} \times \text{Nhi}}{\text{Ni}}\right) \times \text{Nsi}$$

where Mi represents the average gain in CVD-free life-years among men/women with baseline age of i in the analysed dataset, Nhi is the number of high-risk people among men/women with baseline age of i in the analysed dataset, Ni is the total number of men/women with baseline age of i in the analysed dataset, and Nsi is the number of men/women at age i from the standard English population.

STROBE Statement - checklist of items that should be included in reports of observational studies

	Item No	Recommendation	Page No in the main manuscript
**Title and abstract**	1	(*a*) Indicate the study's design with a commonly used term in the title or the abstract	3
		(*b*) Provide in the abstract an informative and balanced summary of what was done and what was found	3
**Introduction**			
Background/rationale	2	Explain the scientific background and rationale for the investigation being reported	6-7
Objectives	3	State specific objectives, including any prespecified hypotheses	7
**Methods**			
Study design	4	Present key elements of study design early in the paper	7-8
Setting	5	Describe the setting, locations, and relevant dates, including periods of recruitment, exposure, follow-up, and data collection	7-8
Participants	6	(*a*) *Cohort study*—Give the eligibility criteria, and the sources and methods of selection of participants. Describe methods of follow-up<br>*Case-control study*—Give the eligibility criteria, and the sources and methods of case ascertainment and control selection. Give the rationale for the choice of cases and controls<br>*Cross-sectional study*—Give the eligibility criteria, and the sources and methods of selection of participants	7-8
		(*b*) *Cohort study*—For matched studies, give matching criteria and number of exposed and unexposed<br>*Case-control study*—For matched studies, give matching criteria and the number of controls per case	Not applicable
Variables	7	Clearly define all outcomes, exposures, predictors, potential confounders, and effect modifiers. Give diagnostic criteria, if applicable	8; Supplementary Methods 1
Data sources/ measurement	8*	For each variable of interest, give sources of data and details of methods of assessment (measurement). Describe comparability of assessment methods if there is more than one group	7-8; Supplementary Methods 1

Bias	9	Describe any efforts to address potential sources of bias	8; Supplementary Methods 1
Study size	10	Explain how the study size was arrived at	7-8; Supplementary Figure 1
Quantitative variables	11	Explain how quantitative variables were handled in the analyses. If applicable, describe which groupings were chosen and why	8; Supplementary Methods 1
Statistical methods	12	(*a*) Describe all statistical methods, including those used to control for confounding	8-12
		(*b*) Describe any methods used to examine subgroups and interactions	9
		(*c*) Explain how missing data were addressed	8; Supplementary Methods 1
		(*d*) *Cohort study*—If applicable, explain how loss to follow-up was addressed<br>*Case-control study*—If applicable, explain how matching of cases and controls was addressed<br>*Cross-sectional study*—If applicable, describe analytical methods taking account of sampling strategy	8; Supplementary Methods 1
		(*e*) Describe any sensitivity analyses	11-12

Continued on next page

**Results**			
Participants	13*	(a) Report numbers of individuals at each stage of study—eg numbers potentially eligible, examined for eligibility, confirmed eligible, included in the study, completing follow-up, and analysed	13; Supplementary Figure 1
		(b) Give reasons for non-participation at each stage	Supplementary Figure 1
		(c) Consider use of a flow diagram	Supplementary Figure 1
Descriptive data	14*	(a) Give characteristics of study participants (eg demographic, clinical, social) and information on exposures and potential confounders	13
		(b) Indicate number of participants with missing data for each variable of interest	Supplementary Table 1
		(c) *Cohort study*—Summarise follow-up time (eg, average and total amount)	13
Outcome data	15*	*Cohort study*—Report numbers of outcome events or summary measures over time	13
		Case-control study—Report numbers in each exposure category, or summary measures of exposure	Not applicable
		Cross-sectional study—Report numbers of outcome events or summary measures	Not applicable
Main results	16	(*a*) Give unadjusted estimates and, if applicable, confounder-adjusted estimates and their precision (eg, 95% confidence interval). Make clear which confounders were adjusted for and why they were included	Not applicable
		(*b*) Report category boundaries when continuous variables were categorized	Supplementary Methods 1
		(*c*) If relevant, consider translating estimates of relative risk into absolute risk for a meaningful time period	Not applicable
Other analyses	17	Report other analyses done—eg analyses of subgroups and interactions, and sensitivity analyses	16
**Discussion**			
Key results	18	Summarise key results with reference to study objectives	17
Limitations	19	Discuss limitations of the study, taking into account sources of potential bias or imprecision. Discuss both direction and magnitude of any potential bias	20-21
Interpretation	20	Give a cautious overall interpretation of results considering objectives, limitations, multiplicity of analyses, results from similar studies, and other relevant evidence	17-19
Generalisability	21	Discuss the generalisability (external validity) of the study results	17-19
**Other information**			

Funding	22	Give the source of funding and the role of the funders for the present study and, if applicable, for the original study on which the present article is based	23

*Give information separately for cases and controls in case-control studies and, if applicable, for exposed and unexposed groups in cohort and cross-sectional studies.

Note: An Explanation and Elaboration article discusses each checklist item and gives methodological background and published examples of transparent reporting. The STROBE checklist is best used in conjunction with this article (freely available on the Web sites of PLoS Medicine at http://www.plosmedicine.org/, Annals of Internal Medicine at http://www.annals.org/, and Epidemiology at http://www.epidem.com/). Information on the STROBE Initiative is available at www.strobe-statement.org.

References

1 Austin PC. Balance diagnostics for comparing the distribution of baseline covariates between treatment groups in propensity-score matched samples. *Stat Med*. 2009;28:3083–107.

2 SCORE2 working group and ESC Cardiovascular risk collaboration. SCORE2 risk prediction algorithms: new models to estimate 10-year risk of cardiovascular disease in Europe. *European Heart Journal*. 2021;42:2439–54.

3 SCORE2-OP working group and ESC Cardiovascular risk collaboration. SCORE2-OP risk prediction algorithms: estimating incident cardiovascular event risk in older persons in four geographical risk regions. *European Heart Journal*. 2021;42:2455–67.

4 Hippisley-Cox J, Coupland C, Vinogradova Y, *et al.* Predicting cardiovascular risk in England and Wales: prospective derivation and validation of QRISK2. *BMJ*. 2008;336:1475–82.

5 Littman AJ, Boyko EJ, McDonell MB, *et al.* Evaluation of a weight management program for veterans. *Prev Chronic Dis*. 2012;9.

6 Hajifathalian K, Ueda P, Lu Y, *et al.* A novel risk score to predict cardiovascular disease risk in national populations (Globorisk): a pooled analysis of prospective cohorts and health examination surveys. *Lancet Diabetes Endocrinol*. 2015;3:339–55.

7 Wolf A, Dedman D, Campbell J, *et al.* Data resource profile: Clinical Practice Research Datalink (CPRD) Aurum. *International Journal of Epidemiology*. 2019;48:1740–1740g.

8 Office for National Statistics, National Records of Scotland, Northern Ireland Statistics and Research Agency, *et al.* 2011 UK Townsend Deprivation Scores. 2017. https://doi.org/10.5257/CENSUS/AGGREGATE-2011-2

9 Hippisley-Cox J, Coupland C, Brindle P. The performance of seven QPrediction risk scores in an independent external sample of patients from general practice: a validation study. *BMJ Open*. 2014;4:e005809.

10 van Buuren S. Multiple imputation of discrete and continuous data by fully conditional specification. *Stat Methods Med Res*. 2007;16:219–42.

11 Buuren S van, Groothuis-Oudshoorn K. mice: Multivariate Imputation by Chained Equations in R. *Journal of Statistical Software*. 2011;45:1–67.

12 White IR, Royston P, Wood AM. Multiple imputation using chained equations: Issues and guidance for practice. *Statistics in Medicine*. 2011;30:377–99.

13 Rubin DB. *Multiple imputation for nonresponse in surveys*. John Wiley & Sons 2004.

14 Royston P. Explained Variation for Survival Models. *The Stata Journal*. 2006;6:83–96.

15 Harrell FE, Lee KL, Mark DB. Multivariable prognostic models: issues in developing models, evaluating assumptions and adequacy, and measuring and reducing errors. *Statistics in Medicine*. 1996;15:361–87.

16 Steyerberg EW, Vickers AJ, Cook NR, *et al.* Assessing the performance of prediction models: a framework for traditional and novel measures. *Epidemiology*. 2010;21:128–38.

17 Graf E, Schmoor C, Sauerbrei W, *et al.* Assessment and comparison of prognostic classification schemes for survival data. *Stat Med*. 1999;18:2529–45.

18 Hilden J, Habbema JD, Bjerregaard B. The measurement of performance in probabilistic diagnosis. II. Trustworthiness of the exact values of the diagnostic probabilities. *Methods Inf Med*. 1978;17:227–37.

19 Steyerberg E. *Clinical prediction models: a practical approach to development, validation, and updating*. New York: Springer-Verlag 2009.

20 Collins GS, Altman DG. An independent and external validation of QRISK2 cardiovascular disease risk score: a prospective open cohort study. *BMJ*. 2010;340:c2442.

21 Pajouheshnia R, Damen JAAG, Groenwold RHH, *et al.* Treatment use in prognostic model research: a systematic review of cardiovascular prognostic studies. *Diagnostic and Prognostic Research*. 2017;1:15.

22 Liew SM, Doust J, Glasziou P. Cardiovascular risk scores do not account for the effect of treatment: a review. *Heart*. 2011;97:689–97.

23 Groenwold RHH, Moons KGM, Pajouheshnia R, *et al.* Explicit inclusion of treatment in prognostic modeling was recommended in observational and randomized settings. *J Clin Epidemiol*. 2016;78:90–100.

24 Xu Z, Arnold M, Stevens D, *et al.* Prediction of cardiovascular disease risk accounting for future initiation of statin treatment. *American Journal of Epidemiology*. Published Online First: 17 February 2021.

25 Cook NR, Ridker PM. Further insight into the cardiovascular risk calculator controversy: The roles of statins, revascularizations, and under-ascertainment in the Women's Health Study. *JAMA Intern Med*. 2014;174:1964–71.

26 Cholesterol Treatment Trialists' (CTT) Collaborators, Mihaylova B, Emberson J, *et al.* The effects of lowering LDL cholesterol with statin therapy in people at low risk of vascular disease: meta-analysis of individual data from 27 randomised trials. *Lancet*. 2012;380:581–90.

27 Florkowski CM. Sensitivity, specificity, Receiver-Operating Characteristic (ROC) curves and likelihood ratios: communicating the performance of diagnostic tests. *Clin Biochem Rev*. 2008;29:S83–7.

28 Heagerty PJ, Lumley T, Pepe MS. Time-dependent ROC curves for censored survival data and a diagnostic marker. *Biometrics*. 2000;56:337–44.

29 Zou KH, O'Malley AJ, Mauri L. Receiver-Operating Characteristic analysis for evaluating diagnostic tests and predictive models. *Circulation*. 2007;115:654–7.

30 Muschelli J. ROC and AUC with a binary predictor: a potentially misleading metric. *J Classif*. 2020;37:696–708.

31 Newson RB. Comparing the predictive powers of survival models using Harrell's C or Somers' D. *The Stata Journal*. 2010;10:339–58.

32 Vickers AJ, Van Calster B, Steyerberg EW. Net benefit approaches to the evaluation of prediction models, molecular markers, and diagnostic tests. *BMJ*. 2016;352:i6.

33 Vickers AJ, Cronin AM, Elkin EB, *et al.* Extensions to decision curve analysis, a novel method for evaluating diagnostic tests, prediction models and molecular markers. *BMC Med Inform Decis Mak*. 2008;8:53.

34 Gemmell I, Heller RF, Payne K, *et al.* Potential population impact of the UK government strategy for reducing the burden of coronary heart disease in England: comparing primary and secondary prevention strategies. *Qual Saf Health Care*. 2006;15:339–43.

35 Colantonio LD, Rosenson RS, Deng L, *et al.* Adherence to Statin Therapy Among US Adults Between 2007 and 2014. *Journal of the American Heart Association*. 2019;8:e010376.

36 Chamnan P, Simmons RK, Khaw K-T, *et al.* Estimating the population impact of screening strategies for identifying and treating people at high risk of cardiovascular disease: modelling study. *BMJ*. 2010;340:c1693.

37 Baigent C, Keech A, Kearney PM, *et al.* Efficacy and safety of cholesterol-lowering treatment: prospective meta-analysis of data from 90,056 participants in 14 randomised trials of statins. *Lancet*. 2005;366:1267–78.

38 Brugts JJ, Yetgin T, Hoeks SE, *et al.* The benefits of statins in people without established cardiovascular disease but with cardiovascular risk factors: meta-analysis of randomised controlled trials. *BMJ*. 2009;338:b2376.

39 Ferket BS, van Kempen BJH, Heeringa J, *et al.* Personalized Prediction of Lifetime Benefits with Statin Therapy for Asymptomatic Individuals: A Modeling Study. *PLoS Med*. 2012;9.

40 Dehbi H-M, Royston P, Hackshaw A. Life expectancy difference and life expectancy ratio: two measures of treatment effects in randomised trials with non-proportional hazards. *BMJ*. 2017;357:j2250.

41 Jaspers NEM, Blaha MJ, Matsushita K, *et al.* Prediction of individualized lifetime benefit from cholesterol lowering, blood pressure lowering, antithrombotic therapy, and smoking cessation in apparently healthy people. *Eur Heart J*. 2020;41:1190–9.

42 Liew SM, Jackson R, Mant D, *et al.* Should identical CVD risks in young and old patients be managed identically? Results from two models. *BMJ Open*. 2012;2:e000728.

43 Office for National Statistics. Estimates of the population for the UK, England and Wales, Scotland and Northern Ireland. https://www.ons.gov.uk/peoplepopulationandcommunity/populationandmigration/populationestimates/datasets/populationestimatesforukenglandandwalesscotlandandnorthernireland (accessed 23 July 2022)
