# Supplementary material for: Age and sex specific thresholds for risk stratification of cardiovascular disease and clinical decision making: prospective open cohort study
Source: BMJ Med. 2024 Aug 12;3(1):e000633. doi: 10.1136/bmjmed-2023-000633 (PMC11340247; doi:10.1136/bmjmed-2023-000633)
Supplement: Supplementary file 2 [file bmjmed-2023-000633.reviewer_comments.pdf]

PEER REVIEW HISTORY

BMJ Medicine publishes all reviews undertaken for accepted manuscripts. Reviewers are asked to complete a checklist review form and are provided with free text boxes to elaborate on their assessment. These free text comments are reproduced below.

This paper was submitted to a another journal from BMJ but declined for publication following peer review. The authors addressed the reviewers' comments and submitted the revised paper to BMJ Medicine. The paper was subsequently accepted for publication at BMJ Medicine.

ARTICLE DETAILS

**TITLE (PROVISIONAL)**	Age- and sex-specific thresholds for cardiovascular disease risk stratification and clinical decision-making
**AUTHORS**	Xu, Zhe; Usher-Smith, Juliet; Pennells, Lisa; Chung, Ryan; Arnold, Matthew; Kim, Lois; Kaptoge, Stephen; Sperrin, Matthew; Di Angelantonio, Emanuele; Wood, Angela

VERSION 1 - REVIEW

**REVIEWER 1**	Reviewer 1. Competing Interrest: None
**REVIEW RETURNED**	20-Dec-2022

**GENERAL COMMENTS**	Publish - yes<br>This article is complex to read unless the reader has a substantial statistical background. Given this, a little simplification may be of benefit to a wider readership. Integrating the abstract with a version of the text found on page 6, lines 11 to 16 would, I believe, assist the reader. The article proposed a strategy which utilises the top 90th percentiles from age and sex specific risk distributions to illustrate potential advantages of applying age and sex thresholds in CVD risk stratification. In this the article is successful.<br>From a patient perspective the nagging question relates to how a GP would actually use the findings. Could a simplified age by gender lookup table with the necessary caveats be of assistance in the decision to utilise statins? The life-years gain with statin initiation for men aged 40 years was stated as 0.16 years (page 4, line 1). This observation is not discussed in any detail elsewhere in the article so could be considered an unnecessary observation. While acknowledged by the authors, life-style, diet, ethnicity and medication adherence over time are important factors which could limit the successful outcome at the GP level. Adherence rates are briefly mentioned and should be discussed further in terms of impacts on strategies A and B. More discussion of these concerns would benefit the reader. An issue not mentioned at all is the presence of co-mobidities within the target populations. Could rheumatoid arthritis or lupus skew the decision to use or not to use statins for patients with CVD at ages greater than 50 years?<br><br>As a patient, I found one paragraph in particular difficult to follow (page 12, lines 11 to 16) and suggest a more simplified version be provided.<br><br>The article successfully highlights the importance of age and sex stratification and suggests a possible practical framework.

**REVIEWER 2**	Mahmood, Fareena; Public Reviewer. Competing Interest: None
**REVIEW RETURNED**	20-Dec-2022

**GENERAL COMMENTS**	The paper highlights reducing the high risk of cardiovascular disease(CVD) in individuals by preventative interventions such as statin therapy while adopting a more stratified approach using age and sex thresholds within existing algorithms. The paper concludes that overall CVD free life years gained is about 0.16 years which is approximately 2 months only for for those less than 50 years. Treating younger populations at high risk will result in higher treatment costs over prolonged periods but for a patient carer, predictions will enable us to better understand the benefits of the intervention and also motivate the patient to adhere to the therapy. It will be very valuable if the study is extended to additional variables such as lifestyle interventions in younger people and to understand whether this will result in increased CVD free life years compared to the prediction in the current study.

**REVIEWER 3**	Reviewer 3. Competing Interest: None
**REVIEW RETURNED**	20-Dec-2022

**GENERAL COMMENTS**	This a well-conducted and important study on the benefits of age- and sex-specific thresholds for prioritizing preventive treatment for cardiovascular disease. The authors use a large electronic health database to estimate the predictive and clinical benefits associated with stratification of risk thresholds.<br><br>I have no major comments. Below I suggest minor additions to the text that may better explain the methods and contextualize the results.<br><br>Abstract<br>The abstract provides a detailed description of the study.<br><br>Introduction<br>The Introduction clearly sets out the case for age- and sex-stratified risk thresholds.<br><br>Methods<br>The Methods are appropriate to address the paper's objective.<br><br>The decision to drop patients taking statins at baseline is understandable but could lead to selection bias. If there are significant differences between statin users and statin non-users who are eligible for treatment, this may bias estimates of the predictive and clinical benefits of different treatment prioritization strategies. The authors assume the benefits of age- and sex-stratified risk thresholds in the latter group are indicative of these benefits in the wider CVD-free population. They may wish to validate this assumption.<br><br>The way that population public health benefit is quantified is relatively simplistic. The authors assume a consistent 25% CVD risk reduction from statins across all patients (adjusted for subgroup-level adherence). This may ignore predictable

	heterogeneity in treatment effect across patients. Specifically, relative risk reduction from statins is likely determined by baseline cholesterol levels (https://pubmed.ncbi.nlm.nih.gov/26945047/). The authors may wish to adjust for this fact in their analysis, as this modifiable cause of CVD varies across age- and sex-defined subgroups of the UK population. Alternatively, this should be acknowledged as a limitation of the analysis.<br><br>Results<br>The Results section is clearly written and provides all necessary information to readers.<br><br>Discussion<br>The Discussion section describes the results and their implications well. The authors note that there are potential side-effects and cost concerns related to treating more and younger patients with statins. These concerns, alongside the discounting future health and cost outcomes, are addressed in our recent cost-effectiveness analysis of strategies to prioritize preventive statin therapy in the Scottish population (https://pubmed.ncbi.nlm.nih.gov/35249370/). This analysis seems like a relevant point of discussion for the authors, given their statement that "more evidence of cost-effectiveness analyses and net-benefit evaluation in the contemporary UK population is warranted."

**REVIEWER 4**	Reviewer 4. Competing Interest: None
**REVIEW RETURNED**	20-Dec-2022

**GENERAL COMMENTS**	In this study, Zhe Xu and colleagues quantified the application of age- and sex-specific CVD risk thresholds for guiding clinical decisions for statin initiation using both UK and European risk prediction tools within a UK primary care population free of CVD and diabetes, using contemporary data collected between 2004 and 2019. By using the information on 90th percentile of age- and sex-specific risk distributions as an example, lower thresholds were set and more young people (women aged <53 and men aged <47 years) were stratified at high-risk of CVD to initiate statins when using the QRISK2 risk algorithm in comparison to a 10% fixed threshold. The authors concluded that for these groups the age- and sex-specific stratification strategy substantially improved sensitivity (with only a slight decrease in specificity), as well as the discriminatory ability and reclassification. Also, among the younger individuals, the estimated NNS was reduced markedly, while NNT only increased modestly, and overall, there was a small increase in CVD-free life-years gained from statin treatment. As a secondary analysis, for the SCORE2 algorithms similar patterns were found. Although a sex- and age-specific approach is already recommended and used in Europe and beyond, this work appears to be the first to provide quantitative evidence of using such thresholds for allocating statins in the UK population. In my view, this paper covers a relevant clinical topic within cardiovascular primary prevention in general practice. It is well written and contains a series of thoroughly performed analyses.<br><br>I have a few concerns mainly pertaining to well-known, cohort-related forms of bias (1), the validity of some of the assumptions made (2) and the implications for clinical practice (3).

	First, regarding the selection of individuals from the CPRD: all individuals were stratified based on a one-off application of the proposed strategies at a single baseline in time, excluding individuals with statin treatment (at baseline). Analyses then focused on high-risk individuals who had not (yet) received statin treatment. To what extent differed they from the ones who had? Would it be conceivable that this statin-naive population might reflect a population (at least in the years following inception) that has a lower probability of receiving statin treatment, e.g. with poor access to primary care, insufficient motivation to use statins, lack of awareness or treatment inertia by GPs, a preference to improve lifestyle rather than using medication, etc.? The authors describe that medication was initiated in around twenty percent ('drop-ins'), which impresses as rather low, but perhaps it is comparable to other studies from this period? Although little information might be available on the determinants of non-use, it might be useful to contrast the users with non-users for an exploratory analysis on potential differences.<br>Similarly, reporting bias towards cardiovascular risk is likely to lead to missingness not at random (MNAR). For instance, registration of relevant CVD risk factors including smoking, blood pressure, cholesterol and weight/BMI may be skewed towards people with highest values (as a result of the higher propensity to report/register abnormal values relevant for CVD risk assessment, as well as resulting from higher consultation rates of individuals with increased compared to normal risk). Under such circumstances, multiple imputation by chained equations (MICE) may not optimally lead to the desired adjustment, since the risk level status of missing cases is for a large part dependent on a factor that is not recorded for these participants: the risk status itself.(1) As a result, this may affect the estimated relations between risk factors and CVD risk, although previously the QRISK2 was developed and validated within the CPRD dataset, so its impact may overall be small. Nevertheless, earlier researchers in the CPRD database may have studied this potential limitation while studying this topic.(2)<br><br>Second, I think that two of the assumptions underlying the calculation of the public health modelling metrics (Supplementary Method 3) may be somewhat overoptimistic.<br>The first one is on the compliance with allocated statin treatment and states that the proportion of adherence (Pa) was assumed to be 70% for the reference group (women aged 55 to 64 years old). The authors refer to the study by Colantonio et al. (2019), but within the group of patients without CVD and diabetes this percentage may not reach such high levels (although I may have overlooked supplementary files with figures stratified for sex). Furthermore, more recently Talic et al. (2022) conducted a retrospective cohort study using a random sample of 141,062 statin users from the Australian national prescription claims data, and found an average 5-year adherence level of approximately 50%.(3) This is in accordance with a similar study by Toth et al.(4)<br>The third assumption states that the relative risk reduction maintains constant from the initiation to the remaining follow-up years. This assumption is not further substantiated, but I would expect this assumption to be dependent on the first one, where others have shown that adherence rates decline substantially over time.(3-4) But perhaps the authors meant that the RRR was constant, adjusted for adherence rate and independent of age and

	sex (assumption 2)?<br>Since both the level of adherence rate and its potential decline over time can substantially affect the projected overall public health impact (5), the authors might consider performing additional sensitivity analyses, to explore the robustness of their findings.<br><br>Third, I would like to share some concerns on the implications for clinical practice. Although I do agree that there is an important role of age and sex in CVD risk stratification (which is already recommended by the ESC 2021 guideline), their application in daily practice may pose several challenges. For instance, unless GPs are supported by automated EMR algorithms, using a ≥90th-percentile (or other percentile) of the age- and sex-specific risk distributions may be difficult to operationalise, with thresholds shifting across sex and age, as well as across geographical settings and time. This should be an important priority for further research. Another challenge might emerge when overall high risk (e.g. ≥90th-percentile) may come into conflict with cut-off values of individual risk factor values (for cholesterol, but also for others, including blood pressure, or BMI), where these may still be below levels that warrant drug treatment (e.g. SBP below 140 mmHg in very young persons, or BMI below 27 in oldest age groups)(Supplementary Table 4). Finally, as mentioned before, the window of opportunity for statin treatment in this population may be lower, as a result of potential selection effects. The authors may like to reflect on such limitations, either in the manuscript or supplementary files.<br><br>Minor points<br>Introduction<br>P5, line 13: 'Institute' (typo)<br>P6, line 2: consider adding 'estimated fatal or non-fatal 10-year CVD risk' (for clarification; since navigating on CVD mortality only (or CVD morbidity only) were strategies that have been used in the past)<br><br>Methods<br>Since 'no history of statins' may not be similarly associated with either end of the socioeconomic spectrum, it might be informative to explore this determinant (Townsend deprivation score), e.g. in comparison to the statin users that were excluded at baseline (and/or add it to Table 1)<br>P8, line 19: within 'Strategy B' the 90th percentile is chosen 'as an example' (p16, line 16), but in my perception this is not yet stated clearly up until this sentence. Also, it might be useful to further substantiate the choice for p90, to avoid a discussion about arbitrariness similar to the previous choice of a 10% treatment threshold for the younger age groups.<br>P15, line 14: something appears missing here; perhaps it should read: 'until around age 55-60 years'?<br>P36, lines 46-47: perhaps remove 'might result in and'? (or add additional text that might have been lost).<br><br>Results<br>P12, line 13: the CVD incidence rates for the QRISK2 and SCORE2 are 10.4 and 7.3 per 1000 person-years respectively. This impresses as a substantial difference, given the apparently similar operationalisation of end-points (SCORE2 even includes heart failure where for QRISK2 this was not mentioned). Perhaps this requires further clarification (this also refers to the

	interpretation of the differences between Supplementary Figures 2 and 3).<br>P29 (Table 1), line 27: prescription of antihypertensive medication: there is a substantial difference between men (17.9%) and women (28.6%), does this reflect UK prescription rates for primary care? Or might it also give a hint of potential selection effects at baseline (see my earlier point)?<br>P13 Supplement (Suppl. Figure 2): 13,349 CVD end-points (16,7%) were found in the CPRD, outside HES. Did the authors explore whether this may have been the lighter part of the disease spectrum (i.e. TIAs, suspected angina pectoris, etc.). Since this group may contain a relatively high false-positive rate of CVD events (i.e. suspected TIA was not considered an acute indication for hospital referral in the first years of the registration period), it might be worthwhile to perform an additional sensitivity analysis to explore the overall impact on studied stratifications. N.B. Perhaps there are alternative explanations for the 'mismatch' with HES, e.g. hospitals that were outside the data linkage, etc.?<br>P15 Supplement (Suppl. Figure 4): perhaps a y-axis up to 8000 should be sufficient here? A line for x=10% similar to the other figures could be added here. Also, consider choosing a smaller part of x-axis coverage (idem for Suppl. Figures 5,7 and 8).<br>Supplementary Figures 4-9: please add '10-year predicted CVD risk' (or something similar, to emphasize the 10-year projection)<br>P20 Supplement (Suppl. Figure 9): does the slight discontinuity reflect the use of SCORE2 and SCORE-OP respectively? Consider adding this to the legenda.<br><br>Discussion<br>General point: between 2004 and 2019, guideline recommendations have shifted from a separate risk-factor approach to a more integrated, overall risk-guided approach, which may have had consequences for the treatment of dyslipidaemia (e.g. changing from navigating on absolute cholesterol levels to overall CVD risk). It might be worth mentioning changes in usual care (including the introduction of NHS health checks) over time to facilitate interpretation.<br>P21-22 Supplement (Suppl. Figures 10 and 11): perhaps the overall fit of the SCORE2 models is slightly less compared to the QRISK2, since the QRISK2 was originally developed and validated based on (British) CPRD-data? It might be worth commenting on this in the discussion (or in the supplementary files). N.B. Please note that there are two series of Supplementary Figures 9, 10 and 11 (see p20-22 and p23-25).<br>P18, line 22: consider 'dependent' (instead of 'depended')<br>P19, line 8: 'easier' than implementation of lifetime risk? Consider phrasing more tentatively, since little is still known on this subject.<br><br>References: perhaps some recent studies are useful to include, related to assumption 2 in Supplementary Method 3 (also see below).(6)<br><br>References<br>1. Joost R. van Ginkel, Marielle Linting, Ralph C. A. Rippe & Anja van der Voort (2020) Rebutting Existing Misconceptions About Multiple Imputation as a Method for Handling Missing Data, Journal of Personality Assessment, 102:3, 297-308, DOI: 10.1080/00223891.2018.1530680<br>2. McFadden E, Stevens R, Glasziou P, Perera R. Implications of lower risk thresholds for statin treatment in primary prevention:

	analysis of CPRD and simulation modelling of annual cholesterol monitoring. Prev Med. 2015 Jan;70:14-6. doi: 10.1016/j.ypmed.2014.11.004. Epub 2014 Nov 18. PMID: 25445333<br>3. Talic S et al. Switching, Persistence and Adherence to Statin Therapy: a Retrospective Cohort Study Using the Australian National Pharmacy Data. Cardiovasc Drugs Ther . 2022 Oct;36(5):867-877. doi: 10.1007/s10557-021-07199-7. PMID: 34097194<br>4. Toth PP, Granowitz C, Hull M, Anderson A, Philip S. Long-term statin persistence is poor among high-risk patients with dyslipidemia: a real-world administrative claims analysis. Lipids Health Dis. 2019;18(1):175.<br>5. Martin-Ruiz E et al. Systematic Review of the Effect of Adherence to Statin Treatment on Critical Cardiovascular Events and Mortality in Primary Prevention. J Cardiovasc Pharmacol Ther. 2018 May;23(3):200-215. doi: 10.1177/1074248417745357. PMID: 29343082<br>6. Yebyo HG, Aschmann HE, Kaufmann M, Puhan MA. Comparative effectiveness and safety of statins as a class and of specific statins for primary prevention of cardiovascular disease: A systematic review, meta-analysis, and network meta-analysis of randomized trials with 94,283 participants. Am Heart J. 2019 Apr;210:18-28. doi: 10.1016/j.ahj.2018.12.007. Epub 2019 Jan 10. PMID: 30716508

**REVIEWER 5**	Jackson, Rod; University of Auckland. Competing Interest: None
**REVIEW RETURNED**	20-Dec-2022

**GENERAL COMMENTS**	This paper investigates an alternative statin treatment threshold (strategy B) to the current 10% 10-year predicted CVD risk threshold using the QRISK equations recommended by NICE (strategy A). The rationale for the current single threshold across all age groups is based on the evidence that the benefit of treatment is directly proportional to the pre-treatment risk, therefore the current single threshold is in effect a single 'numbers needed to treat to prevent one event' (NNT) threshold. The alternative strategy (B) investigated in this paper changes the threshold to include people above the 90th percentile of risk, in those age groups where fewer than 10% of the population have a CVD risk above 10%. So, it simply involves lowers the CVD risk statin treatment threshold in some younger age groups.<br><br>The main study finding is that for men aged less than 48 years and women aged less than 54 years, strategy B involves lowering the strategy A predicted CVD risk threshold below 10% over 10 years. The main implication of this finding is that under strategy B, more of these younger people will be treated under strategy B than strategy A, which will inevitably lead to more events prevented, although the 'cost' will be an increase in the NNT. The investigators demonstrate this increased NNT (described in Figure 2B and 2D), but surprisingly do not present this information in the Abstract, despite it being the key metric of treatment threshold performance used to determine the current NICE recommendations in the Abstract. Instead, in the Abstract they present three metrics of apparent treatment threshold performance improvement, two of which are not actually measures of improvement and the third is a contentious one.

	The first metric – improved discriminatory ability of strategy A versus B measured with the AUROC-DP – is the inevitable consequence of lowering the treatment threshold from one that includes fewer than 10% of the highest risk people to one that the 10% at highest risk. There will be a point at which the increased sensitivity of a lower threshold will be counter-balanced by reduced specificity, but it will be considerably lower than the 90th centile threshold. It would have been of some interest if the investigators had calculated this lower threshold.<br><br>The second metric – the numbers needed to screen (NNS) to prevent an event – is unfortunately a meaningless metric in the context of this study. This is because not only is it the inevitable consequence of lowering the risk threshold, but the NNS will continue to get smaller as the threshold is lowered, with the best NNS when the threshold is lowered to the point that everyone gets treated. For example, if say 1000 people aged 45 years are screened using QRISK and 50 have a predicted risk greater than 10%, then these 50 people will be prescribed statins (strategy A). If, say, this treatment strategy reduces the number of CVD events among these 50 people by one CVD event compared to not treating them with statins, then the NNS = 1000. However, if the threshold is set to include the top 10% at risk (strategy B) which means 100 of the 1000 people screened will meet the treatment threshold, then the number of people treated inevitably rises and the number of events prevented (the denominator in the NNS calculation) inevitably rises and so the NNS inevitably falls. As stated above, the lowest NNS (i.e. the best) will be observed when everyone is treated, making this a meaningless metric.<br><br>The third metric – gain in CVD-free life-years gained is contentious, because unless one takes into account the different case fatality at different ages and more importantly, unless one considers the fact that most people discount events that are likely to occur far into the future, then, again, treatment will inevitably favour younger people. I recommend that the authors read the paper by S Liew and colleagues (BMJ Open 2012;2: e000728. doi:10.1136/ bmjopen-2011-000728) demonstrating the impact of accounting for case fatality and discounting on the PYLL for people of different ages with the same predicted CVD risk. Of note, this final metric is the only one described by the authors that potentially suggests a benefit of strategy B over strategy A, albeit a contentious one. The reason it is contentious is because there are opposing views as to whether the ‘value’ of preventing future event should discounted or not.<br>Therefore, in my opinion, the paper would benefit from a complete rewrite, with the main focus on the increased cost of strategy B in terms of a higher NNT, but a potential gain in CVD-free life-years (but only if discounting and case fatality are not taken into account).<br><br>I would remove the analyses involving the NNS and the AUROC-DP for the reasons I state above.<br><br>As strategy A and strategy B are identical for men aged 48 and over and for women aged 54 years and over, it is unclear what the point of including them is, particularly up to age 80 years. Perhaps a comparison group aged 50-60 years would be worthwhile for comparing NNTs, but there is nothing to gain by including people

	over 60 years.<br><br>I would also recommend excluding the SCORE-related findings. These findings are not relevant to the current paper as reflected by the fact that they are not even mentioned in the Abstract. They merely make the paper far too long. A comparison between QRISK and SCORE would be an important paper in its own right, but it is not the focus of this paper.<br><br>A couple of minor things:<br>Is there a reason for using QRISK2 rather than QRISK3? If so, it would be useful to mention this. I may have missed it.<br>The supplementary figures are wrongly numbered.<br>The scale on the y axis in Figure 2a and 2c appears to be logarithmic. This should be pointed out in the text and as a footnote in the Table.

VERSION 1 – AUTHOR RESPONSE

Reviewer: 1

Recommendation:

Comments:
Publish – yes
1) This article is complex to read unless the reader has a substantial statistical background. Given this, a little simplification may be of benefit to a wider readership. Integrating the abstract with a version of the text found on page 6, lines 11 to 16 would, I believe, assist the reader. The article proposed a strategy which utilises the top 90th percentiles from age and sex specific risk distributions to illustrate potential advantages of applying age and sex thresholds in CVD risk stratification. In this the article is successful.

Author response: The abstract and manuscript has been substantially revised to benefit wider readership. Regarding to the text originally on page 6, lines 11-16 about the description of ESC guideline, SCORE2, and the age-specific threshold, we have now moved all the results using SCORE2 and corresponding risk threshold strategy to the supplementary file to make the main results easier to follow, as also suggested by other reviewers.

2) From a patient perspective the nagging question relates to how a GP would actually use the findings. Could a simplified age by gender lookup table with the necessary caveats be of assistance in the decision to utilise statins? The life-years gain with statin initiation for men aged 40 years was stated as 0.16 years (page 4, line 1). This observation is not discussed in any detail elsewhere in the article so could be considered an unnecessary observation.

Author response:
a. Table 2 presents the information for a "lookup table" for proposed age- and sex-specific thresholds. The implementation into clinical systems is now mentioned in both the introduction (page #, line #) and discussion (page #, line #).
b. We now explicitly state and discuss the key result of a 0.14-0.16 population average gain in life-years compared with strategy-A (10% single threshold) in the Abstract (page 3, line 19) and in Discussion section (page 17, line 8).

3) While acknowledged by the authors, life-style, diet, ethnicity and medication adherence over time are important factors which could limit the successful outcome at the GP level.

Author response:
Other interventions and medication adherence were not considered in this study, but do remain important. In the Discussion section (page 18 lines 20-22), we do discuss the possible extension of our

methods to other preventive interventions, including lifestyle modification: "*It is also possible to extend the insight of age- and sex-specific thresholds to inform the implementation of other preventive interventions, such as health education, lifestyle modification, and hypertension treatment.*"

We also discussed that beyond age and sex, a potential extension of the risk stratification strategy could also account for ethnicity or other social-economic status (page 17, lines 14-18): "*Risk thresholds could be further specified by ethnicity and other metrics of social-economic status; such stratification may have important implications for the fairness of risk assessments beyond age and sex.*[17,18] *Alternatively, individuals could be stratified by their "potential impact of treatment", which incorporate causal effects of risk factors modification on disease risk and disease-free life years (e.g., the JBS3 Risk Calculator*[19]*).*"

In addition, we have now added another implication of the risk stratification strategy, which is "*to motivate patients to adhere to statin therapy*" in the implications section in the Discussion (page 18, line 8).

4) Adherence rates are briefly mentioned and should be discussed further in terms of impacts on strategies A and B. More discussion of these concerns would benefit the reader.

Author response:
In Supplementary Methods 3, we have considered both **age and sex** when estimating the adherence to statins,[20,21] and the estimated proportion of people adhering to statin treatment ranged from 50% (women aged 40-44) to 90% (men aged 65-70) (Supplementary Table 19).

We agreed that although we have considered age and sex, assuming a constant adherence rate over time may still lead to bias. Therefore, we added this as a limitation in the Discussion section (page 21, lines 4-10) and cited the two reference papers as follows:
"*Furthermore, recent studies have shown that the proportion of people adhering to statins declines over time (e.g., 76% at 6 months versus 51% at 5 years).*[22,23] *Although we considered different adherence rates by age and sex, we assumed they were time-invariant which may result in an overestimation of the long-term performance measures amongst individuals who are more likely to discontinue statin therapy (e.g., men and younger people). In addition, the heterogeneity in other characteristics (e.g., cholesterol levels), may also affect the actual individualised statin benefit.*"

5) An issue not mentioned at all is the presence of co-morbidities within the target populations. Could rheumatoid arthritis or lupus skew the decision to use or not to use statins for patients with CVD at ages greater than 50 years?

Author response:
Common comorbidities such as hypertension, chronic kidney disease, atrial fibrillation, and rheumatoid arthritis are included as risk predictors in the QRISK2 model (see Supplementary Method 1. Whilst the risk score estimation has factored in such co-morbidities characteristics to identify individuals above the thresholds, we have not considered how co-morbidities may impact the clinical decision to initiate statins or other medications. We now state this as a limitation on page 21, lines 13-15 as follows:
"*Third, we have assumed allocation of statins to all people with risk above the set thresholds, and have not incorporated more personalised clinical decisions which may factor in existing co-morbidities and medications.*"

6) As a patient, I found one paragraph in particular difficult to follow (page 12, lines 11 to 16) and suggest a more simplified version be provided.
The article successfully highlights the importance of age and sex stratification and suggests a possible practical framework.

Author response:
We have now simplified this paragraph . We have revised to "*A total of 80,569 incident CVD events were identified during a median follow-up period of 7.8 (5th, 95th percentile: 0.9, 13.4) years (Supplementary Figure 2), with an incidence rate of 10.4 (95% CI: 10.3, 10.5) per 1000 person-years*".
In addition, all results for the SCORE2 model have been moved to supplementary material.

Reviewer: 2

Recommendation:

Comments:
The paper highlights reducing the high risk of cardiovascular disease(CVD) in individuals by preventative interventions such as statin therapy while adopting a more stratified approach using age and sex thresholds within existing algorithms. The paper concludes that overall CVD free life years gained is about 0.16 years which is approximately 2 months only for those less than 50 years. Treating younger populations at high risk will result in higher treatment costs over prolonged periods but for a patient carer, predictions will enable us to better understand the benefits of the intervention and also motivate the patient to adhere to the therapy.

1) It will be very valuable if the study is extended to additional variables such as lifestyle interventions in younger people and to understand whether this will result in increased CVD free life years compared to the prediction in the current study.

Author response:
Other interventions and medication adherence were not considered in this study, but do remain important. In the Discussion section (page 18, lines 20-22), we do discuss the possible extension of our methods to other preventive interventions, including lifestyle modification: "It is also possible to extend the insight of age- and sex-specific thresholds to inform the implementation of other preventive interventions, such as health education, lifestyle modification, and hypertension treatment."

Reviewer: 3

Recommendation:

Comments:
This a well-conducted and important study on the benefits of age- and sex-specific thresholds for prioritizing preventive treatment for cardiovascular disease. The authors use a large electronic health database to estimate the predictive and clinical benefits associated with stratification of risk thresholds.

1) I have no major comments. Below I suggest minor additions to the text that may better explain the methods and contextualize the results.

Abstract
The abstract provides a detailed description of the study.

Introduction
The Introduction clearly sets out the case for age- and sex- stratified risk thresholds.

Methods
The Methods are appropriate to address the paper's objective.
The decision to drop patients taking statins at baseline is understandable but could lead to selection bias. If there are significant differences between statin users and statin non-users who are eligible for treatment, this may bias estimates of the predictive and clinical benefits of different treatment prioritization strategies. The authors assume the benefits of age- and sex- stratified risk thresholds in the latter group are indicative of these benefits in the wider CVD-free population. They may wish to validate this assumption.

Author response:
To address this, we have now added results to compare the baseline characteristics of individuals with and without statin treatment history at baseline (new Supplementary Table 2). The results show that: 1) the proportion of individuals with statin treatment history at baseline is small (80,860 [7.2%] vs 1,046,736 [92.8%]); 2) individuals with statin treatment at baseline were older than those without statins (mean age at baseline of 65 vs 56); 3) for younger individuals with baseline age of 40-60 years, i.e., the main population whose risk category would be different using strategies A and B, the proportion of individuals with statin treatment at baseline is low (Supplementary Table 3), with an overall proportion

of 3.8% amongst people aged 40-60 at baseline. Therefore, the potential risks of selection bias caused by the difference across individuals with and without statin treatment history may be ignored.

We have now added the following text to the Results and Discussion sections:
Results (page 13, lines 6-9): "*Excluded individuals with statin treatment history at baseline (n=80,860) were older (mean age at baseline of 65 [SD = 10] years) in comparison with individuals without statin treatment history at baseline (n=1,046,736) (Supplementary Tables 2 and 3).*"

Discussion (page 20, lines 9-15): "*Moreover, to provide evidence for supporting the decision-making on statin initiation among a statin-naïve population, we excluded individuals with statin treatment at baseline. Potential risks of selection bias caused by the difference across individuals with and without statin treatment history may be negligible, because only a small proportion (3.8%) of individuals aged younger than 60 had statin treatment history at baseline. This would have little impact on the predicted risk distributions, the cut-offs for strategy-B, and the assessments of the comparison between risk stratification strategies for younger individuals.*"

2) The way that population public health benefit Is quantified Is relatively simplistic. The authors assume a consistent 25% CVD risk reduction from statins across all patients (adjusted for subgroup-level adherence). This may ignore predictable heterogeneity in treatment effect across patients. Specifically, relative risk reduction from statins is likely determined by baseline cholesterol levels (https://pubmed.ncbi.nlm.nih.gov/26945047/). The authors may wish to adjust for this fact in their analysis, as this modifiable cause of CVD varies across age- and sex-defined subgroups of the UK population. Alternatively, this should be acknowledged as a limitation of the analysis.

Author response:
Thank you for the reference paper. We acknowledged that assuming a constant effect of statin treatment is one of the limitations of our study in the Discussion section. We now have further added that "*In addition, the heterogeneity in other characteristics (e.g., cholesterol levels), may also affect the actual individualised statin benefit.*[24]" with the reference paper cited in this section (page 21, line 8-10).

3) Results
The Results section is clearly written and provides all necessary information to readers.

Discussion
The Discussion section describes the results and their Implications well. The authors note that there are potential side-effects and cost concerns related to treating more and younger patients with statins. These concerns, alongside the discounting future health and cost outcomes, are addressed in our recent cost-effectiveness analysis of strategies to prioritize preventive statin therapy in the Scottish population (https://pubmed.ncbi.nlm.nih.gov/35249370/). This analysis seems like a relevant point of discussion for the authors, given their statement that "more evidence of cost-effectiveness analyses and net-benefit evaluation in the contemporary UK population is warranted."

Author response:
Thank you for the reference paper.
a. We have now adjusted for the discounting future health problem when calculating the CVD-free life expectancy, per the suggestions from Reviewer 5.
b. We acknowledged that more evidence of cost-effectiveness analyses is still warranted in the Discussion – Implications section (page 18, lines 14-20). We now have cited this paper as follows:
"*Evidence from meta-analyses of clinical trials*[11] *and observational studies*[12] *suggests small absolute excess harm of statins, and microsimulation models in the US and Scottish populations indicate improved cost-effectiveness with lower CVD risk thresholds.*[13–15] *However, evidence is still not consistent across studies.*[16] *Therefore, more research on the use of age- and sex-specific thresholds in cost-effectiveness analyses in different populations with limited health resources is warranted, and subsequent efforts on improving adherence to long-term statin therapy are important.*"

Reviewer: 4

Recommendation:

Comments:
In this study, Zhe Xu and colleagues quantified the application of age- and sex-specific CVD risk thresholds for guiding clinical decisions for statin initiation using both UK and European risk prediction tools within a UK primary care population free of CVD and diabetes, using contemporary data collected between 2004 and 2019. By using the information on 90th percentile of age- and sex-specific risk distributions as an example, lower thresholds were set and more young people (women aged <53 and men aged <47 years) were stratified at high-risk of CVD to initiate statins when using the QRISK2 risk algorithm in comparison to a 10% fixed threshold. The authors concluded that for these groups the age- and sex-specific stratification strategy substantially improved sensitivity (with only a slight decrease in specificity), as well as the discriminatory ability and reclassification. Also, among the younger individuals, the estimated NNS was reduced markedly, while NNT only increased modestly, and overall, there was a small increase in CVD-free life-years gained from statin treatment. As a secondary analysis, for the SCORE2 algorithms similar patterns were found. Although a sex- and age-specific approach is already recommended and used in Europe and beyond, this work appears to be the first to provide quantitative evidence of using such thresholds for allocating statins in the UK population. In my view, this paper covers a relevant clinical topic within cardiovascular primary prevention in general practice. It is well written and contains a series of thoroughly performed analyses.

I have a few concerns mainly pertaining to well-known, cohort-related forms of bias (1), the validity of some of the assumptions made (2) and the implications for clinical practice (3).

1) First, regarding the selection of individuals from the CPRD: all individuals were stratified based on a one-off application of the proposed strategies at a single baseline in time, excluding individuals with statin treatment (at baseline). Analyses then focused on high-risk individuals who had not (yet) received statin treatment. To what extent differed they from the ones who had? Would it be conceivable that this statin-naive population might reflect a population (at least in the years following inception) that has a lower probability of receiving statin treatment, e.g. with poor access to primary care, insufficient motivation to use statins, lack of awareness or treatment inertia by GPs, a preference to improve lifestyle rather than using medication, etc.? The authors describe that medication was initiated in around twenty percent (‘drop-ins’), which impresses as rather low, but perhaps it is comparable to other studies from this period? Although little information might be available on the determinants of non-use, it might be useful to contrast the users with non-users for an exploratory analysis on potential differences.

Author response:
To address this, we have added results to compare the baseline characteristics of individuals with and without statin treatment history at baseline (new Supplementary Table 2). The results show that: 1) the proportion of individuals with statin treatment history at baseline is small (80,860 [7.2%] vs 1,046,736 [92.8%]); 2) individuals with statin treatment at baseline were older than those without statins (mean age at baseline of 65 vs 56); 3) for younger individuals with baseline age of 40-60 years, i.e., the main population whose risk category would be different using strategies A and B, the proportion of individuals with statin treatment at baseline is low (Supplementary Table 3), with an overall proportion of 3.8% amongst people aged 40-60 at baseline. Therefore, the potential risks of selection bias caused by the difference across individuals with and without statin treatment history may be ignored.

We have now added the following text to the Results and Discussion sections:

Results (page 13, lines 6-9): “*Excluded individuals with statin treatment history at baseline (n=80,860) were older (mean age at baseline of 65 [SD = 10] years) in comparison with individuals without statin treatment history at baseline (n=1,046,736) (Supplementary Tables 2 and 3).*”

Discussion (page 20, lines 9-15): “*Moreover, to provide evidence for supporting the decision-making on statin initiation among a statin-naïve population, we excluded individuals with statin treatment at baseline. Potential risks of selection bias caused by the difference across individuals with and without statin treatment history may be negligible, because only a small proportion (3.8%) of individuals aged younger than 60 had statin treatment history at baseline. This would have little impact on the predicted risk distributions, the cut-offs for strategy-B, and the assessments of the comparison between risk stratification strategies for younger individuals.*”

2) Similarly, reporting bias towards cardiovascular risk is likely to lead to missingness not at random (MNAR). For instance, registration of relevant CVD risk factors including smoking, blood pressure, cholesterol and weight/BMI may be skewed towards people with highest values (as a result of the higher propensity to report/register abnormal values relevant for CVD risk assessment, as well as resulting from higher consultation rates of individuals with increased compared to normal risk). Under such circumstances, multiple imputation by chained equations (MICE) may not optimally lead to the desired adjustment, since the risk level status of missing cases is for a large part dependent on a factor that is not recorded for these participants: the risk status itself.(1) As a result, this may affect the estimated relations between risk factors and CVD risk, although previously the QRISK2 was developed and validated within the CPRD dataset, so its impact may overall be small. Nevertheless, earlier researchers in the CPRD database may have studied this potential limitation while studying this topic.(2)

Author response:
The MAR assumption is often assumed for missing values in electronic health records, due to the extensive observed data (such as age, sex, ethnicity, deprivation, co-morbidities) available which can help make the MAR assumption more feasible.[25] We conducted MICE using all the risk predictors in the QRISK2 model, so that imputed values for, say, blood pressure, accounted for the persons age, sex, ethnicity, CVD outcome, co-morbidities, postcode and all other factors in the QRISK2 model. We accept that the possibility of some missing values being MNAR remains, especially amongst people who do not engage with healthcare for reasons that are difficult to measure, however, we expect the impact of this to be small, given the large sample size and representativeness of the population.

3) Second, I think that two of the assumptions underlying the calculation of the public health modelling metrics (Supplementary Method 3) may be somewhat overoptimistic.
The first one Is on the compliance with allocated statin treatment and states that the proportion of adherence (Pa) was assumed to be 70% for the reference group (women aged 55 to 64 years old). The authors refer to the study by Colantonio et al. (2019), but within the group of patients without CVD and diabetes this percentage may not reach such high levels (although I may have overlooked supplementary files with figures stratified for sex). Furthermore, more recently Talic et al. (2022) conducted a retrospective cohort study using a random sample of 141,062 statin users from the Australian national prescription claims data, and found an average 5-year adherence level of approximately 50%.(3) This is in accordance with a similar study by Toth et al.(4)
The third assumption states that the relative risk reduction maintains constant from the initiation to the remaining follow-up years. This assumption is not further substantiated, but I would expect this assumption to be dependent on the first one, where others have shown that adherence rates decline substantially over time.(3-4) But perhaps the authors meant that the RRR was constant, adjusted for adherence rate and independent of age and sex (assumption 2)?
Since both the level of adherence rate and its potential decline over time can substantially affect the projected overall public health impact (5), the authors might consider performing additional sensitivity analyses, to explore the robustness of their findings.

Author response:

a. In Supplementary Methods 3, we have considered both **age and sex** when estimating the adherence to statins,[20,21] and the estimated proportion of people adhering to statin treatment ranged from 50% (women aged 40-44) to 90% (men aged 65-70) (Supplementary Table 19).
We agreed that although we have considered age and sex, assuming a constant adherence rate over time may still lead to bias. Therefore, we added this as a limitation in the Discussion section (page 21, lines 4-10) and cited the 2 reference papers as follows:
"*Furthermore, recent studies have shown that the proportion of people adhering to statins declines time (e.g., 76% at 6 months versus 51% at 5 years).*[22,23] *Although we considered different adherence rates by age and sex, we assumed they were time-invariant which may result in an overestimation of the long-term performance measures amongst individuals who are more likely to discontinue statin therapy (e.g., men and younger people). In addition, the heterogeneity in other characteristics (e.g., cholesterol levels), may also affect the actual individualised statin benefit.*"

b. We agreed that the assumption of constant relative risk reduction over time may affect the results, therefore we have now added this as a limitation in the Discussion section as follows (page 20, lines 21-

23; page 21, lines 1-4): "*First, in our study we assumed a constant effect of statins* *and age- and sex-specific statin adherence rates. Notably, trial-based meta-analyses suggest the statin effect is fairly independent of age and sex*[26,27] *but increases with treatment duration,*[26,28] *which may lead to an overestimation of performance measures amongst individuals with shorter statin treatment duration over their lifespans (i.e., older individuals), or an underestimation of performance measures amongst younger individuals who could benefit from statins for a longer duration.*"

4) Third, I would like to share some share some concerns on the implications for clinical practice. Although I do agree that there is an important role of age and sex in CVD risk stratification (which is already recommended by the ESC 2021 guideline), their application in daily practice may pose several challenges. For instance, unless GPs are supported by automated EMR algorithms, using a ≥90th-percentile (or other percentile) of the age- and sex-specific risk distributions may be difficult to operationalise, with thresholds shifting across sex and age, as well as across geographical settings and time. This should be an important priority for further research. Another challenge might emerge when overall high risk (e.g. ≥90th-percentile) may come into conflict with cut-off values of individual risk factor values (for cholesterol, but also for others, including blood pressure, or BMI), where these may still be below levels that warrant drug treatment (e.g. SBP below 140 mmHg in very young persons, or BMI below 27 in oldest age groups)(Supplementary Table 4). Finally, as mentioned before, the window of opportunity for statin treatment in this population may be lower, as a result of potential selection effects. The authors may like to reflect on such limitations, either in the manuscript or supplementary files.

Author response:
a. About application in daily practice, although it would require additional features for operationalisation, this would still be practical. If the electronic health care system is able to calculate the QRISK2 then it would be simple for the system to also output the age and sex based threshold alongside that or for the output of the result to include a figure that the GP could read off. If ethnicity or other risk factors were included in the future, the system will be able to calculate the CVD risk and summarize by different factors.

We now have added the following text in the implications section of the Discussion (page 18, lines 9-11):
"With the steady increases of CVD risk scores algorithms into electronic health care systems,[29] *further incorporating age- and sex-specific thresholds to stratify high-risk individuals is a relatively straightforward extension to implement."*

b. For the conflict with cut-off values of individual risk factors, this may not be a big problem. Apart from very high levels of cholesterol, prescribing guidance for statins is based on CVD risk, not on absolute levels of cholesterol. Guidance for hypertension treatment also means that CVD risk is only relevant if the blood pressure is above the threshold. Having a raised CVD on its own does not trigger treatment for blood pressure.

Minor points
5) Introduction
P5, line 13: 'Institute' (typo)

Author response:
This has been corrected.

6) P6, line 2: consider adding 'estimated fatal or non-fatal 10-year CVD risk' (for clarification; since navigating on CVD mortality only (or CVD morbidity only) were strategies that have been used in the past)

Author response:
This has been added.

7) Methods
Since 'no history of statins' may not be similarly associated with either end of the socioeconomic spectrum, it might be informative to explore this determinant (Townsend deprivation score), e.g. in comparison to the statin users that were excluded at baseline (and/or add it to Table 1)

Author response:
To address this, we have now added results to compare the baseline characteristics of individuals with and without statin treatment history at baseline (new Supplementary Table 2). The results show that: 1) the proportion of individuals with statin treatment history at baseline is small (80,860 [7.2%] vs 1,046,736 [92.8%]); 2) individuals with statin treatment at baseline were older than those without statins (mean age at baseline of 65 vs 56); 3) for younger individuals with baseline age of 40-60 years, i.e., the main population whose risk category would be different using strategies A and B, the proportion of individuals with statin treatment at baseline is low (Supplementary Table 3), with an overall proportion of 3.8% amongst people aged 40-60 at baseline. Therefore, the potential risks of selection bias caused by the difference across individuals with and without statin treatment history may be ignored.

We have now added the following text to the Results and Discussion sections:
Results (page 13, lines 6-9): "*Excluded individuals with statin treatment history at baseline (n=80,860) were older (mean age at baseline of 65 [SD = 10] years) in comparison with individuals without statin treatment history at baseline (n=1,046,736) (Supplementary Tables 2 and 3).*"

Discussion (page 20, lines 9-15): "*Moreover, to provide evidence for supporting the decision-making on statin initiation among a statin-naïve population, we excluded individuals with statin treatment at baseline. Potential risks of selection bias caused by the difference across individuals with and without statin treatment history may be negligible, because only a small proportion (3.8%) of individuals aged younger than 60 had statin treatment history at baseline. This would have little impact on the predicted risk distributions, the cut-offs for strategy-B, and the assessments of the comparison between risk stratification strategies for younger individuals.*"

8) P8, line 19: within 'Strategy B' the 90th percentile is chosen 'as an example' (p16, line 16), but in my perception this is not yet stated clearly up until this sentence. Also, it might be useful to further substantiate the choice for p90, to avoid a discussion about arbitrariness similar to the previous choice of a 10% treatment threshold for the younger age groups.

Author response:
We now have added the following in the Methods section (page 9, lines 11-13) when describing strategy B:
"The 90th percentile was selected as an example to illustrate the potential results of applying age- and sex-specific thresholds in CVD risk stratification, with the consideration that this would be a pragmatic and implementable strategy."

9) P15, line 14: something appears missing here; perhaps it should read: 'until around age 55-60 years'?

Author response:
Thank you for pointing this out. It should be added as "*until around age 55-60 years*". All results based on SCORE2 have now been removed per the suggestions from other reviews and editors.

10) P36, lines 46-47: perhaps remove 'might result in and'? (or add additional text that might have been lost).

Author response:
We have removed "*might result in and*".

11) Results
P12, line 13: the CVD incidence rates for the QRISK2 and SCORE2 are 10.4 and 7.3 per 1000 person-years respectively. This impresses as a substantial difference, given the apparently similar operationalisation of end-points (SCORE2 even includes heart failure where for QRISK2 this was not mentioned). Perhaps this requires further clarification (this also refers to the interpretation of the differences between Supplementary Figures 2 and 3).

Author response:
The SCORE2 definition for CVD includes heart failure but not transient ischemic attack or non-fatal angina. In contrast, the QRISK2 definition for CVD includes transient ischemic attack and angina but

not heart failure. These definitions explain the lower IR of SCORE2-CVD compared with QRISK2-CVD. However, we did not further add more clarification on this because all results based on SCORE2 have now been moved to supplementary material per the suggestions from other reviews and editors.

12) P29 (Table 1), line 27: prescription of antihypertensive medication: there is a substantial difference between men (17.9%) and women (28.6%), does this reflect UK prescription rates for primary care? Or might it also give a hint of potential selection effects at baseline (see my earlier point)?

Author response:
Previous research reported that the prevalence of primary care patients with antihypertensive drug prescriptions was 21.9% in 2018.[30] Among those with a first-ever antihypertensive drug between 1988 and 2018, women were more likely to have antihypertensive drug prescription (44.6% were men and 55.4% were women). These are inconsistent with our findings. However, the degree of difference among men and women in our study population is slightly larger than the previous research, and this may be related to the fact that we excluded people with previous CVD (and this is because the risk prediction is to estimate the risk of future incident CVD among those with no CVD history). There may be some underlying heterogeneity of the relationship between sex, hypertension treatment, and CVD history which need further research, but is beyond the research question of our study.

13) P13 Supplement (Suppl. Figure 2): 13,349 CVD end-points (16,7%) were found in the CPRD, outside HES. Did the authors explore whether this may have been the lighter part of the disease spectrum (i.e. TIAs, suspected angina pectoris, etc.). Since this group may contain a relatively high false-positive rate of CVD events (i.e. suspected TIA was not considered an acute indication for hospital referral in the first years of the registration period), it might be worthwhile to perform an additional sensitivity analysis to explore the overall impact on studied stratifications. N.B. Perhaps there are alternative explanations for the 'mismatch' with HES, e.g. hospitals that were outside the data linkage, etc.?

Author response:
Unfortunately we did not perform additional sensitivity analyses to explore the CVD disease spectrum in CPRD and HES, mainly because if not using a comprehensive linked data from primary care, hospital admission, and death certificates, we may get biased estimates of the incident events.[31] The discrepancy of recorded CVD events in CPRD, HES, and mortality records in our study are in line with previous research on the missingness of CVD events recorded in each data source. [31,32] It has been indicated that each data source missed a substantial proportion of events. Therefore, it is important to identify disease diagnoses from the combination of the three databases.

14) P15 Supplement (Suppl. Figure 4): perhaps a y-axis up to 8000 should be sufficient here? A line for x=10% similar to the other figures could be added here. Also, consider choosing a smaller part of x-axis coverage (idem for Suppl. Figures 5,7 and 8).

Author response:
We set the y-axis up to 140000 in the updated figure (new Supp Figure 3) to make the QRISK2 and SCORE2 figures in the same scale. As suggested, we now have changed the y-axis scale to a smaller upper limit of 10000. A line for x=10% has been added as well. For the x-axis coverage, because the maximum value of predicted risk using QRISK2 is more than 80%, we kept the current scale of 0-100%. Figures for SCORE2 results have also been updated accordingly.

15) Supplementary Figures 4-9: please add '10-year predicted CVD risk' (or something similar, to emphasize the 10-year projection)

Author response: We have added as "*predicted 10-year cardiovascular disease risk*" in the titles and/or annotations in the updated supplementary figures and tables, to make it clearer.

16) P20 Supplement (Suppl. Figure 9): does the slight discontinuity reflect the use of SCORE2 and SCORE-OP respectively? Consider adding this to the legend.

Author response: That is correct. We have added this in the annotation of the figure (now Supp. Figure 16) as "*The slight discontinuity of the distribution curve reflects the use of two different sets of parameters from SCORE2 algorithms (SCORE2 for people under age 70 and SCORE2-OP for those aged over 70 years).*"

17) Discussion
General point: between 2004 and 2019, guideline recommendations have shifted from a separate risk-factor approach to a more integrated, overall risk-guided approach, which may have had consequences for the treatment of dyslipidaemia (e.g. changing from navigating on absolute cholesterol levels to overall CVD risk). It might be worth mentioning changes in usual care (including the introduction of NHS health checks) over time to facilitate interpretation.

Author response:
Thank you for the suggestion. We have now added this point in the Discussion (page 18, line 22; page 19, lines 1-2) as follows: "*Such an extension is of importance for targeting overall CVD risk reduction as opposed to targeting a reduction in cholesterol levels only, as has been recently remphasised in CVD prevention guidelines.*.[33]"

18) P21-22 Supplement (Suppl. Figures 10 and 11): perhaps the overall fit of the SCORE2 models is slightly less compared to the QRISK2, since the QRISK2 was originally developed and validated based on (British) CPRD-data? It might be worth commenting on this in the discussion (or in the supplementary files). N.B. Please note that there are two series of Supplementary Figures 9, 10 and 11 (see p20-22 and p23-25).

Author response:
Yes, we also think that the QRISK2 performs better in calibration compared with SCORE2 in our study population, mainly because the QRISK2 was originally developed for the UK population in the general practice setting using the QRESEARCH database-a primary care electronic database. We did not further add more discussion point on this because all results based on SCORE2 have now been moved to the supplementary file per the suggestions from other reviews and editors.

The serial numbers and their references in the main text for all supplementary figures have been updated.

19) P18, line 22: consider 'dependent' (instead of 'depended')

Author response: This has been corrected.

20) P19, line 8: 'easier' than implementation of lifetime risk? Consider phrasing more tentatively, since little is still known on this subject.

Author response:
We have revised this to "*Likewise, age-specific thresholds in combination with existing recommended 10-year CVD risk models, are likely to be easier to be implemented in practice compared with new models for lifetime risk*". (page 19, lines 10-12)

21) References: perhaps some recent studies are useful to include, related to assumption 2 in Supplementary Method 3 (also see below).(6)

References
1. Joost R. van Ginkel, Marielle Linting, Ralph C. A. Rippe & Anja van der Voort (2020) Rebutting Existing Misconceptions About Multiple Imputation as a Method for Handling Missing Data, Journal of Personality Assessment, 102:3, 297-308, DOI: 10.1080/ 00223891.2018.1530680
2. McFadden E, Stevens R, Glasziou P, Perera R. Implications of lower risk thresholds for statin treatment in primary prevention: analysis of CPRD and simulation modelling of annual cholesterol monitoring. Prev Med. 2015 Jan;70:14-6. Doi: 10.1016/j.ypmed.2014.11.004. Epub 2014 Nov 18. PMID: 25445333

3. Talic S et al. Switching, Persistence and Adherence to Statin Therapy: a Retrospective Cohort Study Using the Australian National Pharmacy Data. Cardiovasc Drugs Ther . 2022 Oct;36(5):867-877. Doi: 10.1007/s10557-021-07199-7. PMID: 34097194
4. Toth PP, Granowitz C, Hull M, Anderson A, Philip S. Long-term statin persistence is poor among high-risk patients with dyslipidemia: a real-world administrative claims analysis. Lipids Health Dis. 2019;18(1):175.
5. Martin-Ruiz E et al. Systematic Review of the Effect of Adherence to Statin Treatment on Critical Cardiovascular Events and Mortality in Primary Prevention. J Cardiovasc Pharmacol Ther. 2018 May;23(3):200-215. Doi: 10.1177/1074248417745357. PMID: 29343082
6. Yebyo HG, Aschmann HE, Kaufmann M, Puhan MA. Comparative effectiveness and safety of statins as a class and of specific statins for primary prevention of cardiovascular disease: A systematic review, meta-analysis, and network meta-analysis of randomized trials with 94,283 participants. Am Heart J. 2019 Apr;210:18-28. Doi: 10.1016/j.ahj.2018.12.007. Epub 2019 Jan 10. PMID: 30716508

Author response:
Thanks for the references. We added the references related to assumption 2, i.e., the adherence to statin therapy, in Supplementary Methods 4 and limitation section in the Discussion (page 20, lines 22-23; page 21, lines 1-10).

Reviewer: 5

Recommendation:

Comments:
My review

This paper investigates an alternative statin treatment threshold (strategy B) to the current 10% 10-year predicted CVD risk threshold using the QRISK equations recommended by NICE (strategy A). The rationale for the current single threshold across all age groups is based on the evidence that the benefit of treatment is directly proportional to the pre-treatment risk, therefore the current single threshold is in effect a single 'numbers needed to treat to prevent one event' (NNT) threshold. The alternative strategy (B) investigated in this paper changes the threshold to include people above the 90th percentile of risk, in those age groups where fewer than 10% of the population have a CVD risk above 10%. So, it simply involves lowers the CVD risk statin treatment threshold in some younger age groups.

1) The main study finding is that for men aged less than 48 years and women aged less than 54 years, strategy B involves lowering the strategy A predicted CVD risk threshold below 10% over 10 years. The main implication of this finding is that under strategy B, more of these younger people will be treated under strategy B than strategy A, which will inevitably lead to more events prevented, although the 'cost' will be an increase in the NNT. The investigators demonstrate this increased NNT (described in Figure 2B and 2D), but surprisingly do not present this information in the Abstract, despite it being the key metric of treatment threshold performance used to determine the current NICE recommendations in the Abstract.

Author response:
We have now revised the abstract substantially and only reported on the NNT results, and the gained CVD-free life-years with further adjustment for time preference as suggested by the reviewer

2) Instead, in the Abstract they present three metrics of apparent treatment threshold performance improvement, two of which are not actually measures of improvement and the third is a contentious one. The first metric – improved discriminatory ability of strategy A versus B measured with the AUROC-DP – is the inevitable consequence of lowering the treatment threshold from one that includes fewer than 10% of the highest risk people to one that the 10% at highest risk. There will be a point at which the increased sensitivity of a lower threshold will be counter-balanced by reduced specificity, but it will be considerably lower than the 90th centile threshold. It would have been of some interest if the investigators had calculated this lower threshold.

Author response:

We thank the reviewer for pointing out this significant problem. We agree that there will be a compromise between the increase in sensitivity but reduction in specificity when lowering the threshold. Here we calculated the sensitivity, specificity, and AUROC-dp (all accounted for censoring) at different cut-offs, with the subgroup of men with baseline age at 40 as an example, to illustrate this.
In the following table, when lowering the threshold from 10% to 4.97% (the cut-off at the 90th percentile of the risk distribution in this sub population), to 1.27% (the cut-off at the 10th percentile of the risk distribution in this sub population), sensitivity increased from 5.91% to 26.42%, and to 98.08%, respectively; specificity reduced from 98.71% to 90.45%, to 10.22%, respectively. However, the AUROC-dp reached the maximum value of 0.630 at the cut-off at the 70th percentile, i.e., 3.24%, which demonstrates when incorporating both sensitivity and specificity, this cut-off would be the counter-balanced point. However, considering that using such a cut-off would result in stratifying 30% of people at high-risk in this population, this would not be pragmatic for such a young-aged group. We now have added the following in the Methods section when describing strategy B for choosing the 90th percentile: "*We chose the 90th percentile as an example to illustrate the potential effects of applying age- and sex-specific thresholds in CVD risk stratification, with the consideration that this would be a pragmatic and implementable strategy*." (page XX,

Stratification	10-year predicted risk cut-off	% of the population	AUROC-dp	Sensitivity	Specificity
1 – Fixed threshold	10%	1.4%	0.522	5.91%	98.71%
2 – Cut-off at the 90th percentile	4.97%	10%	0.579	26.42%	90.45%
3 – Cut-off at the 85th percentile	4.27%	15%	0.603	35.82%	85.57%
4 – Cut-off at the 80th percentile	3.83%	20%	0.614	43.07%	80.63%
5 – Cut-off at the 75th percentile	3.51%	25%	0.627	50.92%	75.71%
6 – Cut-off at the 70th percentile	3.24%	30%	**0.630**	56.95%	70.74%
7 – Cut-off at the 65th percentile	3.02%	35%	0.627	61.37%	65.72%
8 – Cut-off at the 60th percentile	2.82%	40%	0.622	65.35%	60.69%
9 – Cut-off at the 55th percentile	2.64%	45%	0.620	69.84%	55.67%
10 – Cut-off at the 50th percentile	2.47%	50%	0.617	74.47%	50.66%
11 – Cut-off at the 45th percentile	2.32%	55%	0.614	79.18%	45.65%
12 – Cut-off at the 40th percentile	2.16%	60%	0.604	82.48%	40.61%
13 – Cut-off at the 35th percentile	2.02%	65%	0.599	86.16%	35.57%
14 – Cut-off at the 30th percentile	1.87%	70%	0.588	88.69%	30.50%
15 – Cut-off at the 25th percentile	1.72%	75%	0.580	91.79%	25.45%
16 – Cut-off at the 20th percentile	1.57%	80%	0.565	93.50%	20.36%
17 – Cut-off at the 15th percentile	1.43%	85%	0.555	96.09%	15.30%
18 – Cut-off at the 10th percentile	1.27%	90%	0.540	98.08%	10.22%

3) The second metric – the numbers needed to screen (NNS) to prevent an event – is unfortunately a

meaningless metric in the context of this study. This is because not only is it the inevitable consequence of lowering the risk threshold, but the NNS will continue to get smaller as the threshold is lowered, with the best NNS when the threshold is lowered to the point that everyone gets treated. For example, if say 1000 people aged 45 years are screened using QRISK and 50 have a predicted risk greater than 10%, then these 50 people will be prescribed statins (strategy A). If, say, this treatment strategy reduces the number of CVD events among these 50 people by one CVD event compared to not treating them with statins, then the NNS = 1000. However, if the threshold is set to include the top 10% at risk (strategy B) which means 100 of the 1000 people screened will meet the treatment threshold, then the number of people treated inevitably rises and the number of events prevented (the denominator in the NNS calculation) inevitably rises and so the NNS inevitably falls. As stated above, the lowest NNS (i.e. the best) will be observed when everyone is treated, making this a meaningless metric.

Author response:
We thank the reviewer for pointing this out. We conducted a sensitivity analysis where we controlled the number of selected individuals to be the same, i.e., using the number estimated from strategy B across all individuals and then identified the corresponding single cut-off as an alternative fixed threshold for strategy A. The results showed that the improvement in NNS reduction remained (Supplementary Table 9). We now have emphasised this sensitivity analysis in the Methods and the Results section as follows:

Methods – Potential public health impact (page 10, lines 15-27):
"*We note that the NNS will always be smaller when the threshold is lowered, and is at a minimum when everyone gets treated. In contrast, the NNT will always increase when the threshold is lowered*."

Methods – Sensitivity analyses (page 11, lines 16-22):
"*Since NNS, NNT, and the population average gain in CVD-free life-years from statin treatment depend on the number of individuals identified as high-risk, to make a fairer comparison across strategies, we further performed sensitivity analyses by ascertaining the same number of high-risk individuals in each strategy. We constrained the number of individuals classified as high-risk of CVD to be the same as the number identified in strategy B among the whole population sample and then identified the corresponding single threshold as an alternative fixed threshold for strategy A. This single threshold was identified as being 9.2% (strategy-A1).*"

Results – Sensitivity analyses (page 16, lines 2-5):
"*When modelling a fixed budget scenario, constraining the total number of individuals stratified as at high-risk of CVD among the whole population sample to be the same across strategies, a single threshold of 9.2% (strategy-A1) was identified to ascertain the same number of high-risk individuals as that from strategy-B among all individuals..*"

4) The third metric – gain in CVD-free life-years gained is contentious, because unless one takes into account the different case fatality at different ages and more importantly, unless one considers the fact that most people discount events that are likely to occur far into the future, then, again, treatment will inevitably favour younger people. I recommend that the authors read the paper by S Liew and colleagues (BMJ Open 2012;2: e000728. Doi:10.1136/ bmjopen-2011-000728) demonstrating the impact of accounting for case fatality and discounting on the PYLL for people of different ages with the same predicted CVD risk. Of note, this final metric is the only one described by the authors that potentially suggests a benefit of strategy B over strategy A, albeit a contentious one. The reason it is contentious is because there are opposing views as to whether the 'value' of preventing future event should discounted or not.
Therefore, in my opinion, the paper would benefit from a complete rewrite, with the main focus on the increased cost of strategy B in terms of a higher NNT, but a potential gain in CVD-free life-years (but only if discounting and case fatality are not taken into account).

Author response:
We thank the reviewer for pointing this out. In our previous calculation, we had accounted for the different case fatality at different ages by estimating the CVD risk and competing risk based on sex-specific lifetables with 1-year age intervals at each age (Supplementary Method 4). For the time preference, we now have adjusted it in the calculation using a rate of 0.03 referring to the methods used in S Liew 2012.[34] After adjustment, although the absolute CVD-free life-years reduced, the gain in CVD-free life-years from statin treatment did not change significantly, and the difference of such gain across

different strategies was similar as before. The maximum increase in the average gain in CVD-free life expectancy was still 0.16 years in men at age 40.

We also added the following text in the Methods section and in Supplementary Methods 5:
"*In addition, the life expectancy was calculated with further adjustment for a time preference rate to account for the increasing lower value that patients currently give to the life years further out into the far-off future…. A time preference rate of 0.03 was used in this study, which values the next year as worth 97% of the previous year..*[34]"

Furthermore, we have now re-written the paper to focus on the trade-off between the NNT and gain in CVD-free life years (see abstract, results and main discussion).

5) I would remove the analyses involving the NNS and the AUROC-DP for the reasons I state above.
Author response:
We have removed the results of these two metrics from the abstract and the main analysis. In addition, we have now conducted a sensitivity analysis which allows a better interpretation of the NNS, and discussed further on the AUROC-DP.

6) As strategy A and strategy B are identical for men aged 48 and over and for women aged 54 years and over, it is unclear what the point of including them is, particularly up to age 80 years. Perhaps a comparison group aged 50-60 years would be worthwhile for comparing NNTs, but there is nothing to gain by including people over 60 years.

I would also recommend excluding the SCORE-related findings. These findings are not relevant to the current paper as reflected by the fact that they are not even mentioned in the Abstract. They merely make the paper far too long. A comparison between QRISK and SCORE would be an important paper in its own right, but it is not the focus of this paper.

Author response:
As suggested, we have revised the main text to focus on the results from QRISK only. We have moved all the results for SCORE2 into supplementary material and have retained because they are highly relevant to the new 2021 European Society of Cardiology (ESC) guidelines. Furthermore, we have removed the results for people over age 70 in the main figures (Figure 1, Figure 2).

A couple of minor things:
7) Is there a reason for using QRISK2 rather than QRISK3? If so, it would be useful to mention this. I may have missed it.

Author response:
In this study, QRISK2 was used for the risk estimation because it is still recommended by the current NICE guideline and currently used in general practice in the UK. We have already emphasised this in the manuscript – page 7, line 7; page 8, lines 12-13.
Note, that although QRISK3 has been more recently developed, it incorporates more risk factors making it more difficult to implement in clinical practice and insert into clinical systems.
e.g.,https://support.ardens.org.uk/support/solutions/articles/31000154307-qrisk3-calculator for Ardens.

8) The supplementary figures are wrongly numbered.

Author response: These have been corrected.

9) The scale on the y axis in Figure 2a and 2c appears to be logarithmic. This should be pointed out in the text and as a footnote in the Table.

Author responseWe have added this in the footnote for NNS in this figure (now Supplementary Figure 10).

References

1. Heagerty PJ, Lumley T, Pepe MS. Time-dependent ROC curves for censored survival data and a diagnostic marker. *Biometrics*. 2000;56(2):337-344. doi:10.1111/j.0006-341x.2000.00337.x

2. Zou KH, O'Malley AJ, Mauri L. Receiver-Operating Characteristic analysis for evaluating diagnostic tests and predictive models. *Circulation*. 2007;115(5):654-657. doi:10.1161/CIRCULATIONAHA.105.594929

3. Muschelli J. ROC and AUC with a binary predictor: a potentially misleading metric. *J Classif*. 2020;37(3):696-708. doi:10.1007/s00357-019-09345-1

4. Newson RB. Comparing the predictive powers of survival models using Harrell's C or Somers' D. *The Stata Journal*. 2010;10(3):339-358. doi:10.1177/1536867X1001000303

5. Collins GS, Altman DG. An independent and external validation of QRISK2 cardiovascular disease risk score: a prospective open cohort study. *BMJ*. 2010;340:c2442. doi:10.1136/bmj.c2442

6. Steyerberg EW, Vickers AJ, Cook NR, et al. Assessing the performance of prediction models: a framework for traditional and novel measures. *Epidemiology*. 2010;21(1):128-138. doi:10.1097/EDE.0b013e3181c30fb2

7. Steyerberg E. *Clinical Prediction Models: A Practical Approach to Development, Validation, and Updating*. Springer-Verlag; 2009.

8. Wolf A, Dedman D, Campbell J, et al. Data resource profile: Clinical Practice Research Datalink (CPRD) Aurum. *International Journal of Epidemiology*. 2019;48(6):1740-1740g. doi:10.1093/ije/dyz034

9. Office for National Statistics, National Records of Scotland, Northern Ireland Statistics and Research Agency, UK Data Service. 2011 UK Townsend Deprivation Scores. Published online 2017. doi:10.5257/CENSUS/AGGREGATE-2011-2

10. Hippisley-Cox J, Coupland C, Brindle P. The performance of seven QPrediction risk scores in an independent external sample of patients from general practice: a validation study. *BMJ Open*. 2014;4(8):e005809. doi:10.1136/bmjopen-2014-005809

11. Taylor F, Huffman MD, Macedo AF, et al. Statins for the primary prevention of cardiovascular disease. *Cochrane Database of Systematic Reviews*. Published online 2013. doi:10.1002/14651858.CD004816.pub5

12. Macedo AF, Taylor FC, Casas JP, Adler A, Prieto-Merino D, Ebrahim S. Unintended effects of statins from observational studies in the general population: systematic review and meta-analysis. *BMC Med*. 2014;12:51. doi:10.1186/1741-7015-12-51

13. Campbell DJ. Can cardiovascular disease guidelines that advise treatment decisions based on absolute risk be improved? *BMC Cardiovascular Disorders*. 2016;16(1):221. doi:10.1186/s12872-016-0396-y

14. Pandya A, Sy S, Cho S, Weinstein MC, Gaziano TA. Cost-effectiveness of 10-Year Risk Thresholds for Initiation of Statin Therapy for Primary Prevention of Cardiovascular Disease. *JAMA*. 2015;314(2):142-150. doi:10.1001/jama.2015.6822

15. Kohli-Lynch CN, Lewsey J, Boyd KA, et al. Beyond 10-Year Risk: A Cost-Effectiveness Analysis of Statins for the Primary Prevention of Cardiovascular Disease. *Circulation*. 2022;145(17):1312-1323. doi:10.1161/CIRCULATIONAHA.121.057631

16. Špacírová Z, Kaptoge S, García-Mochón L, et al. The cost-effectiveness of a uniform versus age-based threshold for one-off screening for prevention of cardiovascular disease. *Eur J Health Econ*. Published online October 14, 2022. doi:10.1007/s10198-022-01533-y

17. Do H, Nandi S, Putzel P, Smyth P, Zhong J. Joint fairness model with applications to risk predictions for under-represented populations. *ArXiv*. Published online May 10, 2021:arXiv:2105.04648v2.

18. Foryciarz A, Pfohl SR, Patel B, Shah N. Evaluating algorithmic fairness in the presence of clinical guidelines: the case of atherosclerotic cardiovascular disease risk estimation. *BMJ Health Care Inform*. 2022;29(1):e100460. doi:10.1136/bmjhci-2021-100460

19. JBS3 Board. Joint British Societies' consensus recommendations for the prevention of cardiovascular disease (JBS3). *Heart*. 2014;100(Suppl 2):ii1-ii67. doi:10.1136/heartjnl-2014-305693

20. Chamnan P, Simmons RK, Khaw KT, Wareham NJ, Griffin SJ. Estimating the population impact of screening strategies for identifying and treating people at high risk of cardiovascular disease: modelling study. *BMJ*. 2010;340:c1693. doi:10.1136/bmj.c1693

21. Colantonio LD, Rosenson RS, Deng L, et al. Adherence to Statin Therapy Among US Adults Between 2007 and 2014. *Journal of the American Heart Association*. 2019;8(1):e010376. doi:10.1161/JAHA.118.010376

22. Talic S, Marquina C, Ofori-Asenso R, et al. Switching, Persistence and Adherence to Statin Therapy: a Retrospective Cohort Study Using the Australian National Pharmacy Data. *Cardiovasc Drugs Ther*. 2022;36(5):867-877. doi:10.1007/s10557-021-07199-7

23. Toth PP, Granowitz C, Hull M, Anderson A, Philip S. Long-term statin persistence is poor among high-risk patients with dyslipidemia: a real-world administrative claims analysis. *Lipids Health Dis*. 2019;18(1):175. doi:10.1186/s12944-019-1099-z

24. Thanassoulis G, Williams K, Altobelli KK, Pencina MJ, Cannon CP, Sniderman AD. Individualized Statin Benefit for Determining Statin Eligibility in the Primary Prevention of Cardiovascular Disease. *Circulation*. 2016;133(16):1574-1581. doi:10.1161/CIRCULATIONAHA.115.018383

25. Petersen I, Welch CA, Nazareth I, et al. Health indicator recording in UK primary care electronic health records: key implications for handling missing data. *Clin Epidemiol*. 2019;11:157-167. doi:10.2147/CLEP.S191437

26. Baigent C, Keech A, Kearney PM, et al. Efficacy and safety of cholesterol-lowering treatment: prospective meta-analysis of data from 90,056 participants in 14 randomised trials of statins. *Lancet*. 2005;366(9493):1267-1278. doi:10.1016/S0140-6736(05)67394-1

27. Brugts JJ, Yetgin T, Hoeks SE, et al. The benefits of statins in people without established cardiovascular disease but with cardiovascular risk factors: meta-analysis of randomised controlled trials. *BMJ*. 2009;338:b2376. doi:10.1136/bmj.b2376

28. Law MR, Wald NJ, Rudnicka AR. Quantifying effect of statins on low density lipoprotein cholesterol, ischaemic heart disease, and stroke: systematic review and meta-analysis. *BMJ*. 2003;326(7404):1423. doi:10.1136/bmj.326.7404.1423

29. Finnikin S, Ryan R, Marshall T. Statin initiations and QRISK2 scoring in UK general practice: a THIN database study. *Br J Gen Pract*. 2017;67(665):e881-e887. doi:10.3399/bjgp17X693485

30. Rouette J, McDonald EG, Schuster T, Brophy JM, Azoulay L. Treatment and prescribing trends of antihypertensive drugs in 2.7 million UK primary care patients over 31 years: a population-based cohort study. *BMJ Open*. 2022;12(6). doi:10.1136/bmjopen-2021-057510

31. Herrett E, Shah AD, Boggon R, et al. Completeness and diagnostic validity of recording acute myocardial infarction events in primary care, hospital care, disease registry, and national mortality records: cohort study. *BMJ*. 2013;346:f2350. doi:10.1136/bmj.f2350

32. Morgan A, Sinnott SJ, Smeeth L, Minassian C, Quint J. Concordance in the recording of stroke across UK primary and secondary care datasets: a population-based cohort study. *BJGP Open*. 5(2):BJGPO.2020.0117. doi:10.3399/BJGPO.2020.0117

33. Leening MJG, Ikram MA. Primary prevention of cardiovascular disease: The past, present, and future of blood pressure- and cholesterol-lowering treatments. *PLoS Med*. 2018;15(3):e1002539. doi:10.1371/journal.pmed.1002539

34. Liew SM, Jackson R, Mant D, Glasziou P. Should identical CVD risks in young and old patients be managed identically? Results from two models. *BMJ Open*. 2012;2(2):e000728. doi:10.1136/bmjopen-2011-000728

VERSION 2 – REVIEW

**REVIEWER**	Riley, Richard; University of Birmingham, Institute of Applied Health Research. Competing Interest: None
**REVIEW RETURNED**	25-Sep-2023

**GENERAL COMMENTS**	This is an interesting and well-written study. The response to reviewers seems sensible and the revision improved. It is thought provoking. I have not read this before, so have a few comments:<br><br>1) The paper is more about lowering the threshold for statins in some age groups, rather than using 90% percentile points for each age group, per se. Therefore, the question should be whether those lower thresholds are clinically sensible – do they provide a better reflection of the benefit to harms ratio that is acceptable in those age groups? This does not seem to be considered – the authors conclude that the extra costs involved in treatment of more younger patients should be accounted for … but this should be part of the threshold decision, shouldn't it?. Hence, it seems backward in the logic – we should chose the thresholds first shouldn't we, based on our accepted harms to benefits ratio based on the threshold(s), and then see if the model is worthwhile at those thresholds? This needs to be discussed.<br>2) Following this, it makes more sense to me to provide the clinical utility assessments for the two strategies in terms of the net benefit and decision curves. Which has larger net benefit at the thresholds of interest? I am referring to applying the work of Vickers on net benefit https://www.bmj.com/content/352/bmj.i6 - surely this is more relevant than the adapted AUROC curve?<br>3) What is an 'adapted' AUROC curve? Simply that it accounted for censoring or changing the threshold at different age? How do we interpret these in real terms?<br>4) Worth making it clear that all the performance measures (including NNS and NNT) focused on the 10 year time-point for prediction performance.<br>5) For the NNS and NNT, I assume the whole distribution of predictions was used – as obviously each individual has their own risk, and this must be accounted for when deriving the NNT and NNS. (That is, it is not a binary thing, with one risk below and one risk above the threshold, but rather a continuum of risks). Please clarify.

	6) The 'calibration (visually assessing the agreement of observed risk and predicted risk by deciles of predicted risk' – I think it is more correct to say tenths of predicted risk.<br>7) The calibration plot does not provide 95% CIs around each point (perhaps because the data are so big?) – was the competing event of death accounted for? This is particular important in the older age groups. Are we assessing calibration in a world where no-one can die (deaths are censored) or in a real world where CVD may not occur because death from other causes happens first?<br>8) Ideally calibration curves should be added to the calibration plots (e.g. using pseudo observations), but this is a minor point and not essential for this paper.<br><br>I hope these comments are constructive for the authors, in the context of an interesting piece of work that would make a good addition to BMC Medicine

VERSION 2 – AUTHOR RESPONSE

Reviewer: 1
Prof. Richard Riley, University of Birmingham
Comments to the Author

This is an interesting and well-written study. The response to reviewers seems sensible and the revision improved. It is thought provoking. I have not read this before, so have a few comments:

We thank Prof Riley for his insightful review of the manuscript and encouraging comment. By addressing his comments, we believe we have improved the manuscript, especially with the addition of the Net Benefit estimates. Please see the revised manuscript attached and our responses to each of the specific comments below.
1) The paper is more about lowering the threshold for statins in some age groups, rather than using 90% percentile points for each age group, per se. Therefore, the question should be whether those lower thresholds are clinically sensible – do they provide a better reflection of the benefit to harms ratio that is acceptable in those age groups? This does not seem to be considered – the authors conclude that the extra costs involved in treatment of more younger patients should be accounted for … but this should be part of the threshold decision, shouldn't it?. Hence, it seems backward in the logic – we should chose the thresholds first shouldn't we, based on our accepted harms to benefits ratio based on the threshold(s), and then see if the model is worthwhile at those thresholds? This needs to be discussed.

Response: Done. We have clarified our study aims, which are to enhance the quantative evidence on the clinical benefits and harms and provide an implementable framework for incorporating age- and sex-specific risk distributions into risk thresholds, with the following changes to the text:

Page 7, Lines 5-7: "*Limited quantitative analysis exists for establishing and assessing the clinical benefits and harms of age- and sex-specific CVD risk thresholds, with a gap in evidence for frameworks which can be adapted and implemented across populations*".
Page 7, Lines 8-13*: "We aim to enhance quantitative evidence and provide a framework for incorporating age- and sex-specific risk distributions into decision-making for statin initiation in primary CVD prevention. Our study utilises a large UK primary care electronic health records database to assess the potential clinical benefits and harms of augmenting recommended 10-year CVD risk prediction tools (i.e., QRISK2*[1] *used in the UK and SCORE2*[2,3] *used across Europe) with thresholds based on the percentiles of risk distributions in the population by age and sex."*

In the **Methods** section (Page 9, Lines 16-19) we now state: *"The 90th percentile was selected as an example to illustrate the potential results of applying age- and sex-specific thresholds in CVD risk stratification. We applied this to lower the thresholds at younger ages rather than to increase the thresholds at older ages, with the consideration that this would be a pragmatic, acceptable and implementable strategy.*"

We acknowledge there are different ways to select the thresholds, and we have extended our discussion on this (Page18, Lines 11-22):

"*Extensions and alternative approaches merit consideration. For example, other potential strategies are ones that achieve equity in sensitivity, false negative rates (FNR) (e.g., a fixed 5% FNR),*[4] *or net benefit* [5] *across different ages for men and women. It is noteworthy that in our study the risk thresholds equal to 90th percentiles of age- and sex-specific risk distributions resulted in approximately equal estimates across younger ages and sex for sensitivity, specificity and net benefit. Risk thresholds could be further specified by ethnicity and other metrics of social-economic status; such stratification may have important implications for the fairness of risk assessments beyond age and sex.*[6,7] *Alternatively, individuals could be stratified by their "potential impact of treatment", which incorporate causal effects of risk factors modification on disease risk and disease-free life years (e.g., the JBS3 Risk Calculator*[8]*). Regardless, we highlight the importance of ensuring changes to thresholds align with a clinically sensible balance between benefits and harms."*

2) Following this, it makes more sense to me to provide the clinical utility assessments for the two strategies in terms of the net benefit and decision curves. Which has larger net benefit at the thresholds of interest? I am referring to applying the work of Vickers on net benefit https://www.bmj.com/content/352/bmj.i6 - surely this is more relevant than the adapted AUROC curve?

Response: Done. In addition to the adapted AUROC, we estimated the NNS, NNT, and gain of CVD-free life expectancy to provide evidence on the clinical benefits and harms of the risk stratification strategies, which we believe are more relevant than the AUROC. We agree that net benefit is also a useful assessment and we have now added this to the manuscript based on the reference paper by Vickers [5] and with the extension of accounting for censoring.[9]

Instead of plotting the decision curves for a range of thresholds for each age (which would be difficult to plot for each age year 40-70), we made an adaptation to present the net benefit by age for our two proposed risk stratification strategies/thresholds (see new **Figure 1** as a main figure for QRISK2 estimation, and **Supplementary Figure 21** for SCORE2 estimation). The results show that for men aged 40-47 years and for women aged 40-54 years, the net benefit was constant and higher for strategy-B compared with strategy-A, strengthening our conclusion.

The description of the methods has been added to the main **Methods** section (Page 10, Lines 17-22) as follows:
*Net benefit was estimated to assess the clinical value of different risk stratification strategies and their clinical consequences.[38, 39] Net benefit represents the difference between the true positive rate and false positive rate weighted by the odds of the selected threshold for being at high risk, with higher values indicating greater net benefit. Sensitivity, specificity, AUROC-dp, and net benefit were all calculated accounting for censoring (detailed methods described in **Supplementary Methods 3** and **4**).*

and **Supplementary Methods 4**

"*Net benefit was calculated using the following equation at each age group:*
Net benefit = True positive rate – False positive rate × $\left(\frac{P}{1-P}\right)$
where N is the total number of the population at each age group, P is the threshold probability to define when the individual is at high risk of developing CVD (i.e., the risk thresholds under each stratification strategy).[5] *Accounting for time-to-event data, the true positive rate is given by* $[1-(S(t)|\ x = 1)]\times P(x = 1)$ *and the false positive rate is given by* $(S(t)|\ x = 1)\times P(x = 1)$*, where x = 1 represents that the individual had a predicted risk greater than the threshold probability P and x = 0 otherwise; S(t) is the Kaplan-Meier survival probability at the chosen time t (which is 10 years in our calculation).*[9] *One assumption of the method is that the mechanism of censoring is independent from the predictors used in the risk prediction model.[9]"*

and the results have been added in the **Results** section (Page 16, Lines 3-6):

*"For men aged 40-47 years and for women aged 40-54 years, the net benefit was approximately constant and equal across age and sex, and higher for strategy-B compared with strategy-A (**Figure 1**).*

*For example, the net benefit was 0.24 for strategy-B versus 0.10 for strategy-A for women at age 50 (**Figure 1**).”*

And mentioned in the discussion (Page 18, Lines 11-16):
“For example, other potential strategies are ones that achieve equity in sensitivity, false negative rates (FNR) (e.g., a fixed 5% FNR),[4] *or net benefit* [5] *across different ages for men and women. It is noteworthy that in our study the risk thresholds equal to 90th percentiles of age- and sex-specific risk distributions resulted in approximately equal estimates across younger age and sex for sensitivity, specificity and net benefit.”*

3) What is an ‘adapted’ AUROC curve? Simply that it accounted for censoring or changing the threshold at different age? How do we interpret these in real terms?

Response: Done. The adapted Area Under Receiver Operating Characteristic curve for dichotomised predictions (AUROC-dp) account for both censoring and the predicted risk dichotomised as above/below the threshold. This was calculated for the overall sample (**Supplementary Tables 6, 15**), and for individuals grouped by age (**Supplementary Tables 8, 17,** and **Supplementary Figures 9, 20**). We have now added more details in the methods section (Page 10, Lines 12-17) describing this measure:

“The AUROC-dp measures the ability to discriminate between individuals who do and who do not have a CVD event according to the combined risk prediction model and the stratification rule. As a measure of discrimination, the AUROC-dp generally takes values from 0.5 (representing discriminative ability equal to chance alone) and 1 (when the risk prediction model and stratification strategy perfectly separates individuals who do and who do not later experience a CVD event). [10–12]”

Further information is provided in **Supplementary Methods 3**.

4) Worth making it clear that all the performance measures (including NNS and NNT) focused on the 10 year time-point for prediction performance.

Response: Done. We have added the following text to the **Methods** section (Page 11, Line 4): *“We quantified the public health impact of the combined risk prediction model and the stratification rule by the numbers needed to screen (NNS) and the numbers needed to treat (NNT) to prevent one new CVD* event *in 10 years**,** under the assumption that statin treatment is allocated to high-risk individuals and reduces CVD risk.”*

This is also described in detail in the **Supplementary Methods 5**.

5) For the NNS and NNT, I assume the whole distribution of predictions was used – as obviously each individual has their own risk, and this must be accounted for when deriving the NNT and NNS. (That is, it is not a binary thing, with one risk below and one risk above the threshold, but rather a continuum of risks). Please clarify.

Response: For the NNS and NNT estimation, we used individuals’ dichotomised risk, i.e., stratifying each individual to be above the threshold as at high risk and those below the threshold as at low risk. The reason for this is we are calculating the NNS and NNT of the risk prediction model **in combination with** the risk threshold. We have clarified this in the text (Page 11, Lines 2-4):

“*We quantified the public health impact of the combined risk prediction model and the stratification rule by the numbers needed to screen (NNS) and the numbers needed to treat (NNT) to prevent one new CVD event in 10 years,…”*

The details of the NNS and NNT calculation can be found in the **Supplementary Methods 5** as follows:

NEPP = Number of individuals who had CVD over the next 10 years and exceeded statin treatment threshold (i.e., high-risk people among the cases) (N) × Proportion who adhere to treatment (Pa) × Relative risk reduction (RRR) of CVD risk associated with statins[13]
The number needed to screen (NNS) to prevent one new CVD event = Number of target population / NEPP
The number needed to treat (NNT) to prevent one new CVD event = Number of high-risk individuals / NEPP

6) The 'calibration (visually assessing the agreement of observed risk and predicted risk by deciles of predicted risk' – I think it is more correct to say tenths of predicted risk.

Response: Done. We have corrected "by deciles of predicted risk" to "by tenths of predicted risk" throughout the main manuscript and supplementary material.

7) The calibration plot does not provide 95% CIs around each point (perhaps because the data are so big?) – was the competing event of death accounted for? This is particular important in the older age groups. Are we assessing calibration in a world where no-one can die (deaths are censored) or in a real world where CVD may not occur because death from other causes happens first?

Response:
Regarding the 95% CIs: these are very narrow (e.g., the observed risk at 10 years for the bottom tenth of the predicted risk for men = 0.0705, with 95% CI = 0.0677 to 0.0734, based on data from one of the five imputation sets) and are not visible on the calibration plot, thus have not been included. Instead, our calibration plots follow a similar format to the published QRISK2 calibration plots [1].

Regarding accounting for competing risks in the calibration plots: this was achieved in the calibration plots for SCORE2, since the original SCORE2 algorithm accounts for competing risks of non-CVD death [14,15]. Here the observed 10-year CVD risk in SCORE2 was estimated using cumulative incidence function (CIF) at 10 years which adjusted for non-CVD death as described in the **Supplementary Methods 1** as follows:

"For SCORE2 calibration, since the predicted risk was estimated accounting for competing risks of non-CVD death, the observed 10-year CVD risk in SCORE2 was estimated using cumulative incidence function (CIF) at 10 years which adjusted for non-CVD death. Thus, all predictive performance measurements for SCORE2 were calculated accounting for censoring."

However, for the calibration plots for the QRISK2 risk estimation, since the original QRISK2 algorithm did not account for the competing risk of non-CVD death,[1] we did not adjust for competing risk for the calibration estimation. Instead, calibration was assessed by comparing the predicted risk based on QRISK2 algorithm with the observed risk.
Note that competing risks has been accounted for in all calculations for CVD-free life expectancy (details in **Supplementary Methods 6**).

8) Ideally calibration curves should be added to the calibration plots (e.g. using pseudo observations), but this is a minor point and not essential for this paper.

Response: Thank you for this suggestion. Given that calibration is not a crucial focus for this study, we have not added the calibration curves.

VERSION 3 – REVIEW

**REVIEWER**	Riley, Richard; University of Birmingham, Institute of Applied Health Research. Competing Interest: None
**REVIEW RETURNED**	26-Jan-2024

GENERAL COMMENTS	I would like to thank the authors for their very clear and considered response to my comments, which I am very happy with. Just a few final comments<br><br>In the 'how his might affect research, practice and policy' part – I think the authors should also mention that the benefits needs to be formally weighted against the costs of treating younger people for longer.<br><br>The AUROC-dp still confuses me (all ROC plots have to dichotmise predictions at the threshold corresponding to the point on the curve, so not sure why this is different here), but I am pleased the authors have added more details<br><br>Figure 1 – this must be the standardised NB? Otherwise I would not expect it to be close to 1. And if so, is it standardised by a different prevalence according to the age on the x axis?

VERSION 3 – AUTHOR RESPONSE

Reviewer: 1

Prof. Richard Riley, University of Birmingham

Comments to the Author

I would like to thank the authors for their very clear and considered response to my comments, which I am very happy with. Just a few final comments.

In the 'how his might affect research, practice and policy' part – I think the authors should also mention that the benefits needs to be formally weighted against the costs of treating younger people for longer.

Response: Thanks for reviewing the revised manuscript and thanks for the comment. We have added the text "The benefits need to be formally weighted against the costs of treating younger people for longer."

The AUROC-dp still confuses me (all ROC plots have to dichotmise predictions at the threshold corresponding to the point on the curve, so not sure why this is different here), but I am pleased the authors have added more details

Response: The main difference is that the AUROC-dp accounts for the time-to-event/censoring in the observed data (**Supplementary Methods 3**). We agree that it can be interpreted as the C-statistic, although the way we calculated aligns better with the C-index - calculated as the proportion of all possible concordant pairs plus half the proportion of ties while taking into account the time-to-event nature of the data (using somersd package in Stata software with time-to-event/censoring included in the calculation).

Figure 1 – this must be the standardised NB? Otherwise I would not expect it to be close to 1. And if so, is it standardised by a different prevalence according to the age on the x axis?

Response: The NB is not standardised (i.e., it has not been divided by its standard deviation).

The net benefit (NB) has been calculated at each age from the equation: NB = true positive rate – (false positive rate * p/(1-p)), and p is the threshold probability (**Supplementary Methos 4**). Thus for older ages, p=0.1 and p/1-p = 0.111.

Furthermore, for older ages, because the majority will have a risk over the threshold, the true positive rate (probability that those with a CVD event will have a risk over the threshold = sensitivity shown in **Supp Figure 8**) approaches 1. Similarly, the false positive rate (probability that those without a CVD event will have a risk over the threshold = (1-specificity), where specificity is shown in **Supp Figure 8**) also approaches 1. Therefore the NB approaches 1-0.11 = 0.889 as shown in Figure 1. (The true positive rate and false positive rate were calculated accounting for time-to-event data, detailed in Supplementary Methods 4).

(Note we have corrected the notation for the algorithm by removing the redundant "N is the total number of the population at each group" in the text in **Supplementary Methods 4**, because we used true positive rate and false positive rate in the calculation).
